# The Vibrational Raman Optical Activity of β‑Propiolactone Derivatives Computed with Coupled-Cluster Theory

**DOI:** 10.1021/acs.jpca.6c01773

**Published:** 2026-09-04

**Authors:** Mya S. Pederson, Luther D. Crum, Ethan J. Poncelet, Brendan M. Shumberger, T. Daniel Crawford, Rollin A. King

**Affiliations:** † Department of Chemistry, 7560 Bethel University, Saint Paul, Minnesota 55112, United States; ‡ Department of Chemistry, 1757 Virginia Tech, Blacksburg, Virginia 24060, United States

## Abstract

Vibrational Raman optical activity (VROA) is an information-rich technique with which to explore the molecular stereochemistry of chiral molecules. The molecular properties needed to model a VROA spectrum can be computed using coupled-cluster (CC) theory. We report VROA spectra computed with CC theory in an origin-independent manner for the first time, applying the approach to several derivatives of β-propiolactone (oxetan-2-one), a strained, four-membered heterocycle with naturally occurring derivatives exhibiting a variety of stereochemistries. The spectra were theoretically benchmarked in several ways. First, a comparison of VROA spectra computed using CC2 theory was made to evaluate the performance of a large number of one-electron basis sets. Second, spectra computed with CC2 and CCSD were compared to gauge the effectiveness of the less costly CC2 method. Third, VROA spectra determined from CC2 chiroptical tensors computed using different geometries and vibrational Hessians were compared with one another. Finally, VROA spectra for β-propiolactones substituted with methyl, ethyl, and propyl groups were produced with CC2 using geometries and Hessians determined by B3LYP/aug-cc-pVTZ computations. For structures monosubstituted at the 3-position (adjacent to the carbonyl), an intense peak occurs at ≈1130 cm^–1^ corresponding largely to the C–C stretch between the ring and the alkyl substituent. For structures monosubstituted at the 4-position, a similar peak corresponding largely to the C–C stretch between the ring and the substituent appears at ≈1115 cm^–1^. For disubstituted species of (3*S*,4*R*) stereochemistry, a mode involving ring-breathing motion and asymmetrical wagging of the H atoms on C3 and C4 (on the same side of the β-lactone ring) produces a large negative signal at ≈1130 cm^–1^. The overall spectrum is found to be dominated by the configuration at the 3-position and less sensitive to that at the 4-position. The results suggest that VROA can be productively employed to interrogate the stereochemistry at the 3-position. Unfortunately, no clear VROA signal was identified that characterizes the stereochemistry at the 4-position in disubstituted species.

## Graphical abstract

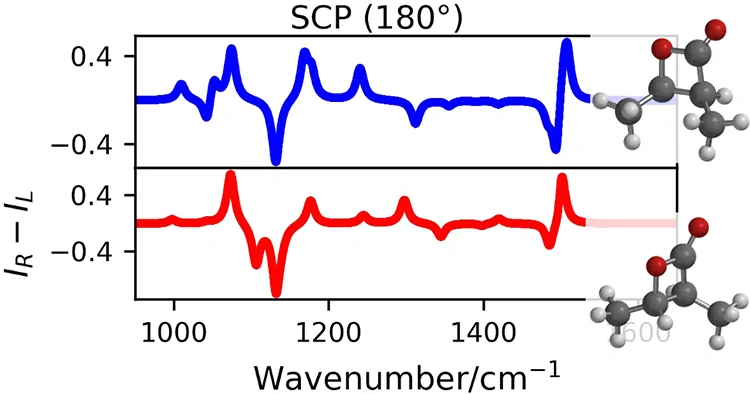

## Introduction

Among the suite of tools available to investigate molecular stereochemistry, Vibrational Raman Optical Activity (VROA) stands out as “richest in structural detail”.^1,2^ VROA is the differential scattering of left- and right-circularly polarized light that induces vibrational excitation due to chirality. Additionally VROA offers the potential to characterize the configuration of chiral centers (or larger molecular motifs) via the spectroscopic behavior of local vibrational modes.

Several experimental approaches are possible. Early experiments carried out “incident circular polarization” (ICP) in which the polarization of the incident photon is varied. Photons scattered at right angles were detected if polarized parallel to, or alternately, perpendicular to, the scattering plane. By 1991, ICP as well as “dual-circular polarization” (DCP), in which both the incident and the scattered photon handedness are varied synchronously, were reported on (−)-trans-pinane.^3^ Currently, however, the most common approach is that of “scattered circular polarization” (SCP) in which the input light is unpolarized, while the intensity of the scattered light is measured separately for right- and left- circularly polarized light. There is further the choice of scattering angle. Backscattering [Δ(180°)] was shown to be advantageous by Hug,^4^ and SCP backscattering has since become the standard approach.

The history of VROA, and particularly its successful application to biomolecules has been reviewed by Barron.^5^ The theory extends back to Barron and Buckingham in 1971.^6^ In the far-from-resonance approximation, VROA is dependent on the geometric derivatives of the electric-dipole polarizability α_*ij*_, the electric-dipole/magnetic-dipole polarizability *G*_*ij*_^′^, and the electric-dipole/electric-quadrupole polarizability *A*_*ijk*_, where *i*, *j*, and *k* represent Cartesian coordinates. These tensors are dependent on molecular orientation. The two Raman invariants are as follows.

1 $$\alpha =\frac{1}{3}\alpha_{\mathrm{ii}}$$

2 $$\beta {\left(\alpha \right)}^{2}=\frac{1}{2}\left(3\alpha_{\mathrm{ij}}\alpha_{\mathrm{ij}}-\alpha_{\mathrm{ii}}\alpha_{\mathrm{jj}}\right)$$

The three VROA invariants are

3 $$\alpha G^{\prime }=\frac{1}{9}\alpha_{\mathrm{ii}}G_{\mathrm{jj}}^{\prime }$$

$$\beta {\left(G{\;}^{\prime }\right)}^{2}=\frac{1}{2}\left(3\alpha_{\mathrm{ij}}G_{\mathrm{ij}}^{\prime }-\alpha_{\mathrm{ii}}G_{\mathrm{jj}}^{\prime }\right)$$ 4

5 $$\beta {\left(A\right)}^{2}=\frac{1}{2}\omega \alpha_{\mathrm{ij}}{\text{ϵ}}_{\mathrm{ikl}}A_{\mathrm{klj}}$$

where ϵ is the Levi–Civita symbol, and summation over repeated indices is implied.^1^ The VROA signal for a particular vibrational mode is determined from the derivative of the tensors in eqs 3–5 with respect to that normal mode. As a consequence, it can be advantageous when using a finite-difference approach to the derivatives to displace along the normal modes of only a desired subset of the full molecular vibrations. Also, for potential interpretive value, the implicit dot products of derivatives in eqs 3–5 can be decomposed into contributions from atomic pairs. The code developed and made public as part of the present work provides both of these extensions (see Methods section).

The relative weighting of the components of the VROA signal varies by experimental setup^1,7^ A typical modern experiment applies a 532 nm input wavelength, detects SCP backscattering [Δ(180°)], and focuses on the 1000–1800 cm^–1^ range. In such cases, the unitless circular intensity differential (Δ) can be expressed as follows

$$\Delta \left(180^{{}^{\circ}}\right)=\frac{I_{R}-I_{L}}{I_{R}+I_{L}}=\frac{\frac{32}{c}\left[3\beta {\left(G{\;}^{\prime }\right)}^{2}+\beta {\left(A\right)}^{2}\right]}{4\left[45\alpha^{2}+7\beta {\left(\alpha \right)}^{2}\right]}$$ 6

where the numerator is proportional to the circular intensity difference activity and the denominator is proportional to the Raman activity.

The needed molecular properties are readily expressed in a sum-over-states form, but in practice they are usually determined via linear-response equations.^8,9^ In 2011 Crawford and Ruud^10^ demonstrated the first VROA spectra obtained using frequency-dependent linear-response coupled-cluster (LR-CC) theory, providing both an alternative to the commonly deployed DFT approach, as well as the possibility of rigorous testing of DFT predictions. Although challenges to agreement with experiment abound (e.g., the effects of anharmonicity, solvation, and dynamical motion), experience with other chiroptical properties suggests that careful evaluation of the electronic structure treatment itself is warranted.

Given that the computation of a VROA spectrum requires a molecular geometry, vibrational normal modes and frequencies, and derivatives of chiroptical tensors along these modes, VROA provides an excellent use case for modularity and interoperability in quantum chemistry calculations (see Methods section). However, VROA intensities can be obtained without going beyond the Born–Oppenheimer approximation as required and recently achieved for dynamically correlated wave function methods for VCD.^11–13^

Several studies have been published on the basis-set dependence of VROA predictions using density functional theory.^14–16^ The primary conclusions were that accurately computing the force field is of great importance, and that smaller basis sets are adequate for computing the polarizability tensors. For example, property computations with aug-cc-pVDZ and aug-cc-pVTZ gave “similar”^15^ or “nearly equivalent”^16^ results. Diffuse functions of *d* type were found to be expendable, and the resulting aug­(sp)-cc-pVDZ basis or the rDPS basis set^17^ were recommended.

A recent study by Shumberger et al.^18^ compared the VROA and VCD predictions (with the DFT functionals B3LYP and CAM-B3LYP) for a variety of basis sets as large as aug-cc-pVQZ for 6 molecules. The final evaluative summary was to roughly categorize the basis set performance as “poor” (rDPS, aug-D-3-21G, augT3-3-21G, R-ORP), “better” (aug-cc-pVDZ, Sadlej-PVTZ), “good” (ORP, Lpol-ds, Lpol-dl, Lpol-fs) and “best” (Lpol-fl, aug-cc-pVTZ). These basis sets were also evaluated in this paper with coupled-cluster theory.

β-propiolactone (oxetan-2-one) derivatives (e.g., **3** and **4** in Figure 1) are highly strained, four-membered organic heterocycles with a high degree of electrophilicity and reactivity. The characteristic ring is susceptible to nucleophilic attack. These compounds exhibit pharmaceutical potential, as numerous related natural products have already been shown to have bioactivity against bacteria, fungi, cancer, and diabetes.^19^

**Figure 1:**
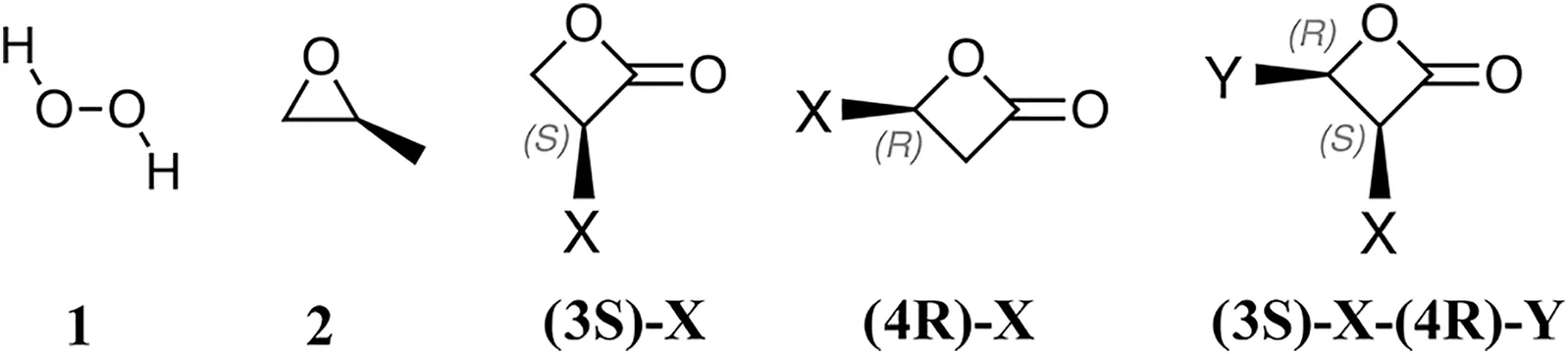
Molecules investigated in this study include hydrogen peroxide (**1**) and (*S*)-methyloxirane (**2**). With **X** and **Y** as methyl groups (**m**), (3*S*)-3-methyloxetan-2-one [**(3*S*)-m**], (4*R*)-4-methyloxetan-2-one [**(4*R*)-m**] and (3*S*,4*R*)-3,4-dimethyloxetan-2-one [**(3*S*)-m-(4*R*)-m**] are shown. Analogues with ethyl (**e**) and propyl (**p**) were also explored.

β-propiolactones are synthetic targets^20–22^ not only for their inherent biological activity, but also for the ways in which their ring strain and chirality may be exploited in synthesis.^23^ Ring-opening decarboxylation, rearrangement, and other reactions have been widely employed.^24–27^ As one example among many, Ito et al.^28^ introduced flourine substitution of β-propiolactones to generate a “building block” that was used to synthesize fluorine-containing chiral β-hydroxy aryl ketones.

Mensch, Johannessen, and co-workers have carried out several computational studies of the VROA spectra of model peptides^29–31^ examining questions such as the effect of explicit water hydrogen-bonding, the impact of amino acid side chains, and the robustness of bands proposed as indicators of a β-turn. In a similar spirit, we present here an initial search for characteristic bands in VROA spectra that could provide information on the configuration of stereocenters within a β-propiolactone ring. The substituent variation from methyl-, to ethyl-, to propyl-, was chosen to generate analogues that were computationally tractable with coupled-cluster (CC) theory, and limited in chemical variety. The presence of stereochemistry-indicating VROA modes robust to such a modest change as the length of an alkyl substituent would seem a necessary (but not sufficient) requirement for them to apply to more complex molecules. Smaller substituents also minimize the complication of conformational flexibility. If characteristic signals are identified, then additional study—theoretical and experimental—could establish whether the bands were applicable to larger analogues of synthetic or biological significance.

Luber^32^ has introduced DFT-based molecular dynamics to the calculation of VROA allowing for the inclusion of dynamical effects and natural line broadening. A potential advantage of β-propiolactones as a computational target is that the rigid nature of the ring structure may prevent dramatic conformational effects on the VROA spectra.

In this work, CC predictions of VROA spectra are evaluated in several ways. The VROA spectra for (*S*)-methyloxirane (**2**) computed with a plethora of one-electron basis sets are compared. The results are compared with the gas-phase experimental spectrum. For several substituted β-propiolactones, spectra computed with CC2 ^33^ are compared with those determined with the more complete CCSD theory. The significance of the chosen geometry and Hessian is explored. Following these preliminaries, the VROA spectra of small mono- and di- substituted β-propiolactone derivatives are reported. An analysis reveals promising characteristic similarities in the VROA spectra that might be generalizable to larger natural compounds, and thereby aid in the identification of their stereochemistry.

**Figure 2:**
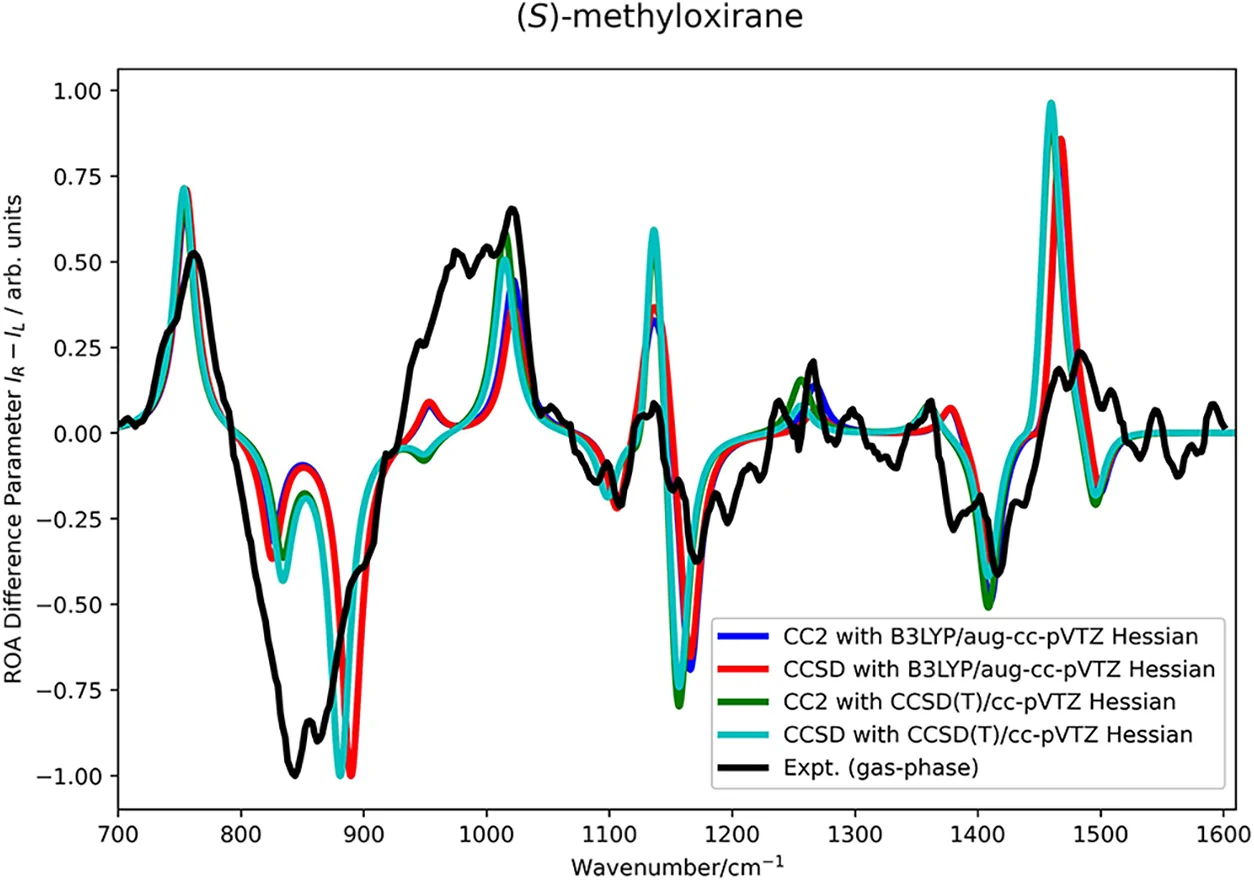
CC2/aug-cc-pVDZ and CCSD/aug-cc-pVDZ VROA parameters of (*S*)-methyloxirane computed with both B3LYP/aug-cc-pVTZ and CCSD­(T)/cc-pVTZ geometry and Hessian. The gas-phase experiment is from ref 61. Harmonic frequencies have been decreased by 2% for B3LYP and 4% for CCSD­(T). Each data set has been separately scaled to a maximum magnitude of 1.0.

## Methods

The following basis sets were used, listed (roughly) in order of increasing size: cc-pVDZ,^34^ 6-31+G*,^35–37^ augD-3-21G,^38^ augT3-3-21G,^38^ asp-cc-pVDZ, which is jun-cc-pV­(D + d)­Z^39^ augmented by the inclusion of diffuse *s* and *p* functions from aug-cc-pVDZ on the hydrogen atoms, R-ORP,^40^ aug-cc-pVDZ,^34,41^ Sadlej-pVTZ,^42^ ORP,^43^ d-aug-cc-pVDZ,^34,41,44^ LPol-ds,^45^ LPol-dl,^45^ LPol-fs,^45^ aug-cc-pVTZ,^34,41^ LPol-fl,^45^ and aug-cc-pVQZ.^34,41^ Compared to the collection of basis sets recently used to test VCD and VROA spectra with DFT by Shumberger et al.,^18^ the present list excludes rDPS,^17^ while adding cc-pVDZ, 6-31+G*, asp-cc-pVDZ, and d-aug-cc-pVDZ.

A Python module named Roa^46^ was developed to manage the workflow and conveniently carry out all the steps of a VROA spectrum computation. Frequency-dependent coupled-cluster (CC2 and CCSD) linear response theory implemented in Psi4 ^10^ was used to compute the perturbed wave functions, the response functions, and the molecular property tensors. Gauge-including atomic orbitals (GIAOs) were not used in this study, however, the CC linear-response calculations used the modified velocity gauge representation for the electric dipole operator, so the results are origin independent. (This represents an improvement from the only previously published CC VROA results^10^ which used the length representation and thus contained some origin dependence.) The derivatives of these tensors with respect to the vibrational normal modes were determined by finite-differences of nuclear displacements, so that the total computation is rather complicated. Running these computations, collecting the results, and computing the VROA properties was managed using the Python module, which takes advantage of the Python API (Application Programming Interface) of Psi4,^47^ as well as some program interoperability. The API readily supports complex input files written in Python to perform nonstandard computations utilizing portions of the Psi4 code base. The B3LYP^48–50^ optimizations and frequency computations were carried out with Psi4, while the CC vibrational frequencies and normal modes were computed using the CFOUR program.^51^ The CC geometry optimizations were carried out using CFOUR’s gradients provided to Psi4’s geometry optimization tools.

Provided tables and figures present SCP peaks computed with an input wavelength of 532 nm. The values of the distinct VROA invariants are included in the SI for all structures. These values enable the reproduction of any of the model spectra presented here, or those corresponding to other VROA experimental setups.^1,7^

In figures, unless otherwise specified, the spectra are presented assuming Laplacian line widths with FWHM = 0.001 eV, or HWHM = 4.033 cm^–1^. The spectral plots comparing CC2 with CCSD results are unscaled unless otherwise noted. All other spectral plots in the figures show relative intensity for which the VROA peak of greatest magnitude is assigned a value of ±1, and all other peaks are correspondingly scaled. In accordance with many other theoretical VROA papers, and to maximize reproducibility, we have chosen to omit from the modeled spectra the ν^4^ and Boltzmann factors suitable for direct comparison to experimental spectra.^8,16^ The values corresponding to the numerator and denominator of eq 6 are labeled “*I*_R_ – *I*_L_” and “*I*_R_ + *I*_L_”, respectively, in the provided figures and tables. Since experimental parameters such as incident laser intensity (which cancel in the quotient) are neglected, the values presented technically correspond to the Raman circular intensity difference activity and the Raman activity, respectively.

**Figure 3:**
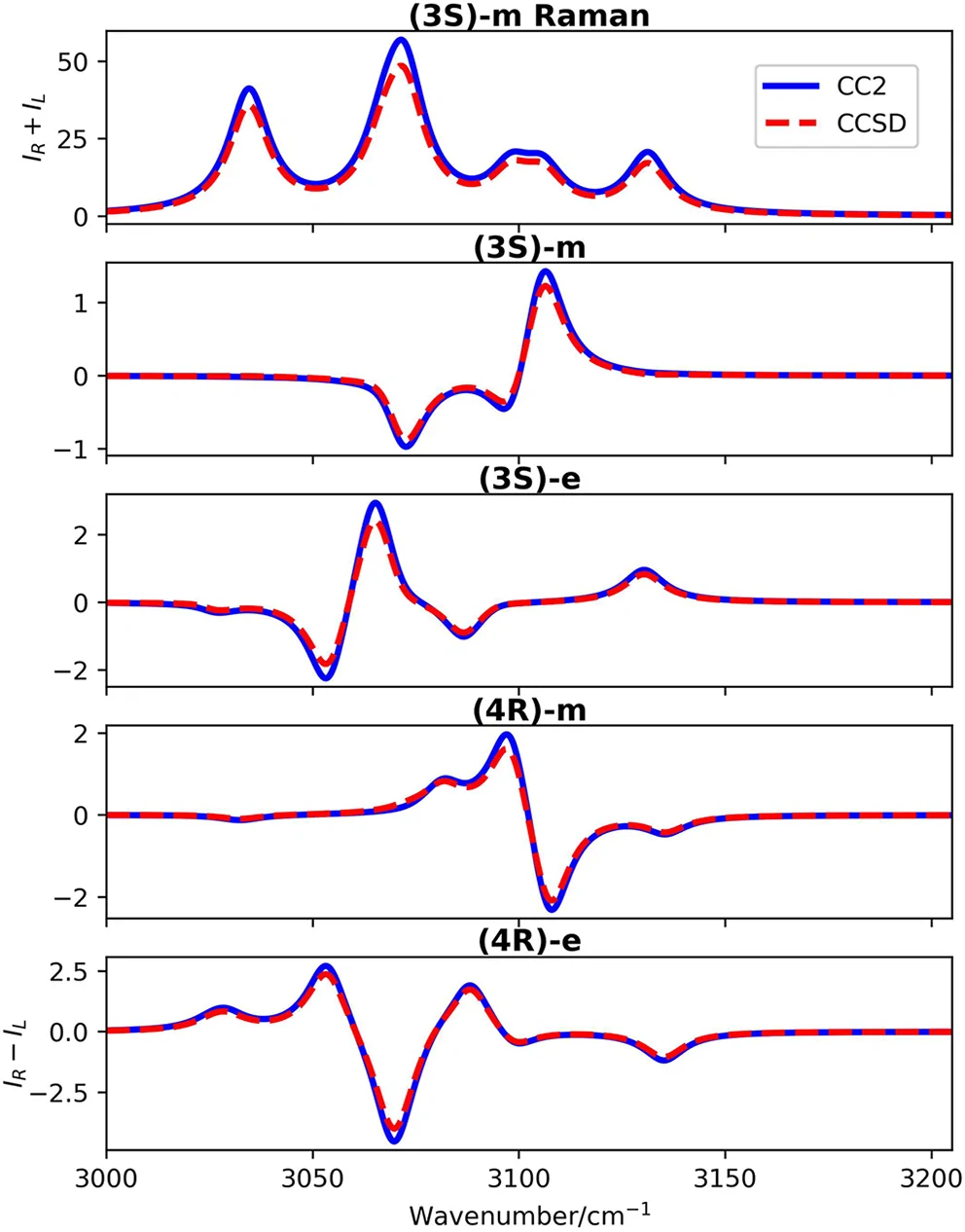
SCP­(180°) Raman (top) and VROA (lower four panels) spectra of **3** and **4** in the high-frequency range. The geometry and Hessian were computed with B3LYP/aug-cc-pVTZ, the CC chiroptical tensors with the asp-cc-pVDZ basis set. Units of Å^4^amu^–1^ for Raman, 10^–3^Å^4^amu^–1^ for VROA. Values here and in other figures correspond to the Raman circular intensity difference activity and Raman activity (see Methods section).

**Figure 4:**
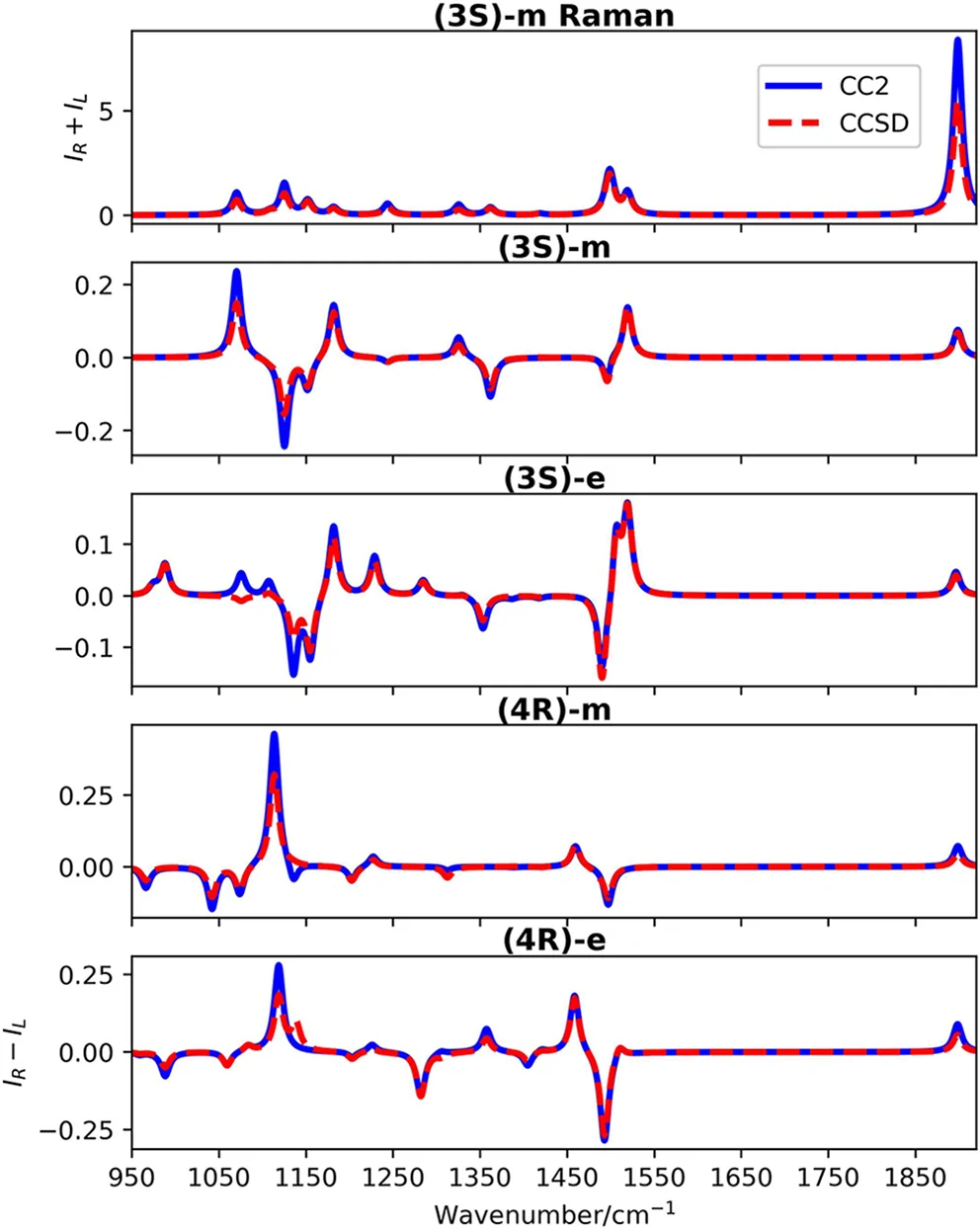
SCP­(180°) Raman (top) and VROA (lower four panels) spectra of **3** and **4** in the midfrequency range. The geometry and Hessian were computed with B3LYP/aug-cc-pVTZ, the CC chiroptical tensors with the asp-cc-pVDZ basis set. Units of Å^4^ amu^–1^ for Raman, 10^–3^ Å^4^ amu^–1^ for VROA.

The developed Python module provides several potentially useful features for analysis of VROA spectra for which we do not report extensive application here. First, the VROA signal along any vibrational mode can be decomposed into contributions from atomic pairs. This type of analysis allows for the investigation of the role of nearby atoms such as strongly vibrating hydrogen atoms.^52^ Yamamoto et al.^53^ investigated the transferability of molecular property tensors including those used in VROA spectra. For VROA, parameter transfer was successful for qualitative interpretation of spectra given reasonably sized fragments. For example, their conclusion mentions the (Pro)_4_ → (Pro)_20_ transfer. The atomic pair decomposition reduces all the way to atoms and is thus unlikely to be usable to determine parameters in larger molecules. However, pair contributions could assist in qualitative interpretation in certain cases. Early work in this area was done by Hug^54^ who wrote that although decomposition of a chiroptical property “into truly atomic terms is at any rate a hopeless undertaking. As shown here for circular difference scattering cross sections, a meaningful subdivision of this chiroptical property into additive quasi-atomic quantities” useful for visualization is possible.

Second, two robustness parameters were proposed by Tomassini et al.,^55^ including the ψ “angle” between *Ã*_*ij*_ = ϵ_*ikl*_ *A*_*klj*_ and α, as well as ϕ which is defined by the inner product of α and *G*′. We implemented the calculation of these parameters and found that a plot of β­(*A*)^2^ vs cos­(ψ) for all normal modes in a small β-lactone presents two distinct linear relationships—one corresponding to C–H stretching modes, and one to all other modes. A similar plot can be produced from β­(*A*)^2^ vs cos­(ϕ), but we do not further explore these robustness parameters here.

### Similarity Metrics

The similarity of two spectra is computed here using five different metrics, whose values are independent of any common, constant prefactor. The first three metrics were also used by Shumberger et al.^18^ recently. The five are the singly normalized overlap (SNO), the double normalized overlap (DNO) also referred to as “Carbo”,^56,57^ the integrated difference function (IDF), the relative absolute difference function (RADF), and the modified Jaccard (MJAC) function.^56,58^ They are defined by the following expressions, where each integration is over a chosen spectral range.

7 $$\mathrm{SNO}\left(f_{1},f_{2}\right)=\frac{\int f_{1}f_{2}}{\int {f_{2}}^{2}}$$

8 $$\mathrm{DNO}\left(f_{1},f_{2}\right)=\frac{\int f_{1}f_{2}}{\sqrt{\int {f_{1}}^{2}\cdot \int {f_{2}}^{2}}}$$

9 $$\mathrm{IDF}\left(f_{1},f_{2}\right)=\frac{\int f_{2}^{2}-\int f_{1}^{2}}{\int f_{2}^{2}}$$

10 $$\mathrm{RADF}\left(f_{1},f_{2}\right)=\frac{\int \left|f_{1}-f_{2}\right|}{\int \left|f_{2}\right|}$$

11 $$\mathrm{MJAC}\left(f_{1},f_{2}\right)=\frac{\int f_{1}f_{2}}{\int {f_{1}}^{2}+\int {f_{2}}^{2}-\left|\int f_{1}f_{2}\right|}$$

The function *f*_2_ is treated as the “reference function” in cases where the better answer is clear. For example, when evaluating basis sets, the spectrum of the largest (most complete) basis set serves as the reference. When comparing CCSD with CC2, the CCSD spectrum serves as the reference. The SNO, IDF, and RADF are dependent on the selection of the reference function, so in cases where the selection of reference is unclear, we introduce the following symmetric forms.

12 $${\mathrm{SNO}}^{\prime }\left(f_{1},f_{2}\right)=\frac{1}{2}\left[\mathrm{SNO}\left(f_{1},f_{2}\right)+\mathrm{SNO}\left(f_{2},f_{1}\right)\right]$$

13 $${\mathrm{IDF}}^{\prime }\left(f_{1},f_{2}\right)=\frac{1}{2}\left[\mathrm{IDF}\left(f_{1},f_{2}\right)+\mathrm{IDF}\left(f_{2},f_{1}\right)\right]$$

14 $${\mathrm{RADF}}^{\prime }\left(f_{1},f_{2}\right)=\frac{1}{2}\left[\mathrm{RADF}\left(f_{1},f_{2}\right)+\mathrm{RADF}\left(f_{2},f_{1}\right)\right]$$

For two idealized enantiomers, the SNO, DNO, IDF, RADF and MJAC of VROA spectra are equal to −1, −1, 0, 2, and −1, respectively. In contrast, for two identical spectra the SNO, DNO, IDF, RADF and MJAC are equal to 1, 1, 0, 0, and 1, respectively. These ideal values hold for both the original and symmetrized forms.

## Results and Discussion

### The Electric-Dipole/Electric-Quadrupole Term

As the electric-dipole/electric-quadrupole term is computationally costly, some have investigated whether the term could be neglected. Based on DFT computations, Luber et al.^52^ concluded that the contribution of the electric-dipole/electric-quadrupole tensor is generally small, particularly so below 2000 cm^–1^. Omitting this term at higher frequencies can cause remarkable changes to the VROA signal of the C–H stretching modes.

In 2020, Johnson et al.^59^ reported a comparison of DFT computations with the experimental spectrum of camphor, concluding that several bands related to C–H bending motions were dominated by the electric-dipole/electric-quadrupole contribution, and similarity overlaps indicated significantly better comparison to experiment if the term is included. Additional computations on fenchone and dimethyloxirane supported this conclusion.

As a preliminary comparison with CC theory, we examined the significance of the distinct CC tensor contributions for 101 normal modes of HOOH (CCSD/Sadlej-LPol-fl), methyloxirane (CCSD/aug-cc-pVDZ), and limonene (CC2/6-31+G*). For this set, the β­(*A*)^2^ contribution was found to be large. However, for the vast majority of modes, the β­(*A*)^2^ and β­(*G*′)^2^ terms have the same sign, so that neglect of the former rarely leads to a major error in Δ(180°) backscattering experiments for which the β­(*G*′)^2^ term has a larger coefficient of the same sign. Although these CC results are limited to three molecules, in a study of several terpene molecules Yu et al.^60^ similarly reported the tendency of β­(*A*)^2^ to be smaller and of the same sign as β­(*G*′)^2^. For other types of VROA, e.g., Δ(0°) or Δ(90°)_*z*_, the quadrupole term would often be decisive. For the modes of the three molecules investigated here with CC theory, the opposite sign for Δ(0°) or Δ(90°)_*z*_ is obtained for *most* of the vibrational modes if the β­(*A*)^2^ term is excluded.

### One-Electron Basis Sets for (*S*)-Methyloxirane (**2**)

The VROA spectrum for (*S*)-methyloxirane (**2**) was computed using a large number of distinct one-electron basis sets. Table 1 presents the number of basis functions and the similarity of each spectrum to that of the aug-cc-pVTZ basis set as measured by five different metrics (see eqs 7–11). In the top half of Table 1, all the chiroptical tensors were computed using the same (aug-cc-pVTZ) reference geometry and vibrational modes. Deviations are due solely to the differences between derivatives along the normal modes of the key tensor quantities in eqs 3–5. In the lower half of Table 1, the comparison is between spectra determined by a fully consistent computation carried out at a single level of theory, including geometry optimization and Hessian. In this case, differences in the computed vibrational frequencies, which locate the VROA peaks, contribute to large spectral differences.

**Table 1:** Similarity of VROA Spectra for Various One-Electron Basis Sets Computed with CC2 and a Fixed Geometry and Hessian (Top) and a Fully Consistent Geometry and Hessian (Bottom)^a^ ^,^ ^b^

(*S*)-Methyloxirane with CC2/aug-cc-pVTZ geometry and Hessian						
basis set	NBF^c^	SNO	DNO	IDF	RADF	MJAC
cc-pVDZ	86	1.2992	0.9768	–0.7693	0.5982	0.8838
6-31+G*	88	1.1807	0.9919	–0.4170	0.3456	0.9550
augD-3-21G	108	0.9759	0.9980	0.0437	0.1109	0.9955
augT3-3-21G	108	1.0202	0.9992	–0.0425	0.0869	0.9979
asp-cc-pVDZ	126	1.0339	0.9991	–0.0707	0.1079	0.9971
R-ORP	146	1.0672	0.9994	–0.1403	0.0940	0.9945
aug-cc-pVDZ	146	1.0462	0.9995	–0.0954	0.0729	0.9970
Sadlej-pVTZ	150	1.0559	0.9996	–0.1159	0.0727	0.9961
ORP	206	1.0141	0.9997	–0.0290	0.0412	0.9992
d-aug-cc-pVDZ	206	1.0212	0.9998	–0.0432	0.0408	0.9992
Sadlej-LPol-ds	222	1.0157	0.9998	–0.0320	0.0366	0.9993
Sadlej-LPol-dl	282	1.0163	0.9998	–0.0332	0.0353	0.9994
aug-cc-pVTZ	322	1.0000	1.0000	0.0000	0.0000	1.0000
Sadlej-LPol-fs	338	1.0086	0.9999	–0.0175	0.0253	0.9997

(*S*)-Methyloxirane with CC2						
basis set	NBF^c^	SNO	DNO	IDF	RADF	MJAC
cc-pVDZ	86	0.7185	0.7296	0.0301	1.2434	0.5742
6-31+G*	88	–0.1442	–0.0983	–1.1501	2.1868	–0.0480
augD-3-21G	108	0.0019	0.0021	0.2352	1.8683	0.0011
augT3-3-21G	108	0.0119	0.0136	0.2288	1.8897	0.0068
asp-cc-pVDZ	126	0.3700	0.4375	0.2850	1.1848	0.2751
R-ORP	146	0.0452	0.0496	0.1693	1.7918	0.0253
aug-cc-pVDZ	146	0.9574	0.8633	–0.2299	0.8802	0.7524
Sadlej-pVTZ	150	–0.0164	–0.0185	0.2173	1.7406	–0.0093
ORP	206	–0.0955	–0.0878	–0.1833	1.6419	–0.0458
d-aug-cc-pVDZ	206	0.8841	0.8761	–0.0183	0.8382	0.7795
Sadlej-LPol-ds	222	0.0524	0.0521	–0.0134	1.4222	0.0267
Sadlej-LPol-dl	282	0.1996	0.2129	0.1206	1.2916	0.1188
aug-cc-pVTZ	322	1.0000	1.0000	0.0000	0.0000	1.0000
Sadlej-LPol-fs	338	0.6358	0.6990	0.1725	0.7525	0.5336

a Comparison of *I*_R_ – *I*_L_ for backscatter (180°) SCP in FFR approximation. Lineshapes assumed to be Lorentzian with a FWHM = 0.001 eV.

b See Methods for definition of each metric. The aug-cc-pVTZ basis set was used as the reference.

c The number of basis functions.

The basis-set performance with CC theory using the aug-cc-pVTZ geometry and Hessian presented in the top half of Table 1 and the corresponding table for HOOH (see SI) jibes with the conclusions of Shumberger et al.^18^ who compared VCD and VROA spectra from DFT computations on six molecules. Those conclusions recommended the ORP and Lpol-ds basis sets, and then as smaller fallback options, aug-cc-pVDZ and Sadlej-pVTZ. However, the wave function computations carried out completely within each basis (bottom half of Table 1) are less consistent with the DFT results. In particular, the aug-cc-pVDZ basis appears to be performing much better than the ORP and smaller Sadlej basis sets. This difference is due to the former being better able to replicate the vibrational frequencies of the reference aug-cc-pVTZ basis set.

Since DFT geometries and frequencies are less sensitive to basis-set size than those from CC computations, the relative results in the bottom half of Table 1 differ substantially from those of the previous DFT analysis (as well as the top half of the table). Variation in the predicted harmonic vibrational frequencies, particularly for wave function methods, will generally lead to poor values in the similarity metrics. We add that significant linear dependencies in the Lpol basis sets larger than Lpol-ds presented a significant obstacle for wave function-based computations on (*S*)-methyloxirane.

The SNO and DNO metrics complement one another as described in ref 18. The choice of a reference function (aug-cc-pVTZ) implies that the SNO metric can have a value larger than 1.0 if the area of the sample spectrum is larger than the area of the reference spectrum. The values in the top half of Table 1 show that this occurs to a degree for most of the less complete basis sets (at least for (*S*)-methyloxirane). The DNO metric neglects differences in peak intensities, so it has values uniformly less than 1.0; however, almost all basis sets give DNO values greater than 0.99. Both the IDF and the RADF metrics are zero for two identical spectra, but like the SNO, the IDF provides information on the (squared) area of the sample spectrum relative to that of the reference. On the other hand, the IDF value is zero for two enantiomers, so its value cannot distinguish between them. While the RADF cannot be negative, either a positive or negative IDF value indicates deviation between the spectra. Tentatively assuming that accuracy improves with the number of basis functions, the RADF metric shows highest correlation and good sensitivity for the purpose of comparing computations of the same quantity. MJAC correlates well with RADF and very closely with DNO.^56^

Previous authors have reported that a high-quality geometry and Hessian are essential for reliable VROA modeling, while significantly smaller basis sets may be used in the computation of the spectral tensors with DFT.^15,16^ Wave function-based methods generally require larger one-electron basis sets than DFT to determine the geometric structure and normal modes of good quality. On the other hand, the CC2 linear response results appear to converge rapidly with respect to basis-set size, and perhaps as quickly as DFT predictions.

For example, for (*S*)-methyloxirane, in comparison to the CC2/aug-cc-pVTZ spectrum, and using the CC2/aug-cc-pVTZ geometry and Hessian, computing the chiroptical tensors with *any* of the basis sets with more than 100 functions performs reasonably well. Furthermore, from a consistent Hessian, the VROA results (particularly as measured by RADF) converge rather smoothly with the number of basis functions.

Since the focus of this section is to evaluate basis-set dependence, the largest basis set was used as the benchmark. The experimental gas-phase VROA spectrum is available for (*S*)-methyloxirane; however, drawing conclusions about basis-set quality by comparing to it would be more tenuous than comparing to the large-basis set results reported here. Comparison to the experimental results follows in the next section.

### Performance of CC2 Versus CCSD

Since the publication of the theory and initial demonstration of the feasibility of carrying out VROA simulations with LR-CC theory,^10^ there has been a dearth of its application to molecules of potential experimental interest. CC2 ^33^ is an approximation to CCSD that formally scales as *N* ^5^, while CCSD scales as *N* ^6^. The lower scaling of CC2 facilitates application to molecules of chemical interest and the large number of computations necessary to determine a VROA spectrum.

Similarity between the CCSD spectrum and the gas-phase experimental spectrum of S̆ebestík and Bour̆^61^ was previously demonstrated by Crawford and Ruud^10^ who concluded, “The overall sign pattern matches well between theory and experiment, with the primary features clearly reproduced”. In Figure 2, we present CC2 and CCSD results, computed using the CCSD­(T)/aug-cc-pVTZ geometry and Hessian of ref 10, as well as with the B3LYP/aug-cc-pVTZ geometry and Hessian. Good agreement is achieved among all the computed spectra, with the B3LYP geometry and Hessian comparing well with the CCSD­(T) benchmark, and CC2 giving almost identical results as that of the more expensive CCSD method. All the computed spectra reproduce the major features of the experimental results. (We also note that we identified a sign error in one component of the ROA invariants in the 2010 CCSD implementation, which we have corrected here. However, this does not alter the overall comparison with experiment.)

CC2 and CCSD VROA spectra were determined for monosubstituted β-propiolactones **3** and **4** with methyl (**m**) and ethyl (**e**) substituents. The geometry and Hessian were computed with B3LYP/aug-cc-pVTZ, and the CC chiroptical tensors with the asp-cc-pVDZ basis set. The spectra are illustrated in the high-frequency and mid-frequency regions in Figures 3 and 4, respectively. In the high-frequency range, the VROA curves are virtually identical, with CC2 showing slightly greater amplitudes than CCSD. In the more complex mid-frequency range, some differences are apparent, particularly in the bending modes around 1100–1150 cm^–1^ (highlighted below), in addition to the aforementioned tendency toward greater CC2 amplitudes. Overall, there is encouraging agreement between the CC2 and CCSD spectra. Given the larger size of some of the β-lactone analogues studied below, along with the large number of required perturbed wave function computations necessary to produce a VROA spectrum, the CC2 approach was used for the remainder of the β-lactone investigation.

### Monosubstituted β-Propiolactones

VROA spectra with CC2/asp-cc-pVDZ were determined for monosubstituted β-propiolactones **3** and **4** with methyl (**m**), ethyl (**e**), and propyl (**p**) substituents, respectively. The results are presented for the (3*S*) and the (4*R*) stereoisomers, respectively, which are oriented upward or above the ring as depicted in Figure 1.

The midrange VROA spectra for monosubstituted **3*S*** are shown in Figure 5, and information on the most prominent peaks is listed in Table 2. The spectra are qualitatively similar for the different, albeit chemically similar, alkyl substituents. The similarity is quantified in the final section. In particular, there is a large negative signal at ≈1130 cm^–1^. Analysis of this normal mode shows that it corresponds largely to a ring breathing motion coupled with a stretching motion of the C–C bond between the ring and the substituent. A second notable peak has a positive value, occurs at ≈1519 cm^–1^, and corresponds with HCH scissoring motion at C4 as well as the substituent.

**Figure 5:**
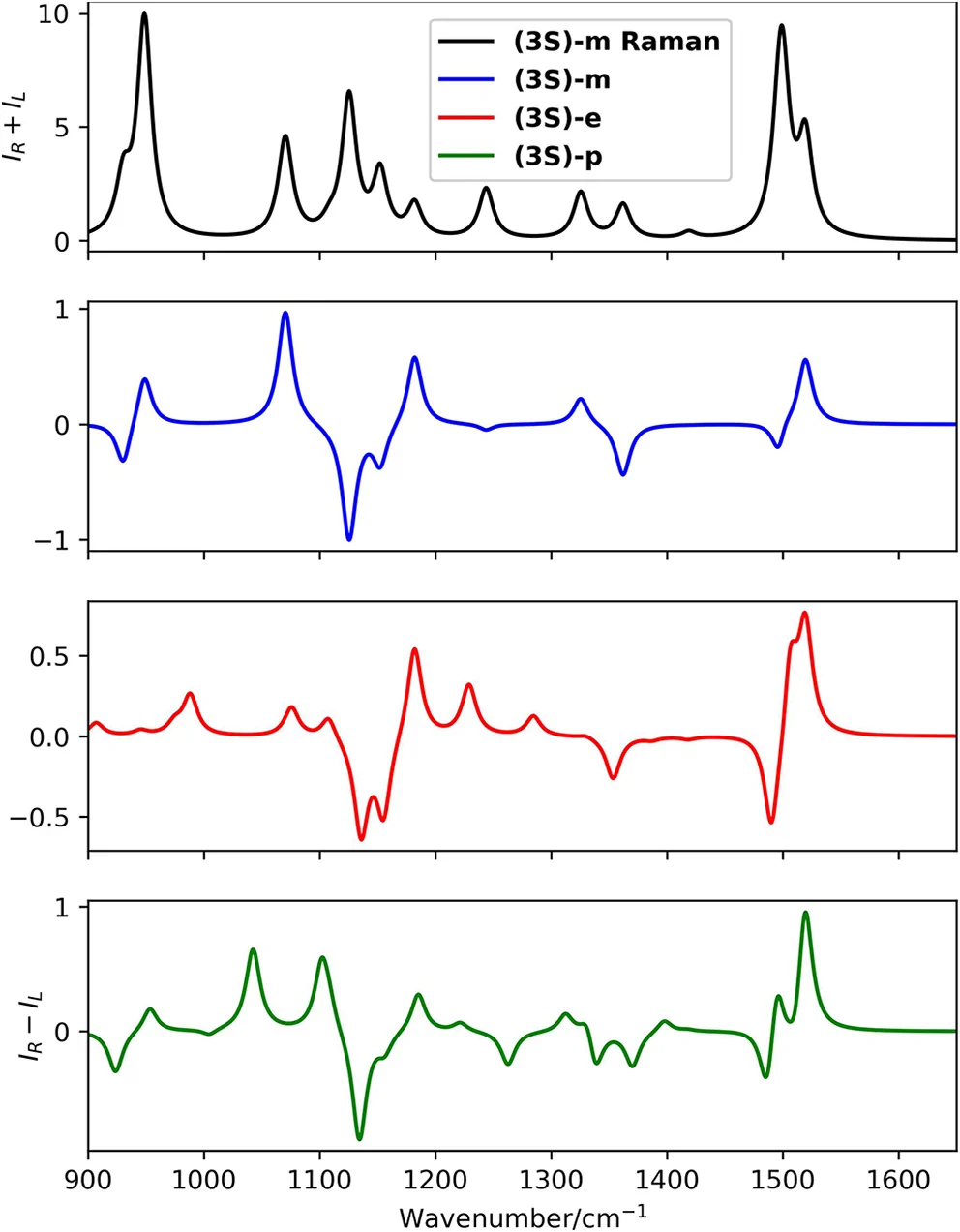
SCP­(180°) Raman (top) and VROA (lower three panels) spectra of **3*S*** in the mid-frequency range computed with CC2/asp-cc-pVDZ using the B3LYP/aug-cc-pVTZ geometry and Hessian. Arbitrary unit (unit increased by 10^3^ for Raman).

**Table 2:** Vibrational Modes with Largest SCP(180°) ROA Difference Parameters in the Mid-frequency Range for (3*S*)-X-2-Oxetanone Determined by CC2/asp-cc-pVDZ Using a B3LYP/aug-cc-pVTZ Geometry and Hessian

mode	ω/cm^–1^	*I*_R_ – *I*_L_ ^a^
**(3*S*)-m**		
ν_8_ C–H^c^ on C4	1519.4	2.6421
ν_12_ C–C btw C4, C3, and **m**	1361.8	–2.0463
ν_15_ C–H^b^, C–C btw C3 and **m**	1181.8	2.8098
ν_17_ ring breathing, C–C btw C3 and **m**	1125.3	–4.5904
ν_19_ C2–O1, C–H^d^ on C3 and **m**	1070.3	4.5180
**(3*S*)-e**		
ν_10_ C–H^c^ on C4, also on **e**	1519.4	3.1417
ν_11_ C–H^c^ on **e**, also on C4	1505.9	2.9403
ν_13_ C–H^c^ on **e**, also on C4	1490.1	–2.8954
ν_20_ C–H^b^ on C4,C–C btw C3 and **e**, C–H^d^ on **e**	1181.7	2.6629
ν_22_ ring breathing, C–C btw C3 and **e**	1135.7	–2.7796
**(3*S*)-p**		
ν_12_ C–H^c^ on C4, also on **p**	1519.2	4.9455
ν_15_ C–H^c^ on **p**	1495.3	2.5371
ν_16_C–H^c^ on **p**, also on C4	1486.3	–2.6539
ν_27_ ring breathing, C–C btw C3 and **p**, C–H^d^ on **p**	1134.2	–4.1282
ν_30_ C–C on **p**	1042.3	2.9965

a Units of 10^–3^ Å^4^ amu^–1^. Values here and in other tables correspond to the Raman circular intensity difference activity (see Methods section).

b Rocking.

c Scissoring.

d Wagging.

The midrange VROA spectra for monosubstituted **4*R*** are shown in Figure 6, and information on the most prominent peaks is listed in Table 3. Again the spectra show qualitative similarity across the alkyl groups. For the **4*R*** species, a large positive signal presents at ≈1115 cm^–1^. Once again, this normal mode corresponds with a ring breathing motion coupled with a stretching motion of the C–C bond between the ring and the substituent—this time on C4. The second largest common peak is a negative VROA intensity at just under 1500 cm^–1^ corresponding with C–H scissoring on the substituent. Thus, intense peaks related to similar vibrational motions of the monosubstituted **3*S*** and **4*R*** structures have opposite signs in the VROA spectrum. The distinct results raise the question, explored in the next section, whether or not the stereochemistry of disubstituted analogues may also be readily distinguished.

**Figure 6:**
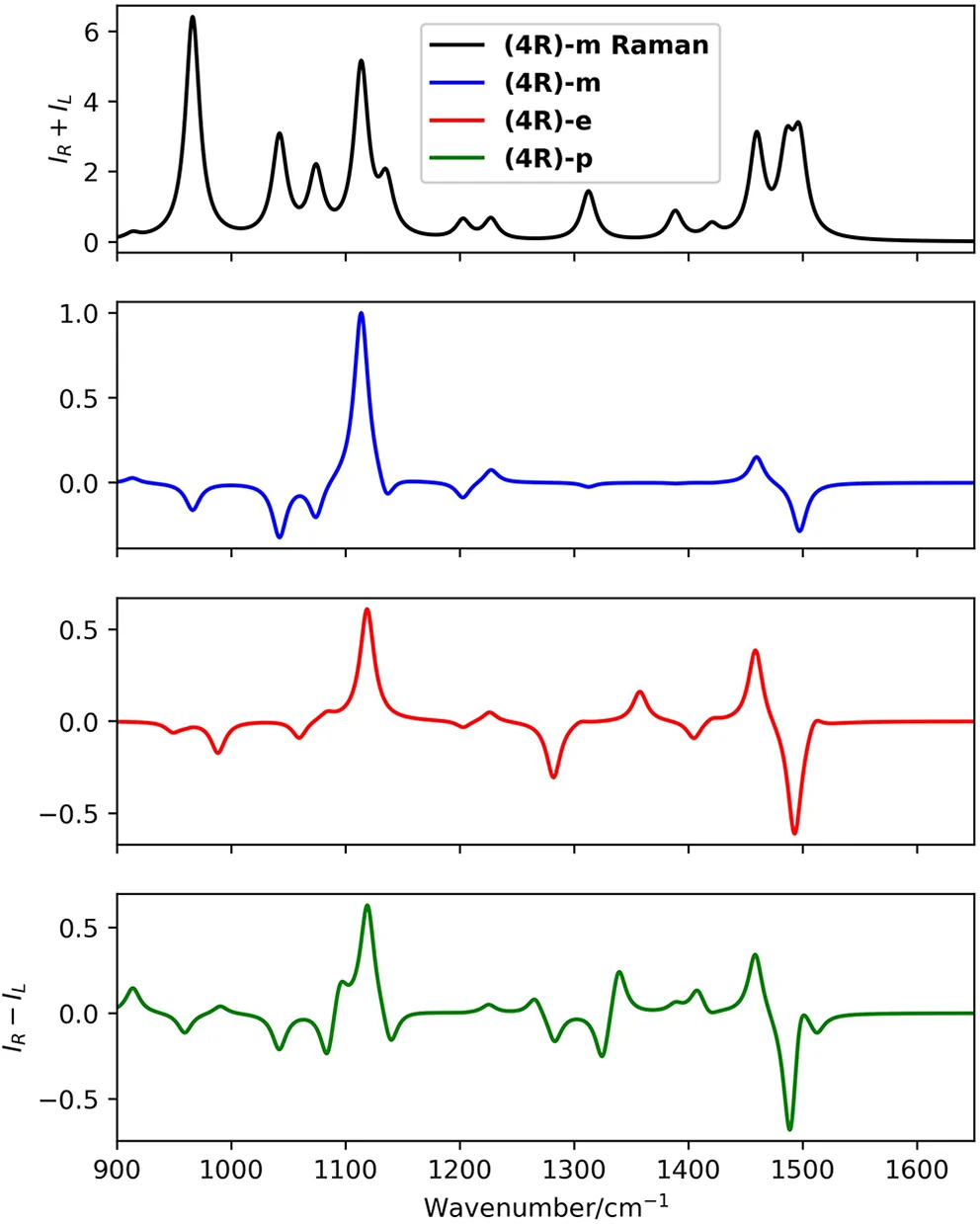
SCP­(180°) Raman (top) and VROA (lower three panels) spectra of **4*R*** in the mid-frequency range computed with CC2/asp-cc-pVDZ using the B3LYP/aug-cc-pVTZ geometry and Hessian. Arbitrary unit (unit increased by 10^3^ for Raman).

**Table 3:** Vibrational Modes with Largest SCP(180°) ROA Difference Parameters in the Mid-frequency Range for (4*R*)-X-2-Oxetanone Determined by CC2/asp-cc-pVDZ Using a B3LYP/aug-cc-pVTZ Geometry and Hessian

mode	ω/cm^–1^	*I*_R_ – *I*_L_ ^a^
**(4*R*)-m**		
ν_8_ C–H^c^ on **m**	1497.1	–2.5198
ν_10_ C–H^c^ on C3	1459.6	1.4046
ν_17_ ring breathing (esp. C2–O1), C–C btw C4 and **m**	1113.6	8.8944
ν_18_ C–H^b^ on C3, C–H^d^ on C4 and **m**	1074.0	–1.8900
ν_19_ C2–O1, C4–O1, C–H^d^ on **m**	1042.0	–2.7611
**(4*R*)-e**		
ν_12_ C–H^c^ on **e**	1492.8	–5.5422
ν_13_ C–H^c^ on C3	1458.6	3.5851
ν_18_ C–H^b^ on all	1281.8	–2.6784
ν_22_ ring breathing (esp. C2–O1), C–C btw C4 and **e**	1118.7	5.2942
ν_25_ ring breathing, C–C on **e**	988.2	–1.4735
**(4*R*)-p**		
ν_15_ C–H^c^ on **p**	1489.1	–7.1893
ν_16_ C–H^c^ on C3	1458.4	3.2508
ν_21_C–H^d^ on C4 and **p**	1324.8	–2.7322
ν_27_ ring breathing (esp. C2–O1), C–C btw C4 and **p**	1119.0	5.6447
ν_29_ C–H^b^ on C3 and C4, also on **p**	1084.2	–2.7718

a Units of 10^–3^ Å^4^ amu^–1^.

b Rocking.

c Scissoring.

d Wagging.

Although VROA is not typically carried out in the high-frequency range, the most prominent peaks in the C–H stretching region of **3*S*** and **4*R*** are presented in Tables 4 and SI2, respectively. With several closely spaced peaks of large intensity, VROA in this range appears unpromising with respect to a search for characteristic chiral signatures.

**Table 4:** Vibrational Modes with Largest SCP(180°) ROA Difference Parameters in the High-frequency Range for (3*S*)-X-2-Oxetanone Determined by CC2/asp-cc-pVDZ Using a B3LYP/aug-cc-pVTZ Geometry and Hessian

mode	ω/cm^–1^	*I*_R_ – *I*_L_ ^a^
**(3*S*)-m**		
ν_2_ C–H^b^ on **m**	3105.9	33.9574
ν_3_ C–H^b^ on **m** and C3	3098.0	–18.5053
ν_4_ C–H^c^ on C4 and C3	3072.2	–21.3081
ν_5_ C–H on C3, C–H^c^ on C4 and **m**	3067.3	5.0279
**(3*S*)-e**		
ν_1_ C–H^b^ on C4	3130.3	18.1685
ν_3_ C–H^b^ on **e**	3086.6	–22.5999
ν_4_ C–H^c^ on C4 and C3	3071.5	–12.9024
ν_5_ C–H on C3, C–H^b^ on **e**	3065.1	75.1757
ν_6_ C–H on C3, C–H^b^ on **e**	3053.8	–56.2451
**(3*S*)-p**		
ν_1_ C–H^b^ on C4	3129.7	19.3321
ν_3_ C–H^b^ on **p**	3083.5	25.1812
ν_4_ C–H^c^ on C4	3071.9	–25.9411
ν_5_ C–H^b^ on C3 and **p**	3065.7	–26.2617

a Units of 10^–3^ Å^4^ amu^–1^.

b Antisymmetric stretch.

c Symmetric stretch.

### (3,4)-Disubstituted β-Propiolactones

VROA spectra were computed with CC2 for (3,4)-disubstituted β-propiolactones for all unique stereoisomers incorporating methyl (**m**) or ethyl (**e**) substituents. The midfrequency backscattering spectra are shown in Figure 7, and information on the peaks of greatest intensity is provided in Table 5. Note that in Figure 7, the enantiomeric forms with a (3*S*) configuration are presented.

**Figure 7:**
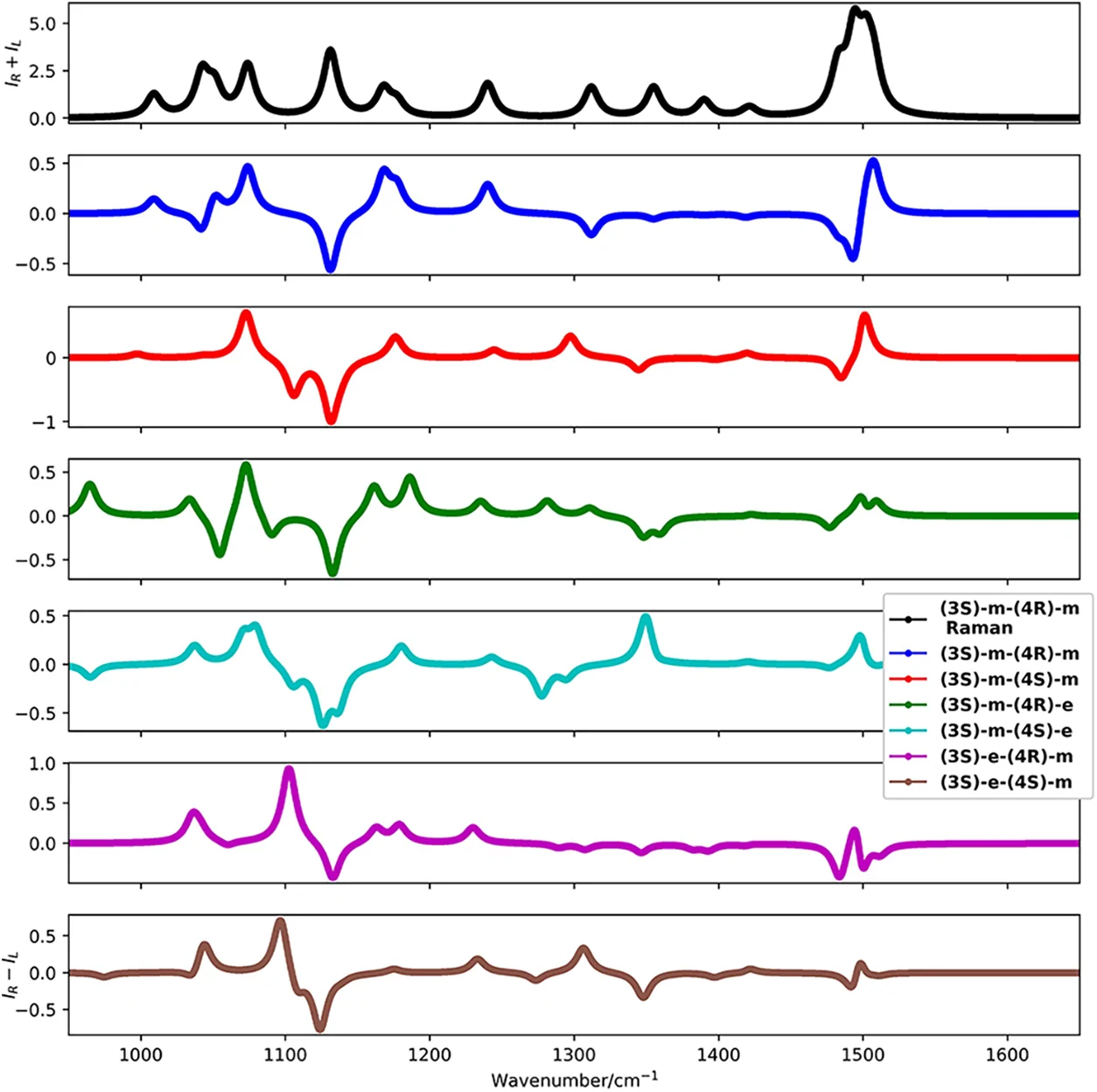
SCP­(180°) Raman (top) and VROA (lower 6 panels) spectra of 3,4-disubstituted β-propiolactones **3** and **4** in the mid-frequency range computed with CC2/asp-cc-pVDZ using the B3LYP/aug-cc-pVTZ geometry and Hessian. Arbitrary unit (unit increased by 10^3^ for Raman).

**Table 5:** Vibrational Modes with Largest SCP(180°) ROA Difference Parameters in the Mid-frequency Range for (3*S*)-X-(4*R*)-Y-2-Oxetanone Determined by CC2/asp-cc-pVDZ Using a B3LYP/aug-cc-pVTZ Geometry and Hessian

mode	ω/cm^–1^	*I*_R_ – *I*_L_ ^a^
**(3*S*)-m-(4*R*)-m**		
ν_10_ C–H^b^,^c^ on **m**’s	1507.4	3.6198
ν_12_ C–H^b^,^c^ on **m**’s	1493.6	–4.2283
ν_21_ ring breathing, C–C btw C3/C4 and **m**’s	1167.9	2.8527
ν_22_ ring breathing (esp. C2–O1), C–H^d^ on C3 and C4	1131.2	–4.3345
ν_23_ ring breathing, C–H^d^ on C3-**m**	1073.8	3.4883
**(3*S*)-m-(4*R*)-e**		
ν_14_ C–H^b^,^c^ on **m** and **e**	1500.4	–6.1529
ν_15_ C–H^b^,^c^ on **m** and **e**	1499.8	7.8074
ν_27_ ring breathing (esp. C2–O1), C–H^d^ on C3 and C4	1132.7	–5.0560
ν_29_ C–H^d^ on C3 and **m**, ring breathing	1072.5	4.9444
ν_30_ C–C btw C4 and **e**, C–C on **e**	1054.6	–3.8480
**(3*S*)-e-(4*R*)-m**		
ν_14_ C–H^c^,^b^ on **m** and **e**	1499.0	–7.3394
ν_15_ C–H^c^,^b^ on **m** and **e**	1495.5	6.4309
ν_16_ C–H^c^,^b^ on **m** and **e**	1483.9	–3.5762
ν_27_ ring breathing (esp. C2–O1), C–H^d^ on C3 and C4	1132.8	–3.5474
ν_28_ C–H^d^ on C3 and **e**, ring breathing	1102.4	7.0553
**(3*S*)-m-(4*S*)-m**		
ν_10_ C–H^c^,^b^ on **m**’s	1500.2	7.3734
ν_12_ C–H^c^,^b^ on **m**’s	1497.1	–3.7169
ν_22_ ring-breathing, C4-**m**, C3-**m**	1131.5	–6.9419
ν_23_ C3-**m**, C4-**m**, C–H^d^ on C3	1105.7	–4.2281
ν_24_ C–H^d^ on C3 and C3-**m**, ring breathing	1072.7	5.4674
**(3*S*)-m-(4*S*)-e**		
ν_13_ C–H^c^,^b^ on **m** and **e**	1500.5	–6.4161
ν_14_ C–H^c^,^b^ on **m** and **e**	1499.7	7.6709
ν_21_ C–H^d^ on C4 and on **e**	1349.6	3.9685
ν_26_ ring breathing (esp. C2–O1), C–H^d^ on C4, also on C3	1136.8	–2.8354
ν_27_ C3–C4, C3-**m**, C–H^d^ on C4	1125.6	–4.0350
**(3*S*)-e-(4*S*)-m**		
ν_14_ C–H^c^,^b^ on **m**	1497.5	4.4070
ν_15_ C–H^c^,^b^ on **e**	1493.4	–3.4356
ν_27_ C3–C4, C4-**m**, C–H^d^ on C3 and C4	1123.9	–5.7336
ν_29_ ring breathing, C3-**e**, C–H^d^ on C3 and C4	1096.6	6.0542
ν_30_ ring breathing (esp. C2–O1), C–H^d^ on C3 and C4	1043.5	3.3629

a Units of 10^–3^ Å^4^ amu^–1^.

b Rocking.

c Scissoring.

d Wagging.

Comparing first the disubstituted species with the same (3*S*,4*R*) stereochemistry, we observe a common, intense, negative intensity at ≈1130 cm^–1^. This vibrational mode corresponds to an asymmetrical wagging of the H atoms at the C3 and C4 positions, which reside on the same side of the β-propiolactone ring. The presence of the substituent at the C4 position eliminates the HCH scissoring mode at C4 previously present for the (3*S*) monosubstituted species, and instead this area of the spectrum becomes complicated by less transferable bending modes of varied intensity.

Both of the characteristic peaks (positive at 1115 cm^–1^, negative just under 1500 cm^–1^) identified for the (4*R*) monosubstituted species are now difficult to identify. The 1168 cm^–1^ peak for **(3*S*)-m-(4*R*)-m** roughly corresponds to the former, however it is now less intense than other peaks, and even more so for the **(4*R*)-e** and **(4*R*)-p** species. As in the case above, the latter peak near 1500 cm^–1^ becomes lost within a number of intense competitors.

Overall, the similarity of all of the spectra is notable, given the distinct orientations at the 4-position, and suggests that the configuration at the 3-position has a dominant effect. We attempt to quantify this conclusion in the following section.

The high-frequency range of the VROA spectra of (3,4)-disubstituted β-propiolactones **3** and **4** are shown in Figure 8, and the most prominent peaks in the C–H stretching regions are shown in Table SI3. VROA experiments are not typically carried out in this part of the spectrum.

**Figure 8:**
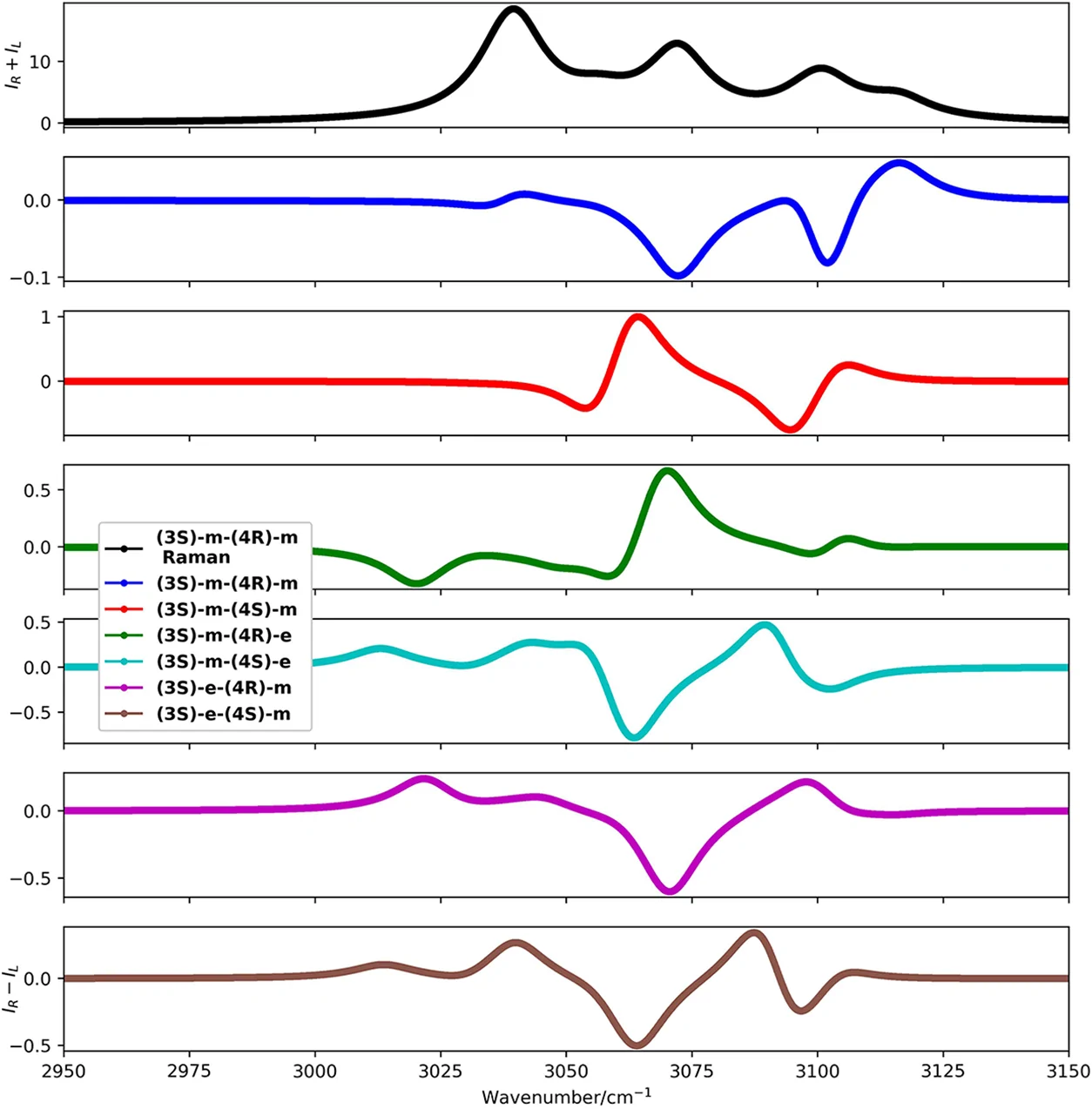
SCP­(180°) Raman (top) and VROA (lower 6 panels) spectra of 3,4-disubstituted β-propiolactones **3** and **4** in the high-frequency range computed with CC2/asp-cc-pVDZ using the B3LYP/aug-cc-pVTZ geometry and Hessian. Arbitrary unit (unit increased by 10^3^ for Raman).

VROA spectra were also computed for a variety of deuterated β-lactones. The resulting spectra are compared in Figures 9 and 10, with information on the peaks presented in Tables 6 and 7. While we do not report here the results from simultaneous alkyl- and isotopic- substitution, this may be a fruitful direction for future study.

**Figure 9:**
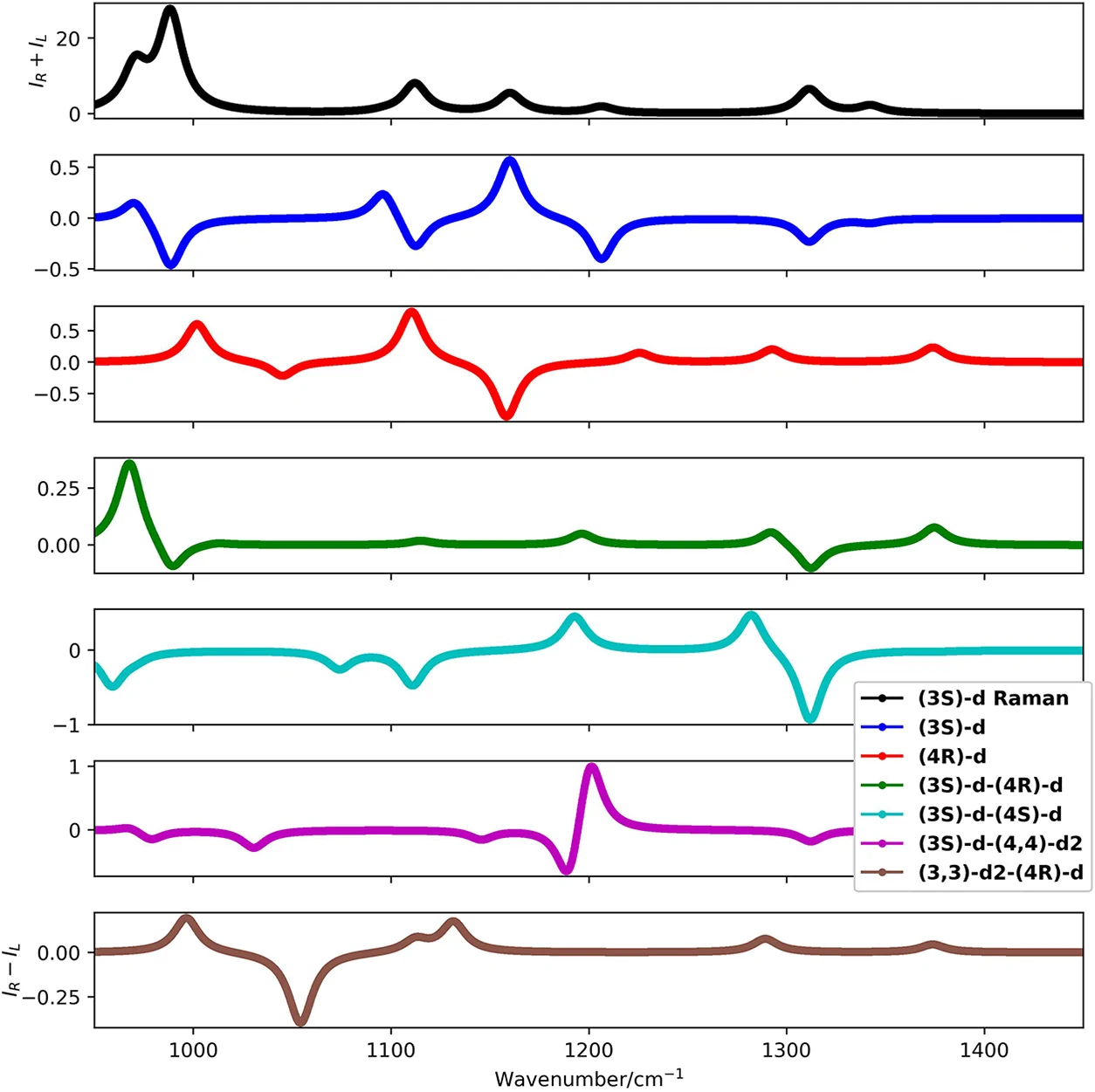
SCP­(180°) Raman (top) and VROA (lower 6 panels) spectra of deuterated β-propiolactone in the mid-frequency range computed with CC2/asp-cc-pVDZ using the B3LYP/aug-cc-pVTZ geometry and Hessian. No other substituents are present. Arbitrary unit (unit increased by 10^3^ for Raman).

**Figure 10:**
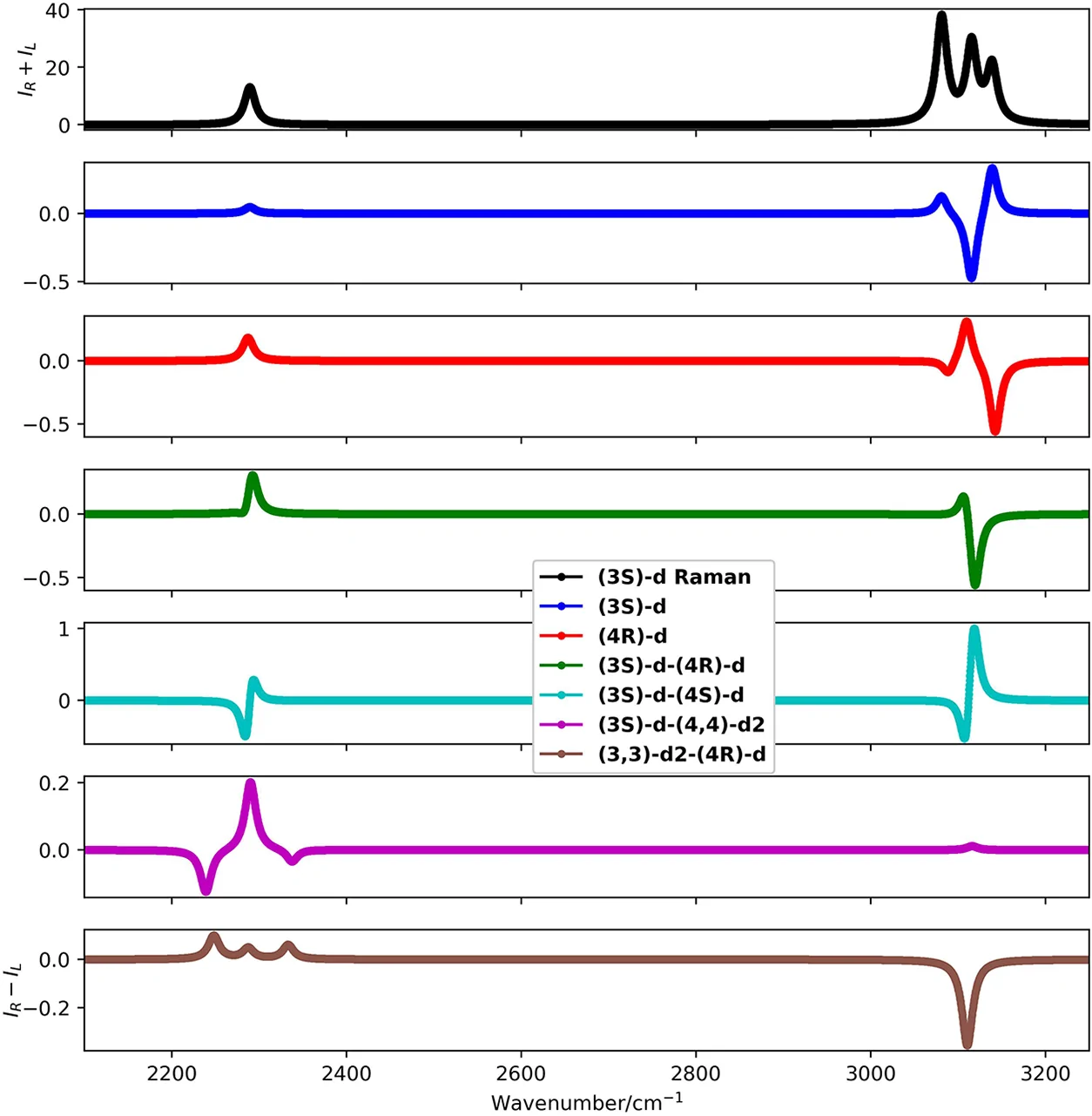
SCP­(180°) Raman (top) and VROA (lower 6 panels) spectra of deuterated β-propiolactone in the high-frequency range computed with CC2/asp-cc-pVDZ using the B3LYP/aug-cc-pVTZ geometry and Hessian. No other substituents are present. Arbitrary unit (unit increased by 10^3^ for Raman).

**Table 6:** Vibrational Modes with Largest SCP(180°) ROA Difference Parameters in the Mid-frequency Range for Deuterated 2-Oxetanone Determined by CC2/asp-cc-pVDZ Using a B3LYP/aug-cc-pVTZ Geometry and Hessian

mode	ω/cm^–1^	*I*_R_ – *I*_L_ ^a^
**(3*S*)-d-(4*S*)-d**		
**(3*S*)-d**		
ν_9_ C–H^b^ on C3, C–H^d^ on C4	1206.3	–1.2945
ν_10_ C–H^b^ on C4	1159.9	1.8409
ν_11_ ring deformation, C–H^d^ on C3 and C4	1111.9	–1.0539
ν_12_ C2–O1, C–H^d^ on C3 and C4	1096.2	0.9179
ν_13_ C3–C4	988.3	–1.5488
**(4*R*)-d**		
ν_7_ C–H^c^ on C4	1373.8	0.7330
ν_10_ C–H^b^ on C3, C2–O1	1158.2	–2.7754
ν_11_ C2–O1	1110.2	2.5803
ν_12_ ring breathing, C–H^b^ on C3,	1044.9	–0.7527
C–H^d^ on C4		
ν_13_ C3–C4, C3–C2, C–H^b^ on C4	1001.7	1.9087
**(3*S*)-d-(4*R*)-d**		
ν_6_ C–H^c^ on C4	1374.6	0.2455
ν_7_C–H^c^ on C3	1312.0	–0.3491
ν_8_ ring deformation, C–H^d^ on C4	1292.3	0.2162
ν_12_ C3–C4	988.9	–0.4177
ν_13_ ring breathing	967.6	1.1720
ν_7_ C–H^c^ on C3, C–H^d^ on C4	1311.7	–3.0254
ν_8_ C–H^d^ on C4, C–H^c^ on C3, C2–O	1282.1	1.6748
ν_9_ C–H^d^ on C3, C1–C3	1192.7	1.4398
ν_10_ C2–O, C–H^d^ on C4 and C3	1110.7	–1.4623
ν_13_ ring deformation, C-D^e^ on C4	958.9	–1.4568
**(3*S*)-d-(4,4)-d_2_**		
ν_6_ C–H/C-D^c^ on C3	1312.1	–0.5630
ν_7_ ring deformation, C–H^d^ on C3	1200.5	4.1806
ν_8_ ring deformation, C–H^d^ on C3	1189.8	–3.2865
ν_10_ ring breathing, C-D^d^ on C4	1030.5	–0.8619
ν_11_ ring deformation, C-D^e^ on C4 and C3	978.3	–0.5140
**(3,3)-d_2_-(4*R*)-d**		
ν_7_ ring deformation, C–H^d^ on C4	1289.0	0.2381
ν_8_ ring breathing, C-D^c^ on C3	1131.4	0.5292
ν_9_ ring deformation, C–H^d^	1112.3	0.2187
ν_10_ ring deformation, C-D^d^ on C3	1054.1	–1.2601
ν_11_ C-D^e^ on C3 and C4	996.3	0.6267

a Units of 10^–3^ Å^4^ amu^–1^.

b Rocking.

c Scissoring.

d Wagging.

e Twisting.

**Table 7:** Vibrational Modes with Largest SCP(180°) ROA Difference Parameters in the High-frequency Range for Deuterated 2-Oxetanone Determined by CC2/asp-cc-pVDZ Using a B3LYP/aug-cc-pVTZ Geometry and Hessian

mode	ω/cm^–1^	*I*_R_ – *I*_L_ ^a^
**(3*S*)-d**		
ν_1_ C–H^b^ on C4 and C3	3138.6	6.5103
ν_2_ C–H on C3, also on C4^b^	3115.2	–8.8888
ν_3_ C–H^c^ on C4	3081.0	2.4624
ν_4_ C-D	2289.1	0.8161
**(4*R*)-d**		
ν_1_ C–H^b^ on C3	3142.2	–9.9437
ν_2_ C–H on C4, also^b^ on C3	3109.5	6.1218
ν_3_ C–H^b^ on C3	3088.8	–2.0263
ν_4_ C-D	2287.0	3.1624
**(3*S*)-d-(4*R*)-d**		
ν_1_ C–H^c^ on C3 and C4	3118.8	–11.3273
ν_2_ C–H^b^ on C3 and C4	3107.7	5.5621
ν_3_ C-D^c^ on C3 and C4	2291.6	6.5252
ν_4_ C-D^b^ on C3 and C4	2284.6	–2.4275
**(3*S*)-d-(4*S*)-d**		
ν_1_C–H^b^ on C3 and C4	3117.2	25.4445
ν_2_ C–H^c^ on C3 and C4	3109.0	–19.3355
ν_3_ C-D^c^ on C3 and C4, C3–C4	2290.7	15.6502
ν_4_ C-D^b^ on C3 and C4	2285.8	–18.1819
**(3*S*)-d-(4,4)-d_2_**		
ν_1_ C–H on C3	3115.8	0.2034
ν_2_ C-D^b^ on C4	2337.4	–0.6642
ν_3_ C-D on C3	2289.8	3.5592
ν_4_ C-D^c^ on C4	2238.9	–2.2227
**(3,3)-d_2_-(4*R*)-d**		
ν_1_ C–H on C4	3110.4	–6.1578
ν_2_ C-D^b^ on C3	2333.0	1.0225
ν_3_ C-D on C4	2287.2	0.7909
ν_4_ C-D^c^ on C3	2247.9	1.6897

a Units of 10^–3^ Å^4^ amu^–1^.

b Antisymmetric stretch.

c Symmetric stretch.

### Similarity Metrics on β–Propiolactone Spectra

The similarity metrics described in the Methods section (eqs 8, 11, and 12–14) have been computed for the β-lactones in the frequency range 950–1650 cm^–1^ and are presented in Table 8. Since there is no obvious reference, we use the symmetrized forms of the metrics where applicable (eqs 12–14). For ease of comparison, the first two lines of the table are a comparison of **(3*S*)-m** with itself, and with its enantiomer, respectively. We have included results for the (3*S*) enantiomers and, for those structures only substituted at the 4-position, the (4*R*) enantiomers. These orientations are upward with respect to the β-lactone ring as represented in Figure 1.

**Table 8:** Similarity of VROA in the 950–1650 cm^–1^ Range for Substituted 2-Oxetanone Determined by CC2/asp-cc-pVDZ Using a B3LYP/aug-cc-pVTZ Geometry and Hessian^a^ ^,^ ^b^

Spectrum 1	Spectrum 2	SNO′	DNO	IDF′	RADF′	MJAC
**(3*S*)-m**	**(3*S*)-m**	1.0000	1.0000	0.0000	0.0000	1.0000
**(3*S*)-m**	**(3*R*)-m**	–1.0000	–1.0000	0.0000	2.0000	–1.0000
**(3*S*)-m**	**(3*S*)-e**	0.5235	0.5200	–0.0267	1.0237	0.3482
**(3*S*)-m**	**(3*S*)-p**	0.3740	0.3738	–0.0024	1.2678	0.2297
**(3*S*)-e**	**(3*S*)-p**	0.5771	0.5707	–0.0452	1.0685	0.3931
**(4*R*)-m**	**(4*R*)-m**	1.0000	1.0000	0.0000	0.0000	1.0000
**(4*R*)-m**	**(4*R*)-e**	0.5322	0.5298	–0.0184	1.1878	0.3581
**(4*R*)-m**	**(4*R*)-p**	0.4692	0.4686	–0.0049	1.2157	0.3055
**(4*R*)-e**	**(4*R*)-p**	0.6646	0.6576	–0.0425	1.0113	0.4823
**(3*S*)-m-(4*R*)-m**	**(3*S*)-m-(4*R*)-m**	1.0000	1.0000	0.0000	0.0000	1.0000
**(3*S*)-m-(4*R*)-m**	**(3*S*)-m-(4*R*)-e**	0.4675	0.4669	–0.0054	1.1245	0.3040
**(3*S*)-m-(4*R*)-m**	**(3*S*)-e-(4*R*)-m**	0.1563	0.1561	–0.0046	1.2648	0.0846
**(3*S*)-m-(4*R*)-e**	**(3*S*)-e-(4*R*)-m**	0.2499	0.2499	–0.0000	1.3030	0.1428
**(3*S*)-m-(4*S*)-m**	**(3*S*)-m-(4*S*)-e**	0.6035	0.5735	–0.2152	1.0498	0.3745
**(3*S*)-m-(4*S*)-m**	**(3*S*)-m-(4*S*)-m**	0.3434	0.3341	–0.1127	1.2880	0.1941
**(3*S*)-m-(4*S*)-e**	**(3*S*)-m-(4*S*)-m**	0.3020	0.3009	–0.0153	1.2693	0.1763
**(3*S*)-m-(4*R*)-m**	**(3*S*)-m-(4*S*)-m**	0.6802	0.6567	–0.1463	0.9605	0.4640
**(3*S*)-m-(4*R*)-m**	**(3*S*)-m-(4*S*)-e**	0.3512	0.3506	–0.0061	1.3072	0.2122
**(3*S*)-m-(4*R*)-m**	**(3*S*)-m-(4*S*)-m**	0.1975	0.1974	–0.0021	1.4652	0.1095
**(3*S*)-m-(4*R*)-e**	**(3*S*)-m-(4*S*)-e**	0.3429	0.3409	–0.0230	1.2814	0.2041
**(3*S*)-m-(4*R*)-e**	**(3*S*)-m-(4*S*)-m**	0.1540	0.1539	–0.0008	1.4534	0.0834
**(3*S*)-m-(4*R*)-m**	**(3*S*)-m-(4*S*)-m**	0.2671	0.2671	–0.0005	1.3062	0.1541
**(3*S*)-m-(4*S*)-m**	**(3*S*)-m-(4*R*)-m**	0.0020	0.0019	–0.0979	1.4188	0.0010
**(3*S*)-m-(4*S*)-m**	**(3*S*)-m-(4*R*)-e**	0.6278	0.6135	–0.0941	1.0471	0.4281
**(3*S*)-m-(4*S*)-e**	**(3*S*)-m-(4*R*)-m**	0.0962	0.0957	–0.0213	1.4218	0.0500

a Comparison of *I*_R_ – *I*_L_ for backscatter (180°) SCP in FFR approximation. Lineshapes assumed to be Lorentzian with a FWHM = 0.001 eV.

b See Methods section for metrics.

For the monosubstituted species, positive values of the SNO′, DNO, and MJAC in the first section of Table 8 uniformly indicate the same stereochemistry at the (3*S*) or at the (4*R*) position, respectively. As one would hope, and perhaps expect, the spectrum of **(3*S*)-e**, for example, is closer to that of **(3*S*)-m** than to that of **(3*R*)-m**. Thus, these metrics combined with the physical similarity of these molecular analogues is sufficient to identify the stereochemistry of a monosubstituted β-lactone—at least for the alkyl substituents investigated here.

Turning to the disubstituted β-lactones in the next block of data in the table, positive values of the SNO′, DNO, and MJAC likewise show the desired similarity for species of like stereochemistry. The smallest values of each were obtained in the comparison of **(3*S*)-m-(4*R*)-m** with **(3*S*)-e-(4*R*)-m**.

In the final block of data, disubstituted species of different stereochemistry are compared. Since we have chosen to tabulate results for the (3*S*) stereoisomers, the comparisons are being made between analogues with different configurations at the C4 position. It is readily seen that *all* of the SNO′, DNO, and MJAC values are positive. The magnitude is smallest for the comparison of **(3*S*)-m-(4*S*)-m** with **(3*S*)-e-(4*R*)-m**. Thus, the stereochemistry at C3, consistent for all the listed stereoisomers, is the dominant factor in spectral similarity as measured by these metrics.

Can the stereochemistry at the C4 position be distinguished? Unfortunately, the presented metric values show little pattern with regards to the configuration there. While not a perfect indicator, all four times the RADF′ is as large as 1.4, the stereochemistry at the 4-position of the compared structures differs. The results from these metrics comport with the visual inspection of the VROA spectra, as well as the specific vibrational mode analysis above.

In an attempt to find a diagnostic for the configuration at C4, various regions of the spectrum were evaluated for their effectiveness at distinguishing the stereochemistry. In the high-frequency C–H stretching region, the metrics completely fail with SNO′ and DNO overlaps being negative for half of the structures of like stereochemistry at both C3 and C4. A more narrow region of 950–1400 cm^–1^ was found to give similar results to those presented in Table 8. Regions were found in which the SNO′ and DNO values were larger overall, but their ability to distinguish different stereochemistry at the 4-position was not improved.

Given the starkly distinct prefactors appropriate to different types of VROA, it is possible that one could distinguish stereoisomers by carrying out a different type of VROA experiment. The various metric values (SNO′, DNO, etc.) were computed for the other types of VROA setups. The information provided in the SI may be used to do this for any of the studied molecules. Unfortunately, for these β-lactone analogues, the results were remarkably consistent for the distinct VROA forms despite the significant difference in tensor coefficients. In only one specific case, did any of the metrics produce a negative SNO′ or DNO value when comparing disubstituted species with the same configuration at the 3-position.

Evaluating the distinct similarity metrics in the current case, we note that the symmetrized version of SNO′ produces values that are virtually the same as DNO. Both of these have very high correlation with MJAC (for the values in Table 8 a correlation exceeding 0.98). The IDF′, while useful for comparing different computations of the same spectrum does not contribute to the stereochemical analysis. DNO produces values similar to but larger than MJAC.^56^ A potential role for the RADF′ value, which has a value of 2.0 for enantiomers was conjectured above, but since RADF′ has no upper-bound, a value above 1.0 is challenging to interpret. In conclusion, it seems reasonable in future work to use either DNO or MJAC, perhaps alongside RADF′ (or something similar) to track area of difference.

## Conclusion

VROA spectra of a number of alkyl-substituted β-lactones were computed with coupled cluster theory and analyzed. Spectra were compared using blackbox similarity metrics, by qualitative spectral comparison, and by analysis of the most intense peaks. VROA spectra of (3*S*)-monosubstituted β-lactones are characterized by a large negative peak associated with a coordinated ring-breathing motion coupled with a stretching motion of the C–C bond between the ring and the substituent at ≈1130 cm^–1^. A second positive peak at ≈1519 cm^–1^ corresponds with HCH scissoring on C4. VROA spectra of (4*R*)-monosubstituted β-lactones show a large positive signal at ≈1115 cm^–1^ corresponding with ring breathing and C–C stretching motion between the ring and the substituent. For disubstituted species of (3*S*,4*R*) stereochemistry, a mode involving ring-breathing motion and asymmetrical wagging of the H atoms on C3 and C4 (on the same side of the β-lactone ring) produces a large negative signal at ≈1130 cm^–1^. The spectra for (3*S*,4*S*) species are found to more resemble those of (3*S*,4*R*) than (3*R*,4*S*). Overall, the spectra are dominated by the stereochemistry at the 3-position. Thus, the ability to determine the stereochemistry of future β-lactones at this position via the use of VROA is promising. On the other hand, a distinctive signal for the stereochemistry at the 4-position in disubstituted species was not identified. Experimental verification of these findings would be beneficial in support of further theoretical developments as well as the characterization of new β-lactone derivatives.

## Supplementary Material



## Notes

Raman spectra for molecules in Figures 5–10; table of CC2 similarity metrics by basis set for HOOH; description of vibrational modes with largest difference parameters for (4*R*)-x and disubstituted β-lactones; vibrational frequencies, VROA invariants, and SCP(180°) *I*_R_ + *I*_L_ and *I*_R_ – *I*_L_ values for all molecules studied (PDF)
