# Supplementary material for: The Vibrational Raman Optical Activity of β‑Propiolactone Derivatives Computed with Coupled-Cluster Theory
Source: J Phys Chem A. 2026 Sep 4;130(37):7297–311. doi: 10.1021/acs.jpca.6c01773 (PMC13592081; doi:10.1021/acs.jpca.6c01773)
Supplement: Supplementary file 1 [file jp6c01773_si_001.pdf]

Supporting Information

The Vibrational Raman Optical Activity of β-propiolactone Derivatives Computed with Coupled-Cluster Theory

Mya S. Pederson,[‡] Luther D. Crum,[‡] Ethan J. Poncelet,[‡] Brendan M. Shumberger,[§] T. Daniel Crawford[§,1], and Rollin A. King[‡,2]

[‡]*Department of Chemistry, Bethel University, Saint Paul, Minnesota 55112, U.S.A.*
[§]*Department of Chemistry, Virginia Tech, Blacksburg, Virginia 24060, U.S.A.*

Contents

Raman spectra for molecules in Figures 5-10. **S2**

CC2 similarity metrics by basis set for HOOH. **S8**

Description of vibrational modes with largest VROA difference parameters for **(4R)-x** and di-substituted β-lactones studied. **S9**

Vibrational frequencies, VROA invariants, and SCP(180°) $I_R + I_L$ and $I_R - I_L$ values for all molecules studied. **S11**

[1]Electronic mail: crawdad@vt.edu
[2]Electronic mail: rking@bethel.edu

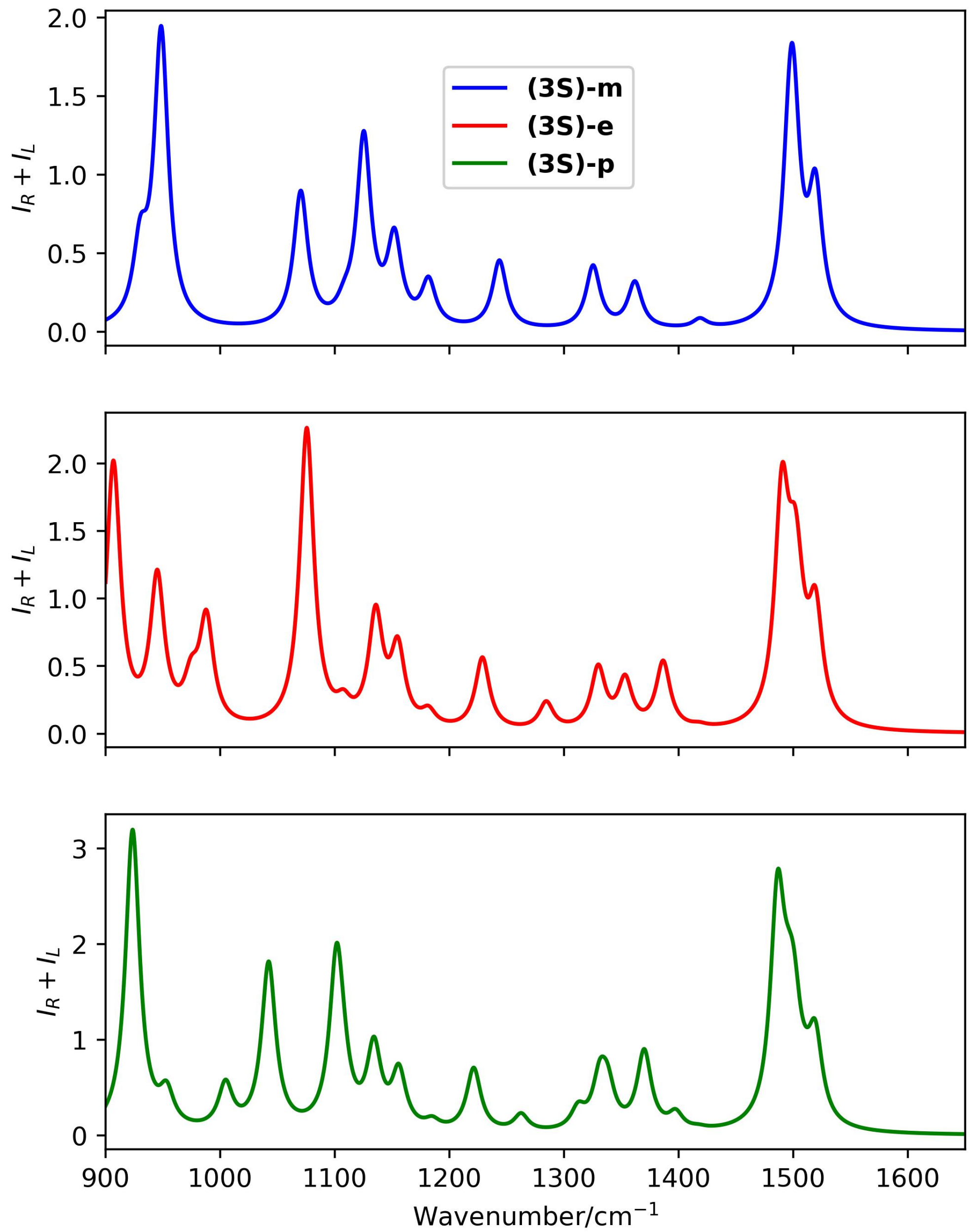

Figure SI1: The Raman spectra corresponding to Fig. 5 of text in units of Å^{4}amu^{-1}.

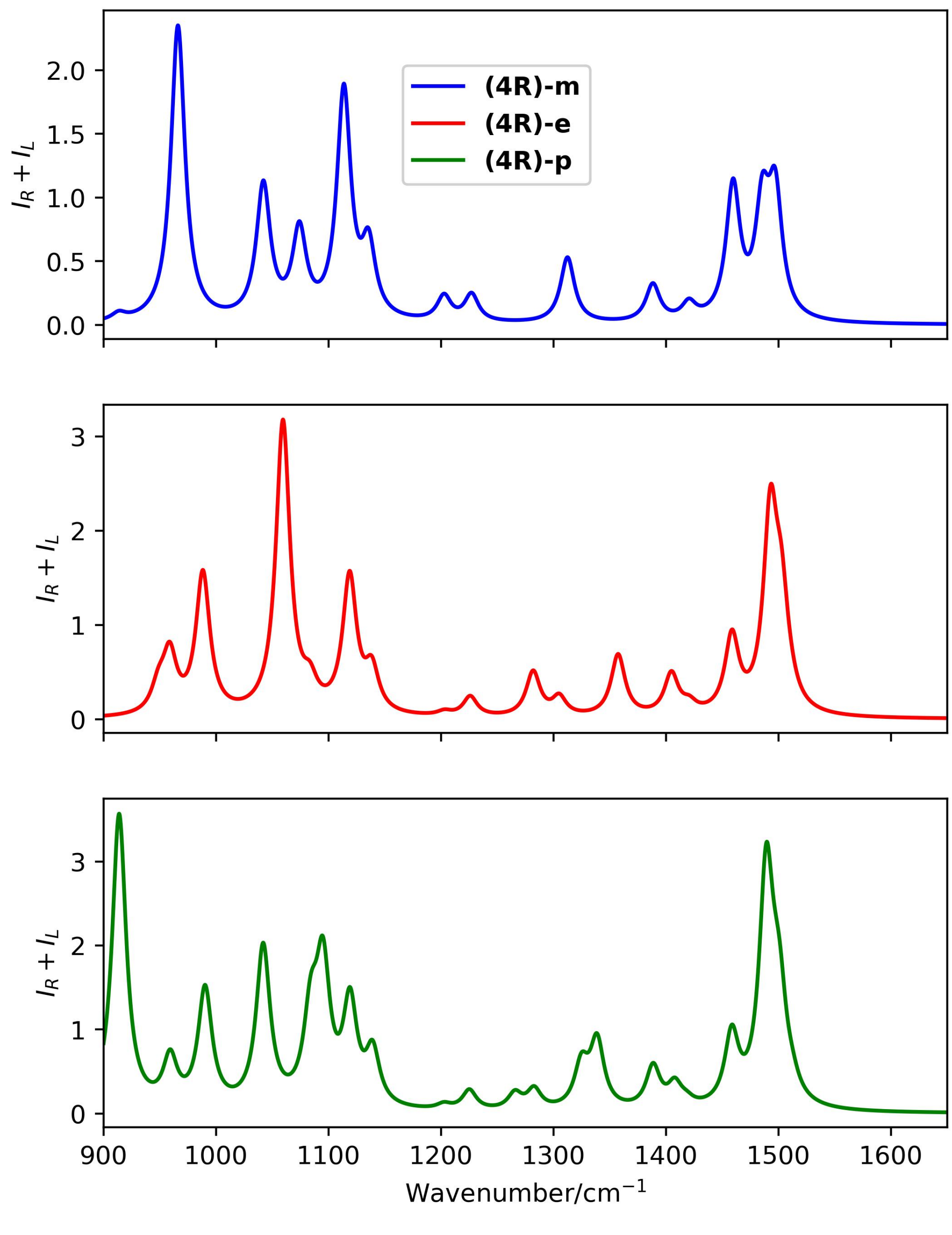

Figure SI2: The Raman spectra corresponding to Fig. 6 of text in units of $\text{Å}^4\text{amu}^{-1}$.

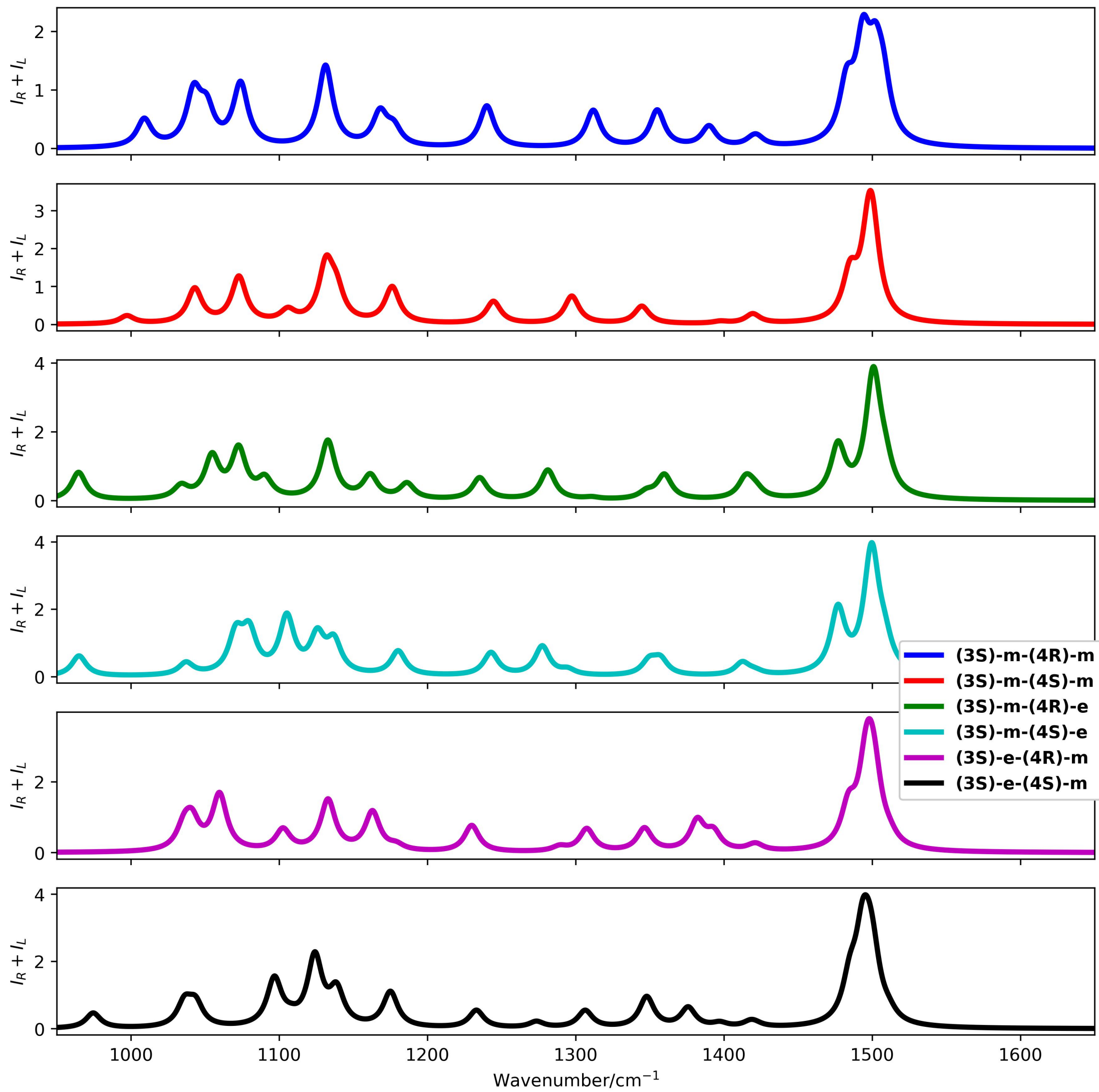

Figure SI3: The Raman spectra corresponding to Fig. 7 of text in units of Å^{4}amu^{-1}.

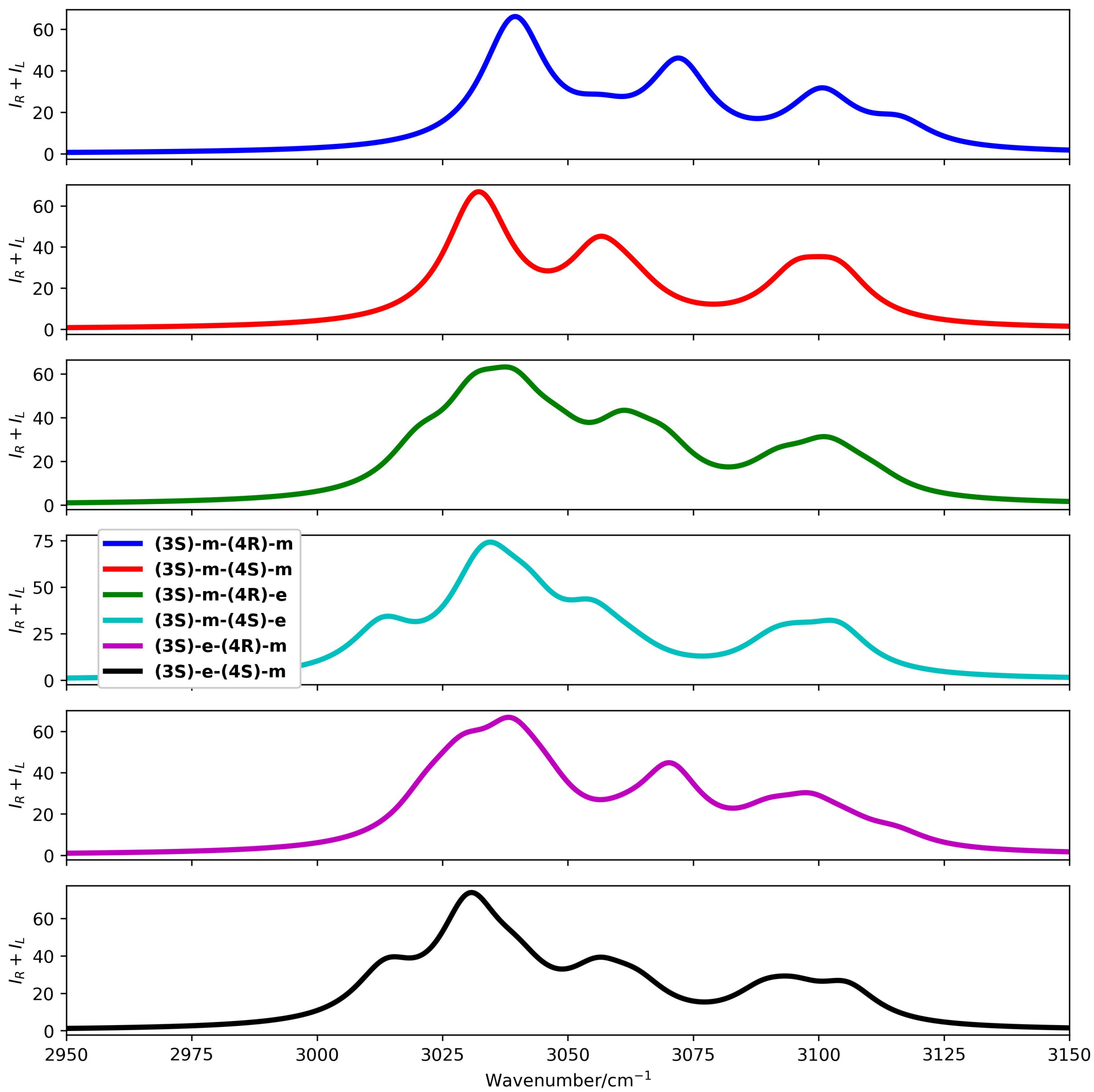

Figure SI4: The Raman spectra corresponding to Fig. 8 of text in units of Å^{4}amu^{-1}.

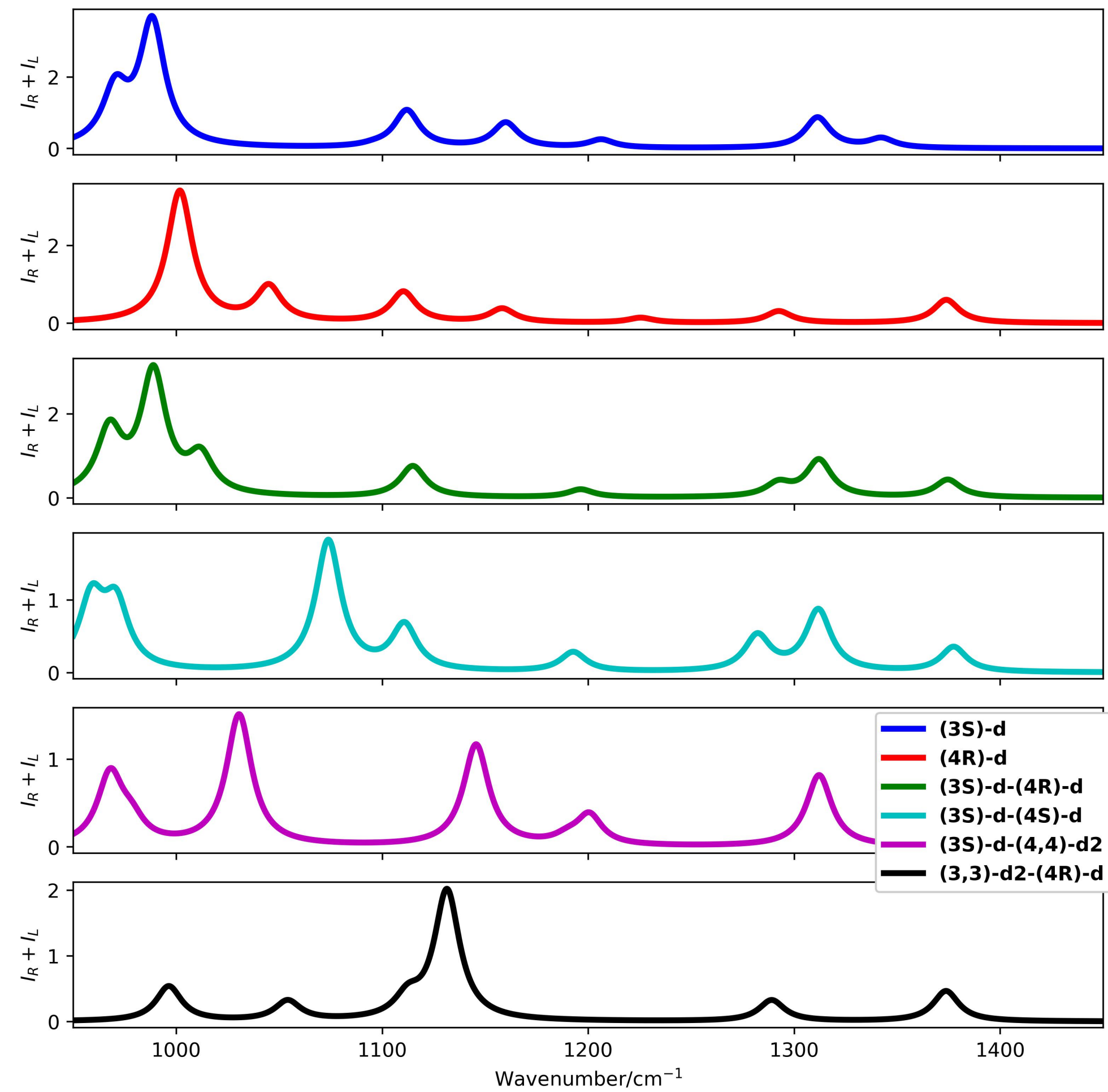

Figure SI5: The Raman spectra corresponding to Fig. 9 of text in units of $\mathring{A}^4$amu^{-1}.

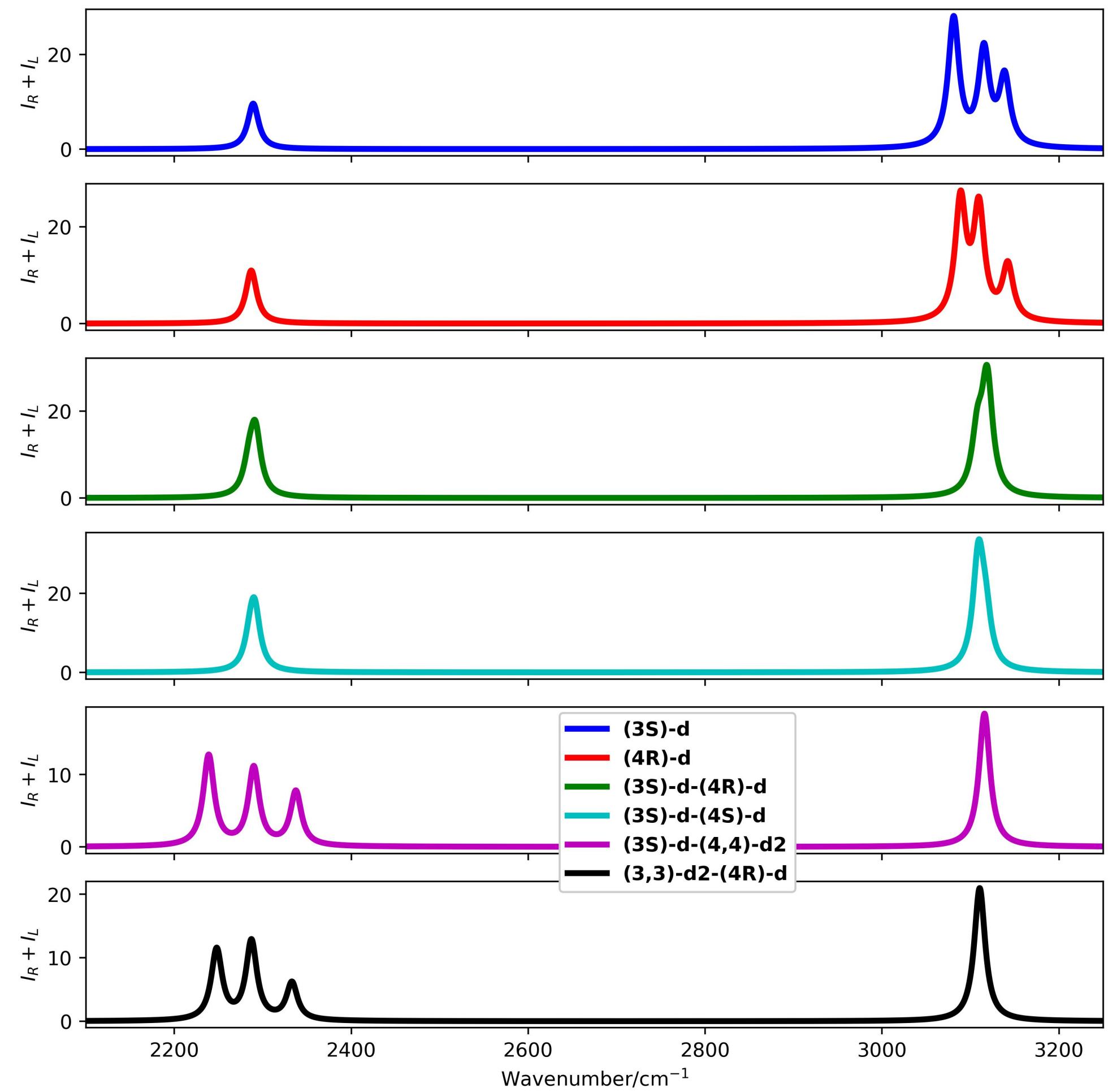

Figure SI6: The Raman spectra corresponding to Fig. 10 of text in units of $\text{Å}^4\text{amu}^{-1}$.

Table SI1: Similarity of VROA spectra for various one-electron basis sets computed with CC2 and a fixed geometry and Hessian (top) and a fully consistent geometry and Hessian (bottom).[a,b]

HOOH with CC2/aug-cc-pVQZ geometry and hessian						
Basis Set	NBF[c]	SNO	DNO	IDF	RADF	MJAC
cc-pVDZ	38	2.3266	0.9287	-5.2762	1.7261	0.4701
6-31+G*	42	1.6003	0.9866	-1.6313	0.7877	0.7880
augD-3-21G	48	0.9679	0.9719	0.0081	0.2202	0.9453
augT3-3-21G	48	0.9633	0.9773	0.0285	0.2435	0.9554
asp-cc-pVDZ	54	1.4080	0.9497	-1.1982	0.7428	0.7865
R-ORP	64	1.0885	0.9984	-0.1885	0.1296	0.9895
aug-cc-pVDZ	64	1.2745	0.9915	-0.6525	0.3759	0.9249
Sadlej-pVTZ	66	1.0865	0.9974	-0.1866	0.1320	0.9876
ORP	90	1.0672	0.9990	-0.1413	0.0682	0.9936
d-aug-cc-pVDZ	90	1.0432	0.9980	-0.0925	0.0876	0.9941
Sadlej-LPol-ds	98	1.0515	0.9983	-0.1094	0.0765	0.9939
Sadlej-LPol-dl	124	1.0050	0.9996	-0.0108	0.0324	0.9993
aug-cc-pVTZ	138	1.0903	0.9995	-0.1899	0.1098	0.9915
Sadlej-LPol-fs	146	0.9525	0.9997	0.0922	0.0631	0.9971
Sadlej-LPol-fl	196	0.9445	0.9998	0.1076	0.0630	0.9964
aug-cc-pVQZ	252	1.0000	1.0000	0.0000	0.0000	1.0000
HOOH with CC2						
Basis Set	NBF[c]	SNO	DNO	IDF	RADF	MJAC
cc-pVDZ	38	0.1148	0.0318	-12.0497	4.0761	0.0082
6-31+G*	42	0.0465	0.0228	-3.1689	2.7612	0.0091
augD-3-21G	48	-0.0118	-0.0077	-1.3805	2.6447	-0.0035
augT3-3-21G	48	0.0007	0.0007	0.0368	2.1821	0.0004
asp-cc-pVDZ	54	0.1129	0.0878	-0.6529	2.1773	0.0444
R-ORP	64	-0.0030	-0.0023	-0.8043	2.3061	-0.0011
aug-cc-pVDZ	64	0.0450	0.0317	-1.0179	2.1981	0.0151
Sadlej-pVTZ	66	0.0327	0.0388	0.2926	1.7159	0.0195
ORP	90	0.7042	0.7354	0.0829	0.8610	0.5806
d-aug-cc-pVDZ	90	0.0303	0.0314	0.0687	1.7579	0.0160
Sadlej-LPol-ds	98	0.0506	0.0466	-0.1791	1.7440	0.0238
Sadlej-LPol-dl	124	0.0231	0.0183	-0.5849	1.8738	0.0090
aug-cc-pVTZ	138	0.0976	0.0927	-0.1089	1.5026	0.0485
Sadlej-LPol-fs	146	0.0164	0.0105	-1.4271	2.1277	0.0048
Sadlej-LPol-fl	196	0.0392	0.0616	0.5960	1.4716	0.0287
aug-cc-pVQZ	252	1.0000	1.0000	0.0000	0.0000	1.0000

[a] Backscatter SCP(180°) in FFR approximation. Lineshapes assumed to be Lorentzian with a FWHM=0.001eV.
[b] See Methods section for definition of each metric. The aug-cc-pVTZ basis set was used as the reference.
[c] The number of basis functions.

Table SI2: Vibrational modes with largest SCP(180°) ROA difference parameters in the higher frequency range for (4R)-X-2-oxetanone determined by CC2/asp-cc-pVDZ using a B3LYP/aug-cc-pVTZ geometry and Hessian.

Mode	ω/cm^{-1}	$I_R - I_L$[u]
**(4R)-m**		
ν_1 C-H[a] on C3	3135.5	-7.7968
ν_2 C-H[a] on **m** and C4	3107.3	-57.8186
ν_3 C-H[s,a] on **m**, C-H on C4	3097.8	51.1113
ν_4 C-H[s] on C3	3081.5	14.0670
**(4R)-e**		
ν_1 C-H[a] on C3	3135.1	-22.3120
ν_2 C-H[s,a] on **e**	3098.8	-16.3065
ν_3 C-H[a] on **e**, C-H on C4	3088.2	44.5037
ν_5 C-H on C4, also C-H[a] on **e**	3069.7	-98.7403
ν_6 C-H[a] on **e**, C-H on C4	3053.5	60.4959
**(4R)-p**		
ν_1 C-H[a] on C3	3134.5	-24.3474
ν_2 C-H[a] on **p**	3090.4	14.5139
ν_3 C-H[a] on **p**	3084.6	-46.9376
ν_4 C-H[s] on C3	3080.4	19.7867
ν_5 C-H[a] on **p** and C4	3072.4	29.4619

[u] Units of 10^{-3}Å^{4}amu^{-1}.
[a] Antisymmetric stretch.
[s] Symmetric stretch.

Table SI3: Vibrational modes with largest SCP(180°) ROA difference parameters in the higher frequency range for (3S)-X-(4R/S)-Y-2-oxetanone determined by CC2/asp-cc-pVDZ using a B3LYP/aug-cc-pVTZ geometry and Hessian.

Mode	ω/cm^{-1}	$I_R - I_L$ [u]
**(3S)-m-(4R)-m**		
ν_1 C-H^{s,a} on **m**'s, C-H^{s} on C3 and C4	3116.3	4.4338
ν_2 C-H^{a} on **m**'s, C-H^{a} on C3 and C4	3108.0	4.3623
ν_3 C-H^{s,a} on C3-**m**, also C-H^{a} on C4-**m**	3101.0	-20.1027
ν_4 C-H^{s,a} on C4-**m**, also C-H^{s,a} on C3-**m**	3097.9	12.7515
ν_5 C-H^{s} on C3 and C4	3072.2	-8.4432
**(3S)-m-(4R)-e**		
ν_2 C-H^{a} on e, also on **m**	3104.3	26.4496
ν_3 C-H^{a} on **m**	3100.8	-25.0564
ν_5 C-H^{s} on C3 and C4, also on **m** and **e**	3069.1	78.2926
ν_6 C-H on C3, C-H^{a} on **e**	3060.9	-47.3639
ν_{10} C-H^{s} on first C of **e**	3020.2	-27.8465
**(3S)-e-(4R)-m**		
ν_2 C-H^{s,a} on **e**	3105.7	-8.2959
ν_3 C-H^{s,a} on **m**	3098.3	24.0361
ν_5 C-H^{s} on C3 and C4, also C-H^{a} **m** and **e**	3070.4	-55.9759
ν_7 C-H^{a} on C3, C4, and first C of **e**	3045.3	8.7202
ν_{10} C-H^{s} on first C of **e**	3021.8	21.6557
**(3S)-m-(4S)-m**		
ν_2 C-H^{s,a} on C3-**m**, C-H^{a} on C4-**m**	3103.5	45.1683
ν_3 C-H^{s,a} on **m**'s	3095.5	-54.3332
ν_4 C-H^{s,a} on **m**'s	3095.0	-34.6249
ν_5 C-H^{a} on C4 and C3	3063.0	136.2357
ν_6 C-H^{s} on C3 and C4	3056.2	-96.8505
**(3S)-m-(4S)-e**		
ν_1 C-H^{s,a} on **m** and **e**	3104.2	42.7241
ν_2 C-H^{s,a} on **e** and **m**	3103.7	-61.4240
ν_4 C-H^{s,a} on **e**	3090.7	72.4773
ν_5 C-H^{a} on C3, C4, and **e**	3062.6	-93.9203
ν_6 C-H^{s} on C3 and C4, C-H^{a} on **e**	3054.9	50.1524
**(3S)-e-(4S)-m**		
ν_2 C-H^{a} on **m** and C4	3105.4	32.1301
ν_3 C-H^{s,a} on **m** and C4	3095.1	-55.6149
ν_4 C-H^{s,a} on **e**	3088.9	59.5935
ν_5 C-H^{s,a} on C4, C3, and **m**	3063.7	-50.3483
ν_7 C-H^{a} on C3 and **e**	3039.8	27.1965

[u] Units of 10^{-3}Å^{4}amu^{-1}.
[a] Antisymmetric stretch.
[s] Symmetric stretch.

Table SI4: VROA results for (3S)-methyl-2-oxetanone determined by CC2/asp-cc-pVDZ using a B3LYP/aug-cc-pVTZ geometry and Hessian.

		VROA Invariants[a]			SCP (180°)[b]	
Mode	ω/cm^{-1}	$\alpha G'$	$\beta(G')^2$	$\beta(A)^2$	$I_R + I_L$	$I_R - I_L$
ν_1	3131.2	1.892e-06	5.179e-06	-2.100e-05	357.0567	-0.1823
ν_2	3105.9	1.370e-06	5.789e-05	8.436e-04	230.4818	33.9574
ν_3	3098.0	-1.251e-06	-2.863e-05	-4.685e-04	230.8825	-18.5053
ν_4	3072.2	2.882e-06	-6.043e-05	-4.571e-04	814.5905	-21.3081
ν_5	3067.3	1.576e-06	2.854e-05	6.501e-05	341.8561	5.0279
ν_6	3034.5	-1.881e-06	-2.146e-06	4.813e-06	740.9610	-0.0542
ν_7	1898.6	-4.051e-07	3.659e-06	3.187e-05	158.2046	1.4301
ν_8	1519.4	9.994e-09	3.868e-06	6.755e-05	19.2345	2.6421
ν_9	1499.9	2.419e-08	4.116e-06	1.086e-05	25.9880	0.7749
ν_{10}	1496.8	-1.203e-09	-3.733e-06	-4.162e-05	16.6057	-1.7629
ν_{11}	1418.6	-3.277e-09	-6.571e-07	1.902e-06	1.3251	-0.0023
ν_{12}	1361.8	-1.929e-09	-3.545e-06	-5.067e-05	6.8859	-2.0463
ν_{13}	1325.4	-8.094e-08	-6.929e-07	3.489e-05	9.3763	1.0952
ν_{14}	1243.6	1.505e-07	-5.093e-06	8.083e-06	10.1807	-0.2402
ν_{15}	1181.8	1.474e-07	7.630e-06	6.129e-05	6.6452	2.8098
ν_{16}	1152.0	2.104e-07	-3.063e-06	-3.849e-05	12.7315	-1.5915
ν_{17}	1125.3	1.666e-07	-7.787e-06	-1.142e-04	28.2049	-4.5904
ν_{18}	1108.2	-6.166e-08	-1.330e-07	-8.256e-07	1.9752	-0.0409
ν_{19}	1070.3	8.984e-08	4.198e-06	1.228e-04	20.1857	4.5180
ν_{20}	948.5	8.744e-08	3.324e-06	5.075e-05	44.1105	2.0269
ν_{21}	930.3	-3.856e-07	-5.994e-06	-3.409e-05	10.8649	-1.7380
ν_{22}	876.5	-2.148e-07	2.823e-06	2.830e-05	34.0284	1.2273
ν_{23}	816.3	-3.994e-07	3.445e-06	-1.837e-05	82.1145	-0.2680
ν_{24}	735.4	2.693e-07	-2.511e-06	3.335e-05	29.2616	0.8617
ν_{25}	689.4	-5.260e-08	1.040e-05	-6.007e-07	12.1611	1.0209
ν_{26}	504.9	8.607e-08	-7.253e-06	-2.869e-05	12.8777	-1.6841
ν_{27}	360.5	-9.500e-08	1.078e-06	8.647e-06	4.9744	0.3966
ν_{28}	219.3	-2.109e-08	3.759e-07	-3.077e-06	7.8241	-0.0651
ν_{29}	211.6	-2.241e-09	-1.155e-07	-1.616e-06	2.6105	-0.0655
ν_{30}	146.8	6.627e-09	-1.976e-06	-1.392e-05	1.6621	-0.6626

[a] Atomic units.
[b] Units of Å^4amu^{-1} for Raman, 10^{-3}Å^4amu^{-1} for VROA.

Table SI5: VROA results for (3S)-methyl-2-oxetanone (3000+) determined by CCSD/asp-cc-pVDZ using a B3LYP/aug-cc-pVTZ geometry and Hessian.

		VROA Invariants[a]			SCP (180°)[b]	
Mode	ω/cm^{-1}	$\alpha G'$	$\beta(G')^2$	$\beta(A)^2$	$I_R + I_L$	$I_R - I_L$
ν_1	3131.2	1.656e-06	2.997e-06	-2.284e-05	294.5984	-0.4621
ν_2	3105.9	1.267e-06	5.681e-05	6.950e-04	199.7442	28.8859
ν_3	3098.0	-1.017e-06	-2.702e-05	-3.692e-04	200.8642	-15.0306
ν_4	3072.2	2.738e-06	-5.890e-05	-4.210e-04	695.1640	-19.9507
ν_5	3067.3	1.460e-06	3.475e-05	6.826e-05	288.9701	5.7577
ν_6	3034.5	-1.806e-06	-1.479e-06	8.212e-06	643.9887	0.1260

[a] Atomic units.
[b] Units of $\mathrm{\AA}^4$amu^{-1} for Raman, $10^{-3}\mathrm{\AA}^4$amu^{-1} for VROA.

Table SI6: VROA results for (3S)-methyl-2-oxetanone (950-1900) determined by CCSD/asp-cc-pVDZ using a B3LYP/aug-cc-pVTZ geometry and Hessian.

		VROA Invariants[a]			SCP (180°)[b]	
Mode	ω/cm^{-1}	$\alpha G'$	$\beta(G')^2$	$\beta(A)^2$	$I_R + I_L$	$I_R - I_L$
ν_1	1898.6	-4.851e-07	2.157e-06	3.183e-05	102.2632	1.2785
ν_2	1519.4	2.791e-08	4.709e-06	6.663e-05	17.8141	2.6956
ν_3	1499.9	2.187e-08	3.188e-06	9.026e-06	22.9837	0.6205
ν_4	1496.8	8.128e-10	-4.295e-06	-4.260e-05	15.1865	-1.8522
ν_5	1418.6	-6.378e-10	-2.341e-07	1.294e-06	1.2192	0.0198
ν_6	1361.8	-2.166e-09	-3.926e-06	-3.681e-05	6.0895	-1.6220
ν_7	1325.4	-1.098e-08	-1.908e-07	2.128e-05	4.7580	0.6913
ν_8	1243.6	8.929e-08	-3.710e-06	4.186e-06	7.5481	-0.2318
ν_9	1181.8	4.364e-08	6.897e-06	5.148e-05	5.4713	2.4091
ν_{10}	1152.0	1.951e-07	-3.629e-06	-3.311e-05	11.6465	-1.4687
ν_{11}	1125.3	1.153e-07	-5.469e-06	-7.043e-05	18.6921	-2.8986
ν_{12}	1108.2	8.635e-10	-4.877e-07	-2.255e-06	2.7540	-0.1241
ν_{13}	1070.3	-4.986e-08	3.991e-06	7.348e-05	13.3472	2.8524
ν_{14}	948.5	-6.420e-08	3.446e-06	4.088e-05	30.0939	1.7097

[a] Atomic units.
[b] Units of Å^{4}amu^{-1} for Raman, 10^{-3}Å^{4}amu^{-1} for VROA.

Table SI7: VROA results for (3S)-methyl-2-oxetanone determined by CC2/asp-cc-pVDZ using a CC2/asp-cc-pVDZ geometry and Hessian.

		VROA Invariants[a]			SCP (180°)[b]	
Mode	ω/cm^{-1}	$\alpha G'$	$\beta(G')^2$	$\beta(A)^2$	$I_R + I_L$	$I_R - I_L$
ν_1	3189.5	1.831e-06	-2.907e-07	-1.198e-04	377.8752	-4.0281
ν_2	3170.8	4.355e-07	1.754e-05	5.168e-04	262.0013	19.0055
ν_3	3162.6	-8.980e-07	8.781e-06	2.516e-05	228.3477	1.7191
ν_4	3118.0	4.682e-06	-6.420e-05	-5.456e-04	613.9411	-24.6396
ν_5	3105.5	1.121e-06	3.793e-05	5.888e-05	578.7531	5.7641
ν_6	3060.1	-1.646e-06	2.548e-07	6.013e-07	772.0825	0.0456
ν_7	1809.6	-4.405e-07	3.260e-06	5.017e-05	175.7311	2.0012
ν_8	1497.8	5.931e-09	8.146e-06	1.249e-04	18.2838	4.9840
ν_9	1487.2	2.049e-08	6.229e-06	-1.405e-07	30.3351	0.6191
ν_{10}	1482.6	-1.688e-09	-8.946e-06	-8.313e-05	16.4781	-3.6706
ν_{11}	1395.9	-1.987e-09	-1.486e-06	1.034e-07	0.8938	-0.1453
ν_{12}	1353.9	-1.113e-08	-3.137e-06	-4.236e-05	11.1816	-1.7282
ν_{13}	1289.6	-7.159e-08	-3.416e-07	3.156e-05	9.4938	1.0193
ν_{14}	1226.0	1.202e-07	-4.656e-06	2.908e-05	10.6961	0.5044
ν_{15}	1181.1	1.573e-07	8.301e-06	7.662e-05	7.0235	3.3888
ν_{16}	1134.1	6.044e-08	-1.515e-05	-1.612e-04	28.0642	-6.8965
ν_{17}	1128.4	4.435e-07	3.426e-06	7.007e-06	17.3761	0.5770
ν_{18}	1084.5	-8.928e-09	6.813e-07	-5.911e-05	3.0057	-1.9047
ν_{19}	1038.5	3.266e-09	3.389e-06	1.539e-04	27.3477	5.4755
ν_{20}	929.6	1.713e-07	6.672e-06	2.588e-05	28.8621	1.5321
ν_{21}	909.9	-1.023e-06	-8.634e-06	-9.259e-06	29.0574	-1.1736
ν_{22}	871.3	2.800e-07	-1.031e-06	-1.408e-06	43.5868	-0.1503
ν_{23}	812.2	-5.866e-07	5.991e-06	-3.218e-06	80.0731	0.4925
ν_{24}	712.5	3.160e-07	-1.594e-06	1.404e-05	44.2123	0.3088
ν_{25}	672.6	7.191e-08	1.038e-05	1.536e-05	10.5134	1.5524
ν_{26}	489.2	6.452e-08	-7.796e-06	-2.834e-05	12.9841	-1.7266
ν_{27}	357.9	-9.312e-08	1.325e-06	8.269e-06	4.6732	0.4087
ν_{28}	230.9	-5.820e-09	-7.198e-07	2.105e-07	1.3632	-0.0650
ν_{29}	206.5	-2.499e-08	8.375e-07	-6.431e-06	9.1096	-0.1308
ν_{30}	125.4	5.661e-09	-2.120e-06	-1.358e-05	1.9783	-0.6655

[a] Atomic units.
[b] Units of Å^{4}amu^{-1} for Raman, 10^{-3}Å^{4}amu^{-1} for VROA.

Table SI8: VROA results for (3S)-ethyl-2-oxetanone determined by CC2/asp-cc-pVDZ using a B3LYP/aug-cc-pVTZ geometry and Hessian.

		VROA Invariants[a]			SCP (180°)[b]	
Mode	ω/cm^{-1}	$\alpha G'$	$\beta(G')^2$	$\beta(A)^2$	$I_R + I_L$	$I_R - I_L$
ν_1	3130.3	2.793e-06	3.841e-05	4.291e-04	328.2598	18.1685
ν_2	3094.4	6.944e-07	2.749e-05	2.141e-05	415.5473	3.4672
ν_3	3086.6	2.047e-07	-4.755e-05	-5.344e-04	82.8572	-22.5999
ν_4	3071.5	1.186e-06	-3.159e-05	-2.918e-04	716.0107	-12.9024
ν_5	3065.1	3.618e-06	1.301e-04	1.862e-03	153.8164	75.1757
ν_6	3053.8	-4.885e-08	-7.226e-05	-1.468e-03	444.2585	-56.2451
ν_7	3026.5	-1.761e-06	-1.178e-05	-1.397e-04	186.1694	-5.8435
ν_8	3023.1	2.367e-06	2.560e-05	-2.799e-05	1122.6607	1.6288
ν_9	1896.5	-5.238e-07	7.247e-06	4.509e-06	156.1019	0.8762
ν_{10}	1519.4	-2.275e-09	3.454e-06	8.376e-05	18.7918	3.1417
ν_{11}	1505.9	-2.839e-09	2.756e-06	7.982e-05	4.6160	2.9403
ν_{12}	1501.5	3.459e-09	3.240e-06	-3.803e-05	21.8010	-0.9450
ν_{13}	1490.1	1.996e-08	-8.575e-06	-6.102e-05	38.2176	-2.8954
ν_{14}	1418.0	-2.245e-09	-1.218e-07	-2.049e-06	0.4406	-0.0806
ν_{15}	1386.5	-1.879e-08	1.271e-06	-6.400e-06	11.6485	-0.0863
ν_{16}	1353.3	4.230e-08	-2.621e-06	-2.831e-05	8.3290	-1.2074
ν_{17}	1329.8	-1.052e-07	9.732e-06	-2.655e-05	10.6037	0.0884
ν_{18}	1284.5	1.203e-09	-4.679e-06	3.098e-05	4.7036	0.5654
ν_{19}	1228.8	4.806e-07	-4.906e-06	5.778e-05	12.5469	1.4373
ν_{20}	1181.7	7.593e-10	8.464e-06	5.439e-05	2.5920	2.6629
ν_{21}	1154.9	2.042e-08	-6.166e-06	-4.886e-05	13.3370	-2.2482
ν_{22}	1135.7	1.125e-07	-5.113e-06	-6.793e-05	19.3512	-2.7796
ν_{23}	1107.6	-1.642e-07	-3.191e-07	2.047e-05	3.0853	0.6515
ν_{24}	1075.6	1.157e-07	-4.118e-06	3.732e-05	52.3178	0.8333
ν_{25}	988.0	2.972e-07	7.193e-06	1.258e-05	18.3596	1.1401
ν_{26}	974.6	8.276e-08	1.220e-06	5.814e-06	6.6400	0.3163
ν_{27}	945.1	-5.569e-07	-3.562e-06	1.480e-05	25.6886	0.1374
ν_{28}	906.8	4.445e-07	3.500e-06	6.293e-07	46.2792	0.3715
ν_{29}	840.5	-1.309e-06	3.206e-06	8.432e-06	54.6900	0.6025
ν_{30}	793.8	-8.090e-08	3.342e-06	2.273e-05	18.8381	1.0935
ν_{31}	729.1	3.146e-07	-7.327e-06	1.273e-05	27.5065	-0.3086
ν_{32}	669.3	-3.207e-07	4.152e-06	-1.179e-05	19.9403	0.0223
ν_{33}	542.2	1.511e-08	-1.006e-05	-4.297e-05	6.9929	-2.4414
ν_{34}	378.5	4.622e-09	4.565e-06	7.424e-06	6.1469	0.7049
ν_{35}	308.6	-3.517e-08	-1.219e-06	5.464e-06	12.1981	0.0603
ν_{36}	230.6	-1.488e-08	2.521e-07	-2.717e-06	0.3843	-0.0654
ν_{37}	158.6	-3.697e-09	1.920e-06	8.789e-06	5.9843	0.4856
ν_{38}	141.3	2.934e-09	-1.292e-06	-1.014e-05	1.2431	-0.4678
ν_{39}	96.5	8.532e-09	-3.741e-06	-2.520e-05	1.9279	-1.2158

[a] Atomic units.
[b] Units of Å^{4}amu^{-1} for Raman, 10^{-3}Å^{4}amu^{-1} for VROA.

Table SI9: VROA results for (3S)-ethyl-2-oxetanone (3000+) determined by CCSD/asp-cc-pVDZ using a B3LYP/aug-cc-pVTZ geometry and Hessian.

		VROA Invariants[a]			SCP (180°)[b]	
Mode	ω/cm^{-1}	$\alpha G'$	$\beta(G')^2$	$\beta(A)^2$	$I_R + I_L$	$I_R - I_L$
ν_1	3130.3	2.395e-06	3.700e-05	3.614e-04	270.4104	15.7671
ν_2	3094.4	5.283e-07	2.296e-05	2.518e-05	346.3573	3.1399
ν_3	3086.6	3.390e-07	-4.590e-05	-4.528e-04	71.2864	-19.7100
ν_4	3071.5	1.346e-06	-3.188e-05	-2.684e-04	608.0790	-12.1502
ν_5	3065.1	3.306e-06	1.210e-04	1.502e-03	129.7530	62.2482
ν_6	3053.8	-4.915e-08	-6.171e-05	-1.184e-03	383.7959	-45.6995
ν_7	3026.5	-1.589e-06	-1.183e-05	-1.212e-04	163.1129	-5.2295
ν_8	3023.1	2.240e-06	2.377e-05	2.753e-06	970.8641	2.4718

[a] Atomic units.
[b] Units of Å^{4}amu^{-1} for Raman, 10^{-3}Å^{4}amu^{-1} for VROA.

Table SI10: VROA results for (3S)-ethyl-2-oxetanone (950-1900) determined by CCSD/asp-cc-pVDZ using a B3LYP/aug-cc-pVTZ geometry and Hessian.

		VROA Invariants[a]			SCP (180°)[b]	
Mode	ω/cm^{-1}	$\alpha G'$	$\beta(G')^2$	$\beta(A)^2$	$I_R + I_L$	$I_R - I_L$
ν_1	1896.5	-6.072e-07	4.355e-06	9.431e-06	102.3463	0.7509
ν_2	1519.4	1.635e-08	4.339e-06	8.051e-05	17.0708	3.1218
ν_3	1505.9	-9.128e-10	3.436e-06	7.486e-05	4.1250	2.8428
ν_4	1501.5	6.339e-12	2.547e-06	-3.437e-05	20.3742	-0.8920
ν_5	1490.1	1.681e-08	-1.013e-05	-6.818e-05	34.0606	-3.2902
ν_6	1418.0	-1.847e-10	-8.595e-08	-1.041e-06	0.1922	-0.0434
ν_7	1386.5	-1.476e-08	5.820e-07	-1.870e-06	9.7181	-0.0041
ν_8	1353.3	9.168e-09	-1.364e-06	-2.309e-05	6.9342	-0.9074
ν_9	1329.8	-2.148e-08	7.587e-06	-2.127e-05	6.5127	0.0496
ν_{10}	1284.5	-7.537e-10	-3.519e-06	2.582e-05	4.1820	0.5094
ν_{11}	1228.8	3.521e-07	-2.968e-06	4.593e-05	8.5919	1.2358
ν_{12}	1181.7	-7.068e-09	7.350e-06	4.508e-05	1.8673	2.2409
ν_{13}	1154.9	3.009e-08	-6.218e-06	-4.158e-05	12.3554	-2.0107
ν_{14}	1135.7	7.288e-08	-3.111e-06	-3.434e-05	13.7486	-1.4578
ν_{15}	1107.6	-7.040e-08	-1.513e-07	5.848e-06	0.9033	0.1800
ν_{16}	1075.6	-7.780e-08	-4.963e-06	9.239e-06	42.2433	-0.1886
ν_{17}	988.0	1.554e-07	6.334e-06	1.386e-05	13.3617	1.0969
ν_{18}	974.6	6.080e-08	1.812e-06	5.327e-06	5.6706	0.3593
ν_{19}	945.1	-4.636e-07	-2.261e-06	8.716e-06	18.3621	0.0645

[a] Atomic units.
[b] Units of $\mathrm{\AA}^4\mathrm{amu}^{-1}$ for Raman, $10^{-3}\mathrm{\AA}^4\mathrm{amu}^{-1}$ for VROA.

Table SI11: VROA results for (3S)-ethyl-2-oxetanone determined by CCSD/asp-cc-pVDZ using a B3LYP/aug-cc-pVTZ geometry and Hessian.

		VROA Invariants[a]			SCP (180°)[b]	
Mode	ω/cm^{-1}	$\alpha G'$	$\beta(G')^2$	$\beta(A)^2$	$I_R + I_L$	$I_R - I_L$
ν_1	3187.2	2.884e-06	4.447e-05	4.840e-04	346.4309	20.6104
ν_2	3157.5	5.094e-07	1.882e-05	-1.734e-04	402.0436	-3.9045
ν_3	3148.9	2.187e-07	-4.281e-05	-3.683e-04	95.9798	-16.5806
ν_4	3120.4	2.841e-06	1.174e-04	1.853e-03	295.0870	73.5999
ν_5	3105.8	4.345e-06	-1.492e-04	-1.841e-03	730.6714	-76.4050
ν_6	3102.0	-1.443e-06	6.859e-05	1.196e-04	362.4096	10.8604
ν_7	3058.3	3.598e-06	6.843e-07	-3.007e-04	467.3749	-9.9699
ν_8	3050.7	-2.546e-06	5.289e-06	4.149e-05	895.2028	1.9144
ν_9	1807.9	-6.447e-07	9.287e-06	2.441e-05	175.5511	1.7449
ν_{10}	1497.5	-6.697e-09	2.841e-06	8.971e-05	18.9552	3.2788
ν_{11}	1491.0	-3.789e-09	9.652e-06	1.512e-04	11.9907	6.0122
ν_{12}	1487.8	2.143e-09	4.127e-06	-4.552e-05	21.9343	-1.1061
ν_{13}	1474.3	2.763e-08	-1.466e-05	-1.195e-04	34.4042	-5.4577
ν_{14}	1400.2	-1.359e-08	-4.021e-07	-6.878e-06	1.4837	-0.2699
ν_{15}	1376.9	-5.215e-08	1.032e-06	-2.178e-06	16.3896	0.0307
ν_{16}	1329.6	4.797e-08	-1.519e-07	-2.811e-05	8.2709	-0.9534
ν_{17}	1297.2	-8.693e-08	1.079e-05	-3.480e-05	8.1002	-0.0808
ν_{18}	1265.9	-1.195e-09	-5.391e-06	5.421e-05	8.0498	1.2696
ν_{19}	1211.6	4.669e-07	-3.929e-06	8.825e-05	12.7709	2.5523
ν_{20}	1177.2	1.380e-08	7.664e-06	4.747e-05	4.7937	2.3521
ν_{21}	1137.3	1.394e-07	-1.300e-05	-1.062e-04	23.1459	-4.8471
ν_{22}	1131.0	5.663e-08	-2.759e-06	-2.672e-05	12.8098	-1.1683
ν_{23}	1087.7	1.735e-07	-3.541e-06	-3.368e-05	32.5274	-1.4788
ν_{24}	1070.4	-4.004e-07	-3.259e-06	3.424e-05	25.8730	0.8167
ν_{25}	994.0	1.040e-07	9.067e-06	6.800e-05	32.7444	3.1778
ν_{26}	968.3	1.384e-07	2.454e-06	-9.476e-06	4.5048	-0.0706
ν_{27}	922.9	-3.154e-07	-1.084e-06	2.149e-05	7.1987	0.6086
ν_{28}	882.0	3.349e-07	-1.680e-06	-3.666e-05	79.5584	-1.3918
ν_{29}	838.3	-1.409e-06	4.779e-06	3.216e-05	47.7571	1.5520
ν_{30}	787.4	-1.481e-07	2.109e-06	1.214e-05	13.9566	0.6163
ν_{31}	707.3	2.907e-07	-6.555e-06	5.789e-06	41.0528	-0.4631
ν_{32}	662.6	-1.874e-07	5.459e-06	2.337e-06	20.8887	0.6246
ν_{33}	523.5	-3.683e-09	-1.090e-05	-4.567e-05	8.1020	-2.6160
ν_{34}	381.6	1.360e-08	4.847e-06	3.588e-06	6.4101	0.6051
ν_{35}	305.9	-4.113e-08	-1.008e-06	8.707e-06	10.1328	0.1897
ν_{36}	243.1	-1.725e-08	3.104e-07	-1.334e-06	0.4830	-0.0135
ν_{37}	153.3	-9.561e-09	1.562e-06	4.374e-06	6.2488	0.3024
ν_{38}	124.4	-6.441e-10	-7.988e-07	-7.437e-06	1.2339	-0.3282
ν_{39}	106.2	1.361e-08	-4.040e-06	-2.706e-05	2.6293	-1.3079

[a] Atomic units.
[b] Units of Å^{4}amu^{-1} for Raman, 10^{-3}Å^{4}amu^{-1} for VROA.

Table SI12: VROA results for (3S)-propyl-2-oxetanone determined by CC2/asp-cc-pVDZ using a B3LYP/aug-cc-pVTZ geometry and Hessian.

		VROA Invariants[a]			SCP (180°)[b]	
Mode	ω/cm^{-1}	$\alpha G'$	$\beta(G')^2$	$\beta(A)^2$	$I_R + I_L$	$I_R - I_L$
ν_1	3129.7	3.057e-06	3.602e-05	4.711e-04	326.3330	19.3321
ν_2	3089.9	-1.815e-06	-2.795e-06	-3.366e-05	425.4197	-1.4035
ν_3	3083.5	9.880e-07	4.509e-05	6.191e-04	149.1700	25.1812
ν_4	3071.9	1.663e-06	-4.144e-05	-6.528e-04	640.8947	-25.9411
ν_5	3065.7	2.473e-06	-5.527e-05	-6.209e-04	268.8738	-26.2617
ν_6	3052.9	3.313e-07	2.803e-05	1.197e-04	77.8066	6.8041
ν_7	3028.3	6.435e-07	1.069e-05	8.306e-05	451.4141	3.8430
ν_8	3025.6	7.575e-08	9.991e-06	1.208e-04	899.2621	5.0316
ν_9	3014.5	8.148e-07	-2.139e-05	-2.679e-04	104.1040	-11.0860
ν_{10}	3005.7	-9.758e-07	1.476e-05	1.158e-04	799.4638	5.3451
ν_{11}	1896.1	-1.122e-06	4.394e-06	4.231e-05	155.4475	1.8523
ν_{12}	1519.2	-1.485e-08	6.983e-06	1.272e-04	20.1590	4.9455
ν_{13}	1510.7	-1.620e-09	-2.846e-06	-3.240e-05	3.0942	-1.3663
ν_{14}	1500.6	-1.809e-09	-7.887e-07	-1.445e-05	22.3801	-0.5613
ν_{15}	1495.3	2.071e-09	4.160e-06	6.353e-05	11.9452	2.5371
ν_{16}	1486.3	6.612e-08	-1.002e-05	-4.944e-05	54.0524	-2.6539
ν_{17}	1417.5	8.825e-09	-8.364e-08	2.157e-06	0.4637	0.0636
ν_{18}	1397.4	1.462e-09	4.323e-06	7.442e-07	3.9919	0.4578
ν_{19}	1370.0	-2.122e-08	-5.360e-06	-2.219e-05	19.4590	-1.2774
ν_{20}	1338.0	-2.375e-08	7.163e-06	-6.994e-05	9.4331	-1.6172
ν_{21}	1331.2	-4.630e-09	5.509e-06	1.059e-05	11.6521	0.9053
ν_{22}	1312.3	5.516e-08	1.651e-06	1.563e-05	5.0157	0.6868
ν_{23}	1262.5	-1.011e-08	-7.024e-06	-1.571e-05	4.1257	-1.2280
ν_{24}	1221.2	5.831e-07	-5.433e-06	2.597e-05	15.5984	0.3228
ν_{25}	1185.0	-3.454e-10	4.509e-06	2.988e-05	2.0645	1.4489
ν_{26}	1155.9	3.719e-08	-4.235e-06	-7.364e-06	13.8990	-0.6699
ν_{27}	1134.2	-2.296e-08	-1.612e-06	-1.188e-04	19.6996	-4.1282
ν_{28}	1107.6	-2.515e-07	-2.234e-07	2.566e-05	5.3605	0.8340
ν_{29}	1101.6	1.617e-07	-4.198e-06	8.318e-05	41.8579	2.3560
ν_{30}	1042.3	1.285e-07	9.044e-06	6.264e-05	41.1124	2.9965
ν_{31}	1004.7	4.055e-07	6.072e-07	-8.486e-06	10.9517	-0.2225
ν_{32}	953.2	-2.529e-07	2.395e-06	1.951e-05	8.2452	0.8910
ν_{33}	923.6	1.817e-07	6.026e-07	-4.773e-05	74.4450	-1.5329
ν_{34}	899.3	-2.074e-09	-3.124e-06	-7.939e-06	4.5805	-0.5778
ν_{35}	877.5	-7.795e-07	5.405e-06	4.489e-05	24.4489	2.0396
ν_{36}	854.3	-7.235e-07	-1.480e-07	2.086e-05	35.6066	0.6814
ν_{37}	756.3	1.208e-07	1.135e-06	6.430e-06	18.8967	0.3283
ν_{38}	728.6	3.007e-07	-9.348e-06	-6.670e-06	22.7874	-1.1587
ν_{39}	683.2	-1.144e-07	5.724e-06	-2.646e-05	14.7635	-0.3102
ν_{40}	547.4	-5.947e-08	-9.662e-06	-3.707e-05	7.0537	-2.2048
ν_{41}	396.9	1.489e-07	3.392e-06	1.455e-05	8.6616	0.8254
ν_{42}	322.2	-1.225e-07	1.738e-06	1.230e-05	7.1497	0.5846
ν_{43}	269.9	-2.626e-08	-1.217e-06	-1.085e-05	11.1549	-0.4841
ν_{44}	240.7	-5.859e-09	3.424e-08	-5.136e-06	0.1869	-0.1680
ν_{45}	160.3	-1.052e-08	-5.466e-07	-4.631e-06	1.3880	-0.2093
ν_{46}	116.5	-1.782e-09	3.758e-06	1.515e-05	4.9307	0.8821
ν_{47}	90.8	7.774e-10	-3.449e-06	-4.165e-05	1.4795	-1.7358

ν_{48}	80.2	7.294e-09	-5.184e-06	-2.782e-05	3.9144	-1.4477

[a] Atomic units.
[b] Units of $Å^4 amu^{-1}$ for Raman, $10^{-3} Å^4 amu^{-1}$ for VROA.

Table SI13: VROA results for (4R)-methyl-2-oxetanone determined by CC2/asp-cc-pVDZ using a B3LYP/aug-cc-pVTZ geometry and Hessian.

		VROA Invariants[a]			SCP (180°)[b]	
Mode	ω/cm^{-1}	$\alpha G'$	$\beta(G')^2$	$\beta(A)^2$	$I_R + I_L$	$I_R - I_L$
ν_1	3135.5	1.883e-06	-1.010e-05	-2.033e-04	280.6436	-7.7968
ν_2	3107.3	-2.514e-07	-1.000e-04	-1.432e-03	221.3205	-57.8186
ν_3	3097.8	1.726e-06	5.936e-05	1.353e-03	279.3893	51.1113
ν_4	3081.5	-4.202e-06	2.953e-05	3.328e-04	685.3454	14.0670
ν_5	3073.1	3.888e-07	2.925e-05	-1.197e-04	518.2514	-1.0671
ν_6	3032.4	1.579e-06	3.148e-06	-8.548e-05	813.6911	-2.5380
ν_7	1898.7	-8.914e-09	-2.980e-06	4.972e-05	175.7701	1.3612
ν_8	1497.1	-5.689e-10	-3.415e-06	-6.524e-05	22.1195	-2.5198
ν_9	1485.7	1.747e-08	-3.474e-06	1.051e-05	19.4399	0.0029
ν_{10}	1459.6	2.376e-08	3.824e-06	3.061e-05	24.5132	1.4046
ν_{11}	1420.3	1.193e-07	-1.356e-07	-3.873e-07	3.0817	-0.0265
ν_{12}	1388.3	6.588e-08	-2.579e-08	-1.163e-06	6.8848	-0.0414
ν_{13}	1312.4	-1.145e-07	1.224e-06	-1.025e-05	12.1085	-0.2194
ν_{14}	1227.1	1.678e-10	1.885e-06	1.540e-05	5.0137	0.7029
ν_{15}	1202.6	-1.793e-07	-6.810e-07	-2.308e-05	4.6312	-0.8387
ν_{16}	1135.6	1.384e-07	-4.851e-07	-3.989e-05	12.8410	-1.3800
ν_{17}	1113.6	3.259e-07	1.281e-05	2.280e-04	42.2442	8.8944
ν_{18}	1074.0	-8.743e-07	-3.571e-06	-4.591e-05	15.9298	-1.8900
ν_{19}	1042.0	-9.030e-08	-3.082e-06	-7.347e-05	24.8012	-2.7611
ν_{20}	966.1	3.304e-07	-4.059e-06	-2.921e-05	54.9247	-1.3813
ν_{21}	913.5	1.329e-08	1.094e-06	4.845e-06	1.3138	0.2712
ν_{22}	852.2	-5.971e-07	7.409e-06	2.490e-05	42.2612	1.5731
ν_{23}	817.6	-4.762e-09	6.172e-07	6.731e-05	15.0653	2.3085
ν_{24}	711.4	-8.601e-07	-6.819e-06	-4.159e-05	76.4347	-2.0709
ν_{25}	524.0	9.069e-09	4.861e-06	2.601e-05	6.9150	1.3551
ν_{26}	509.0	5.553e-08	-2.400e-06	-2.111e-05	14.4789	-0.9449
ν_{27}	429.3	3.236e-07	3.201e-07	2.310e-06	9.9655	0.1091
ν_{28}	319.6	3.289e-08	-1.447e-06	-1.073e-06	3.9695	-0.1807
ν_{29}	233.0	-7.024e-09	-3.251e-07	1.252e-06	0.1808	0.0092
ν_{30}	116.4	-5.154e-09	7.735e-07	1.023e-05	4.8097	0.4190

[a] Atomic units.
[b] Units of $\mathrm{\mathring{A}}^4\mathrm{amu}^{-1}$ for Raman, $10^{-3}\mathrm{\mathring{A}}^4\mathrm{amu}^{-1}$ for VROA.

Table SI14: VROA results for (4R)-methyl-2-oxetanone (3000+) determined by CCSD/asp-cc-pVDZ using a B3LYP/aug-cc-pVTZ geometry and Hessian.

		VROA Invariants[a]			SCP (180°)[b]	
Mode	ω/cm^{-1}	$\alpha G'$	$\beta(G')^2$	$\beta(A)^2$	$I_R + I_L$	$I_R - I_L$
ν_1	3135.5	1.729e-06	-7.464e-06	-1.869e-04	236.3828	-6.9847
ν_2	3107.3	-3.183e-07	-9.531e-05	-1.251e-03	192.3189	-51.2989
ν_3	3097.8	1.334e-06	4.957e-05	1.127e-03	235.3579	42.5891
ν_4	3081.5	-3.615e-06	2.945e-05	2.911e-04	570.1759	12.6642
ν_5	3073.1	2.951e-07	2.579e-05	-2.577e-05	434.5293	1.7218
ν_6	3032.4	1.066e-06	2.502e-06	-6.751e-05	696.0241	-2.0028

[a] Atomic units.
[b] Units of Å^{4}amu^{-1} for Raman, 10^{-3}Å^{4}amu^{-1} for VROA.

Table SI15: VROA results for (4R)-methyl-2-oxetanone (950-1900) determined by CCSD/asp-cc-pVDZ using a B3LYP/aug-cc-pVTZ geometry and Hessian.

		VROA Invariants[a]			SCP (180°)[b]	
Mode	ω/cm^{-1}	$\alpha G'$	$\beta(G')^2$	$\beta(A)^2$	$I_R + I_L$	$I_R - I_L$
ν_1	1898.7	-3.223e-08	-1.531e-06	3.072e-05	122.6490	0.8722
ν_2	1497.1	-7.305e-10	-2.625e-06	-5.285e-05	20.1306	-2.0271
ν_3	1485.7	1.217e-08	-3.509e-06	1.629e-06	17.3756	-0.2970
ν_4	1459.6	2.851e-09	4.017e-06	3.259e-05	20.5084	1.4901
ν_5	1420.3	8.146e-08	-2.041e-07	-7.944e-07	2.3365	-0.0470
ν_6	1388.3	1.632e-08	-6.118e-07	2.462e-06	5.4288	0.0209
ν_7	1312.4	-2.827e-08	-9.598e-07	-1.856e-05	8.7748	-0.7156
ν_8	1227.1	4.926e-10	1.402e-06	1.063e-05	3.1482	0.4953
ν_9	1202.6	-1.783e-07	-1.656e-06	-2.375e-05	5.1359	-0.9588
ν_{10}	1135.6	6.213e-08	1.387e-06	-2.215e-06	7.7708	0.0649
ν_{11}	1113.6	1.571e-07	9.877e-06	1.527e-04	24.9309	6.0867
ν_{12}	1074.0	-5.023e-07	-2.949e-06	-4.036e-05	11.4760	-1.6424
ν_{13}	1042.0	-1.171e-07	-2.585e-06	-5.120e-05	15.7239	-1.9678
ν_{14}	966.1	2.913e-07	-3.173e-06	-1.694e-05	45.4329	-0.8833

[a] Atomic units.
[b] Units of Å^{4}amu^{-1} for Raman, 10^{-3}Å^{4}amu^{-1} for VROA.

Table SI16: VROA results for (4R)-methyl-2-oxetanone determined by CC2/asp-cc-pVDZ using a CC2/asp-cc-pVDZ geometry and Hessian.

		VROA Invariants[a]			SCP (180°)[b]	
Mode	ω/cm^{-1}	$\alpha G'$	$\beta(G')^2$	$\beta(A)^2$	$I_R + I_L$	$I_R - I_L$
ν_1	3192.5	2.008e-06	1.554e-06	-5.946e-05	295.0849	-1.8292
ν_2	3175.7	-8.529e-07	-9.228e-05	-1.356e-03	260.1743	-54.5042
ν_3	3164.0	1.664e-06	4.380e-05	1.056e-03	266.4967	39.6272
ν_4	3122.1	4.838e-07	1.042e-04	1.175e-04	731.4913	14.3518
ν_5	3111.5	-3.447e-06	-4.475e-05	1.436e-04	501.4946	0.3134
ν_6	3059.7	1.403e-06	2.316e-06	-9.880e-05	868.2303	-3.0659
ν_7	1812.9	1.355e-07	-1.877e-06	4.243e-05	190.9305	1.2282
ν_8	1483.2	-8.636e-10	-3.223e-06	-5.914e-05	23.7534	-2.2967
ν_9	1474.0	6.397e-09	-4.779e-06	-7.740e-06	18.9600	-0.7369
ν_{10}	1440.4	5.985e-08	5.378e-06	3.980e-05	27.2629	1.8671
ν_{11}	1409.0	1.270e-07	5.880e-07	2.768e-05	3.8945	0.9828
ν_{12}	1364.3	3.178e-08	8.246e-08	-2.122e-05	8.8397	-0.7002
ν_{13}	1279.6	-8.949e-08	3.791e-07	-2.049e-05	12.5111	-0.6460
ν_{14}	1209.2	-1.172e-09	3.828e-07	-1.702e-05	7.0536	-0.5299
ν_{15}	1198.3	-1.947e-07	-2.405e-06	-1.716e-05	6.9280	-0.8137
ν_{16}	1130.9	-1.021e-07	1.111e-05	1.367e-04	21.3514	5.6762
ν_{17}	1088.5	4.145e-07	5.262e-06	8.361e-05	34.0304	3.3176
ν_{18}	1055.0	-9.433e-07	-5.269e-06	-6.572e-05	20.0543	-2.7215
ν_{19}	1022.2	-8.441e-08	-1.558e-06	-7.118e-05	23.7022	-2.5319
ν_{20}	956.3	6.462e-07	-5.188e-06	-4.710e-05	68.8553	-2.0918
ν_{21}	896.5	6.403e-08	7.127e-07	8.008e-07	2.5223	0.0981
ν_{22}	840.3	-6.973e-07	6.109e-06	1.192e-04	45.5443	4.5901
ν_{23}	802.0	-3.972e-07	2.258e-06	4.917e-06	17.2057	0.3902
ν_{24}	695.4	-8.928e-07	-6.385e-06	-3.824e-05	86.9722	-1.9156
ν_{25}	500.6	-9.699e-10	5.621e-06	3.153e-05	7.4681	1.6154
ν_{26}	492.7	3.671e-08	-3.718e-06	-2.641e-05	14.2493	-1.2538
ν_{27}	423.7	3.509e-07	6.863e-07	2.609e-06	10.0493	0.1558
ν_{28}	323.4	2.698e-08	-1.348e-06	-1.132e-06	3.9498	-0.1728
ν_{29}	248.0	-8.230e-09	-5.753e-07	4.669e-07	0.4606	-0.0420
ν_{30}	104.6	-6.578e-09	8.467e-07	1.216e-05	5.5015	0.4907

[a] Atomic units.
[b] Units of $\text{Å}^4\text{amu}^{-1}$ for Raman, $10^{-3}\text{Å}^4\text{amu}^{-1}$ for VROA.

Table SI17: VROA results for (4R)-ethyl-2-oxetanone determined by CC2/asp-cc-pVDZ using a B3LYP/aug-cc-pVTZ geometry and Hessian.

		VROA Invariants[a]			SCP (180°)[b]	
Mode	ω/cm^{-1}	$\alpha G'$	$\beta(G')^2$	$\beta(A)^2$	$I_R + I_L$	$I_R - I_L$
ν_1	3135.1	9.187e-07	-4.175e-05	-5.432e-04	260.0333	-22.3120
ν_2	3098.8	-2.173e-06	-3.828e-05	-3.737e-04	426.7871	-16.3065
ν_3	3088.2	3.530e-07	8.845e-05	1.068e-03	115.8882	44.5037
ν_4	3080.0	-2.940e-06	5.277e-06	2.660e-04	563.5288	9.4057
ν_5	3069.7	-1.014e-06	-1.568e-04	-2.488e-03	216.7610	-98.7403
ν_6	3053.5	2.567e-07	8.812e-05	1.548e-03	545.0360	60.4959
ν_7	3028.9	7.708e-07	9.193e-06	3.393e-04	67.2606	12.2457
ν_8	3025.6	-3.057e-06	-1.430e-05	2.183e-04	1287.2732	5.8542
ν_9	1898.4	2.312e-07	4.006e-06	3.877e-05	191.8654	1.6952
ν_{10}	1510.0	4.033e-09	2.784e-06	1.433e-05	1.8261	0.7573
ν_{11}	1502.6	2.714e-08	-1.349e-06	2.088e-06	24.1670	-0.0654
ν_{12}	1492.8	5.780e-09	-1.150e-05	-1.315e-04	48.0896	-5.5422
ν_{13}	1458.6	3.442e-08	9.135e-06	8.000e-05	19.0380	3.5851
ν_{14}	1420.7	-5.277e-10	2.542e-07	5.252e-06	2.3427	0.2008
ν_{15}	1404.8	3.372e-07	-6.488e-06	-7.126e-06	10.2386	-0.8876
ν_{16}	1357.4	-1.241e-07	6.490e-07	4.081e-05	15.3735	1.4273
ν_{17}	1305.0	-2.626e-08	3.940e-07	4.298e-06	4.6083	0.1829
ν_{18}	1281.8	-1.715e-07	-2.224e-06	-7.357e-05	11.1340	-2.6784
ν_{19}	1225.9	-1.202e-08	2.089e-06	7.986e-06	4.9318	0.4758
ν_{20}	1203.0	-6.531e-08	-7.840e-07	-7.732e-06	1.0760	-0.3366
ν_{21}	1138.4	1.868e-08	1.911e-06	-6.064e-06	10.4692	-0.0111
ν_{22}	1118.7	3.303e-07	7.486e-06	1.361e-04	34.0600	5.2942
ν_{23}	1083.5	-1.279e-07	2.217e-06	3.165e-06	5.9201	0.3277
ν_{24}	1059.4	-4.132e-07	3.922e-06	-3.771e-05	73.1540	-0.8660
ν_{25}	988.2	-1.648e-07	-5.197e-06	-2.855e-05	35.1892	-1.4735
ν_{26}	958.7	2.849e-07	-2.664e-06	3.862e-06	14.4481	-0.1379
ν_{27}	948.7	2.687e-07	-1.620e-06	-8.263e-06	6.2061	-0.4380
ν_{28}	887.8	-1.191e-06	6.274e-06	4.195e-05	77.6988	2.0287
ν_{29}	819.4	1.087e-07	-3.927e-06	-5.993e-06	6.0607	-0.5933
ν_{30}	788.4	-1.172e-07	2.672e-07	1.445e-05	10.4944	0.5092
ν_{31}	741.2	-2.857e-08	-4.896e-06	-4.284e-05	41.7738	-1.9204
ν_{32}	528.1	-3.045e-10	6.627e-06	1.789e-05	14.8815	1.2607
ν_{33}	514.4	9.185e-08	-2.256e-06	-1.710e-05	14.5272	-0.7966
ν_{34}	410.9	4.576e-07	3.960e-06	1.489e-05	10.7647	0.8938
ν_{35}	388.6	-1.549e-07	-3.469e-06	-6.173e-06	11.7307	-0.5535
ν_{36}	248.7	-1.598e-08	8.114e-07	1.041e-05	0.9633	0.4288
ν_{37}	201.2	7.107e-10	-1.129e-07	1.598e-06	1.2045	0.0420
ν_{38}	114.3	-2.234e-10	-1.340e-06	-4.803e-06	2.3217	-0.2945
ν_{39}	90.0	-1.200e-08	-4.723e-07	-6.641e-06	4.1058	-0.2690

[a] Atomic units.
[b] Units of Å^{4}amu^{-1} for Raman, 10^{-3}Å^{4}amu^{-1} for VROA.

Table SI18: VROA results for (4R)-ethyl-2-oxetanone (3000+) determined by CCSD/asp-cc-pVDZ using a B3LYP/aug-cc-pVTZ geometry and Hessian.

		VROA Invariants[a]			SCP (180°)[b]	
Mode	ω/cm^{-1}	$\alpha G'$	$\beta(G')^2$	$\beta(A)^2$	$I_R + I_L$	$I_R - I_L$
ν_1	3135.1	9.893e-07	-3.704e-05	-4.787e-04	218.6608	-19.6873
ν_2	3098.8	-1.784e-06	-3.780e-05	-3.274e-04	359.1689	-14.7119
ν_3	3088.2	1.308e-07	8.659e-05	9.451e-04	97.8209	40.2185
ν_4	3080.0	-2.700e-06	7.748e-06	2.372e-04	465.1822	8.6936
ν_5	3069.7	-8.888e-07	-1.522e-04	-2.158e-03	182.7721	-87.2686
ν_6	3053.5	2.326e-07	8.313e-05	1.339e-03	466.3207	53.0089
ν_7	3028.9	7.217e-07	9.789e-06	2.917e-04	58.2818	10.7172
ν_8	3025.6	-2.916e-06	-1.226e-05	1.748e-04	1118.0805	4.6054

[a] Atomic units.
[b] Units of Å^{4}amu^{-1} for Raman, 10^{-3}Å^{4}amu^{-1} for VROA.

Table SI19: VROA results for (4R)-ethyl-2-oxetanone (950-1900) determined by CCSD/asp-cc-pVDZ using a B3LYP/aug-cc-pVTZ geometry and Hessian.

		VROA Invariants[a]			SCP (180°)[b]	
Mode	ω/cm^{-1}	$\alpha G'$	$\beta(G')^2$	$\beta(A)^2$	$I_R + I_L$	$I_R - I_L$
ν_1	1898.4	7.266e-08	3.377e-06	2.110e-05	131.0266	1.0424
ν_2	1510.0	-1.194e-09	2.853e-06	1.298e-05	1.4997	0.7189
ν_3	1502.6	1.375e-08	-9.063e-07	3.929e-06	21.8526	0.0404
ν_4	1492.8	6.873e-09	-1.166e-05	-1.223e-04	42.6217	-5.2487
ν_5	1458.6	1.219e-08	9.213e-06	7.511e-05	15.6375	3.4298
ν_6	1420.7	1.180e-08	-6.363e-07	4.619e-06	1.8922	0.0905
ν_7	1404.8	1.846e-07	-4.929e-06	-2.497e-06	7.9171	-0.5770
ν_8	1357.4	-8.856e-08	-7.595e-07	2.776e-05	10.3569	0.8506
ν_9	1305.0	2.501e-08	-1.276e-06	-2.665e-06	2.5482	-0.2167
ν_{10}	1281.8	-9.124e-08	-3.548e-06	-6.990e-05	10.6303	-2.6886
ν_{11}	1225.9	-6.864e-09	1.183e-06	4.049e-06	3.0362	0.2537
ν_{12}	1203.0	-7.648e-08	-1.593e-06	-8.478e-06	1.4959	-0.4425
ν_{13}	1138.4	8.528e-09	4.909e-06	3.535e-05	9.1193	1.6716
ν_{14}	1118.7	1.736e-07	5.570e-06	8.323e-05	16.5441	3.3360
ν_{15}	1083.5	-6.290e-08	2.452e-06	4.087e-06	4.5295	0.3820
ν_{16}	1059.4	-3.383e-07	3.439e-06	-3.656e-05	55.9008	-0.8759
ν_{17}	988.2	-1.056e-07	-3.299e-06	-1.806e-05	26.0092	-0.9334
ν_{18}	958.7	3.394e-07	-2.963e-06	6.592e-06	13.7459	-0.0766
ν_{19}	948.7	2.385e-07	-5.937e-07	-4.828e-06	5.9306	-0.2206

[a] Atomic units.
[b] Units of $\mathrm{\AA}^4\mathrm{amu}^{-1}$ for Raman, $10^{-3}\mathrm{\AA}^4\mathrm{amu}^{-1}$ for VROA.

Table SI20: VROA results for (4R)-ethyl-2-oxetanone determined by CC2/asp-cc-pVDZ using a CC2/asp-cc-pVDZ geometry and Hessian.

		VROA Invariants[a]			SCP (180°)[b]	
Mode	ω/cm^{-1}	$\alpha G'$	$\beta(G')^2$	$\beta(A)^2$	$I_R + I_L$	$I_R - I_L$
ν_1	3190.8	1.127e-06	-4.211e-05	-5.532e-04	269.7971	-22.6843
ν_2	3162.6	-1.840e-06	-3.929e-05	-3.423e-04	412.0151	-15.3595
ν_3	3152.0	2.084e-07	7.292e-05	8.357e-04	89.3033	35.1988
ν_4	3126.6	-8.058e-07	-1.679e-04	-2.569e-03	348.0365	-102.5712
ν_5	3110.4	-3.285e-06	9.823e-05	8.555e-04	754.4499	38.3915
ν_6	3106.6	7.275e-08	1.716e-05	1.111e-03	357.1206	38.8183
ν_7	3064.6	-4.788e-06	-2.689e-06	6.755e-04	541.8132	22.2771
ν_8	3054.0	1.102e-06	-4.168e-06	1.896e-05	859.6357	0.2155
ν_9	1811.6	3.855e-07	7.285e-06	2.473e-05	207.4489	1.5549
ν_{10}	1495.1	7.973e-09	1.969e-06	1.147e-05	4.5027	0.5801
ν_{11}	1489.4	3.652e-08	-2.836e-06	1.080e-05	26.8219	0.0766
ν_{12}	1478.6	9.759e-09	-1.027e-05	-1.386e-04	49.6489	-5.6567
ν_{13}	1439.8	8.979e-08	9.062e-06	7.009e-05	17.8985	3.2470
ν_{14}	1399.4	-2.222e-08	1.071e-06	1.594e-05	2.7642	0.6393
ν_{15}	1394.0	4.232e-07	-6.576e-06	6.831e-06	14.2935	-0.4305
ν_{16}	1330.0	-8.457e-08	-1.806e-07	2.884e-05	16.0560	0.9444
ν_{17}	1277.5	-8.086e-09	1.524e-06	7.358e-06	3.6362	0.3982
ν_{18}	1265.5	-2.183e-07	-4.724e-06	-1.059e-04	14.0581	-4.0091
ν_{19}	1206.5	3.824e-09	6.752e-07	-2.235e-05	6.4582	-0.6784
ν_{20}	1195.0	-3.719e-08	-8.430e-07	1.326e-05	2.0976	0.3583
ν_{21}	1135.0	-1.015e-07	1.230e-05	1.315e-04	20.1914	5.6205
ν_{22}	1085.5	5.407e-07	1.202e-05	2.398e-05	56.0743	2.0038
ν_{23}	1074.0	-1.535e-08	2.810e-06	1.657e-05	24.6817	0.8346
ν_{24}	1062.3	-6.726e-07	-5.034e-06	-3.773e-05	17.2999	-1.7634
ν_{25}	995.1	-1.093e-07	-9.933e-06	-6.153e-05	44.4859	-3.0485
ν_{26}	949.3	4.304e-07	-1.166e-06	1.610e-06	22.8076	-0.0630
ν_{27}	925.1	4.269e-07	-1.399e-06	-1.651e-05	10.5428	-0.6913
ν_{28}	863.9	-1.930e-06	5.092e-06	8.325e-05	90.5984	3.2887
ν_{29}	816.5	4.917e-08	-3.840e-06	-2.180e-05	13.6027	-1.1120
ν_{30}	784.8	-1.882e-10	2.063e-06	2.754e-05	2.1927	1.1258
ν_{31}	720.8	-5.549e-08	-6.615e-06	-5.598e-05	52.2921	-2.5311
ν_{32}	508.9	2.133e-08	8.617e-06	3.329e-05	20.0788	1.9742
ν_{33}	497.8	6.488e-08	-3.334e-06	-2.608e-05	8.7037	-1.2045
ν_{34}	411.5	4.786e-07	3.804e-06	1.314e-05	8.7216	0.8197
ν_{35}	389.5	-1.695e-07	-3.660e-06	-9.570e-06	14.7924	-0.6859
ν_{36}	252.3	-1.680e-08	4.681e-07	6.831e-06	0.6003	0.2749
ν_{37}	208.5	-4.362e-09	1.334e-07	2.942e-06	1.5049	0.1115
ν_{38}	119.5	-2.972e-09	-1.180e-06	-9.254e-06	1.4965	-0.4270
ν_{39}	86.6	-1.260e-08	-5.279e-07	-1.772e-06	5.9424	-0.1120

[a] Atomic units.
[b] Units of $\text{Å}^4\text{amu}^{-1}$ for Raman, $10^{-3}\text{Å}^4\text{amu}^{-1}$ for VROA.

Table SI21: VROA results for (4R)-propyl-2-oxetanone determined by CC2/asp-cc-pVDZ using a B3LYP/aug-cc-pVTZ geometry and Hessian.

		VROA Invariants[a]			SCP (180°)[b]	
Mode	ω/cm^{-1}	$\alpha G'$	$\beta(G')^2$	$\beta(A)^2$	$I_R + I_L$	$I_R - I_L$
ν_1	3134.5	6.658e-07	-4.265e-05	-6.015e-04	252.9329	-24.3474
ν_2	3090.4	2.256e-06	1.892e-05	3.781e-04	410.6944	14.5139
ν_3	3084.6	-7.415e-07	-7.586e-05	-1.179e-03	146.4823	-46.9376
ν_4	3080.4	-4.191e-06	1.947e-05	5.344e-04	617.0448	19.7867
ν_5	3072.4	1.116e-06	7.584e-05	6.551e-04	293.5902	29.4619
ν_6	3052.8	-4.805e-07	-5.558e-06	2.433e-05	86.5886	0.2555
ν_7	3029.6	-4.628e-07	5.724e-07	1.524e-04	566.2240	5.1447
ν_8	3025.8	-2.136e-06	-1.268e-05	-2.080e-04	926.8244	-8.2130
ν_9	3017.2	-1.136e-06	1.119e-05	2.605e-04	51.2087	9.8174
ν_{10}	3008.5	1.295e-06	-1.154e-05	-1.034e-04	831.0859	-4.6061
ν_{11}	1897.5	-1.032e-07	4.979e-06	1.971e-05	202.6105	1.1564
ν_{12}	1512.1	-5.947e-09	-1.450e-06	-2.306e-05	3.5337	-0.9148
ν_{13}	1501.1	-5.657e-10	2.662e-07	7.142e-06	22.9425	0.2651
ν_{14}	1496.8	3.304e-09	4.913e-06	5.774e-05	7.1104	2.4194
ν_{15}	1489.1	-7.092e-08	-1.233e-05	-1.784e-04	64.2824	-7.1893
ν_{16}	1458.4	5.426e-08	7.821e-06	7.392e-05	19.7526	3.2508
ν_{17}	1418.3	2.409e-08	-8.801e-07	-8.789e-06	1.6244	-0.3815
ν_{18}	1407.8	1.961e-07	-2.207e-06	4.208e-05	6.3727	1.1834
ν_{19}	1388.5	2.748e-08	2.367e-06	5.020e-06	11.8019	0.4046
ν_{20}	1338.7	-2.109e-08	-2.376e-06	8.693e-05	18.8183	2.6636
ν_{21}	1324.8	-3.233e-07	-2.871e-06	-7.324e-05	11.9325	-2.7322
ν_{22}	1282.7	-3.235e-08	-5.468e-06	-2.983e-05	5.6435	-1.5434
ν_{23}	1265.7	-3.147e-08	2.832e-06	1.916e-05	4.5445	0.9232
ν_{24}	1225.1	-6.174e-09	1.596e-06	7.769e-06	5.5671	0.4192
ν_{25}	1202.6	-6.309e-08	-5.403e-07	8.694e-07	1.4712	-0.0251
ν_{26}	1139.2	9.414e-08	-1.886e-06	-5.447e-05	14.9906	-2.0071
ν_{27}	1119.0	1.176e-07	3.942e-06	1.573e-04	28.4685	5.6447
ν_{28}	1095.0	3.355e-07	1.741e-05	5.595e-06	37.8634	1.9302
ν_{29}	1084.2	-7.344e-07	-8.083e-06	-5.879e-05	23.3175	-2.7718
ν_{30}	1041.8	-3.674e-07	-9.645e-06	-2.578e-05	44.9869	-1.8262
ν_{31}	990.2	-3.552e-07	1.767e-06	7.205e-06	33.1267	0.4174
ν_{32}	959.2	5.450e-07	-1.219e-06	-2.709e-05	13.3049	-1.0263
ν_{33}	913.8	-1.104e-06	1.047e-06	3.534e-05	83.1066	1.2845
ν_{34}	896.8	-2.989e-07	-3.662e-07	9.653e-06	22.1869	0.2855
ν_{35}	872.1	1.342e-08	-3.256e-06	-4.499e-05	4.9345	-1.8276
ν_{36}	819.0	1.859e-08	3.671e-06	5.626e-05	16.9588	2.2456
ν_{37}	761.9	-7.329e-08	-1.798e-06	6.899e-07	29.3503	-0.1570
ν_{38}	744.1	-4.939e-08	8.688e-07	-5.395e-06	7.9436	-0.0931
ν_{39}	526.6	2.747e-08	7.184e-06	2.326e-05	13.2187	1.4959
ν_{40}	512.0	1.364e-07	-1.184e-06	-1.576e-05	15.5102	-0.6445
ν_{41}	457.0	6.009e-08	1.666e-06	1.064e-05	0.9736	0.5220
ν_{42}	364.0	-1.956e-07	-4.838e-06	-7.777e-06	14.6820	-0.7441
ν_{43}	313.2	2.394e-07	2.638e-06	9.759e-06	12.5251	0.5899
ν_{44}	244.3	6.394e-10	8.620e-08	-1.930e-06	0.1291	-0.0558
ν_{45}	188.6	-1.567e-08	-9.699e-07	-9.617e-07	1.2630	-0.1292
ν_{46}	102.4	2.910e-10	-1.546e-06	-9.286e-06	3.2112	-0.4648
ν_{47}	93.9	-5.861e-09	7.680e-07	1.545e-06	0.9010	0.1285

ν_{48}	68.9	-3.065e-09	2.128e-06	1.329e-05	6.1567	0.6565

[a] Atomic units.
[b] Units of $\text{Å}^4\text{amu}^{-1}$ for Raman, $10^{-3}\text{Å}^4\text{amu}^{-1}$ for VROA.

Table SI22: VROA results for (3S,4R)-dimethyl-2-oxetanone determined by CC2/asp-cc-pVDZ using a B3LYP/aug-cc-pVTZ geometry and Hessian.

		VROA Invariants[a]			SCP (180°)[b]	
Mode	ω/cm^{-1}	$\alpha G'$	$\beta(G')^2$	$\beta(A)^2$	$I_R + I_L$	$I_R - I_L$
ν_1	3116.3	1.464e-06	2.555e-06	1.252e-04	262.9180	4.4338
ν_2	3108.0	2.167e-08	1.086e-05	9.811e-05	30.3430	4.3623
ν_3	3101.0	6.728e-07	-2.826e-05	-5.175e-04	504.1723	-20.1027
ν_4	3097.9	-8.962e-07	-1.957e-07	3.826e-04	107.0416	12.7515
ν_5	3072.2	4.868e-06	3.875e-05	-3.692e-04	919.3043	-8.4432
ν_6	3056.6	4.984e-08	-1.059e-05	4.979e-05	244.8507	0.6012
ν_7	3039.6	-1.769e-06	3.848e-06	6.581e-05	1313.0496	2.5819
ν_8	3036.2	1.361e-06	-6.017e-07	-6.194e-05	178.0637	-2.1279
ν_9	1891.5	-5.459e-07	6.445e-07	-1.043e-06	163.0195	0.0297
ν_{10}	1507.4	-1.155e-08	4.738e-06	9.423e-05	15.3896	3.6198
ν_{11}	1502.0	1.694e-08	9.144e-06	2.970e-05	21.0289	1.9071
ν_{12}	1493.6	5.671e-10	-1.075e-05	-9.441e-05	29.2104	-4.2283
ν_{13}	1482.6	8.407e-09	-3.967e-06	-2.209e-05	17.1966	-1.1347
ν_{14}	1421.9	8.200e-08	-2.841e-07	8.052e-07	2.9092	-0.0016
ν_{15}	1418.6	2.646e-08	-1.375e-06	-3.342e-06	1.1127	-0.2493
ν_{16}	1389.8	1.092e-07	-2.434e-06	5.037e-06	6.5510	-0.0756
ν_{17}	1354.9	6.793e-08	1.830e-06	-1.645e-05	11.8092	-0.3660
ν_{18}	1311.7	-2.215e-07	-1.553e-06	-4.277e-05	11.8466	-1.5832
ν_{19}	1239.9	-6.078e-08	3.095e-06	5.569e-05	13.3611	2.1690
ν_{20}	1177.2	6.190e-08	4.111e-06	4.004e-05	5.4331	1.7483
ν_{21}	1167.9	6.650e-09	4.636e-06	7.155e-05	10.4756	2.8527
ν_{22}	1131.2	4.468e-08	-6.928e-06	-1.091e-04	26.0689	-4.3345
ν_{23}	1073.8	6.691e-08	5.291e-06	8.863e-05	19.9787	3.4883
ν_{24}	1050.9	-1.055e-07	-1.266e-06	5.340e-05	10.7237	1.6558
ν_{25}	1042.2	-5.935e-08	-5.291e-06	-3.766e-05	16.4599	-1.7870
ν_{26}	1008.9	-7.838e-08	5.358e-07	3.139e-05	8.8092	1.1015
ν_{27}	853.0	8.730e-08	9.047e-06	-1.022e-05	15.3715	0.5649
ν_{28}	834.8	-9.383e-08	1.948e-06	4.854e-05	47.1851	1.8154
ν_{29}	776.2	-4.452e-07	-5.063e-06	4.537e-06	48.0869	-0.3556
ν_{30}	745.5	-1.155e-06	-1.440e-06	7.305e-06	69.9626	0.0997
ν_{31}	677.5	-6.894e-09	8.395e-06	-2.354e-05	11.6144	0.0549
ν_{32}	533.6	4.879e-08	-5.673e-06	-3.769e-05	10.8929	-1.8263
ν_{33}	473.5	4.706e-07	1.238e-06	2.630e-05	12.9921	1.0018
ν_{34}	362.3	7.972e-09	-2.562e-06	-1.588e-05	4.9301	-0.7866
ν_{35}	261.1	1.062e-08	2.063e-07	2.178e-06	1.6554	0.0934
ν_{36}	224.5	-7.476e-08	-4.184e-07	-5.200e-06	5.1533	-0.2155
ν_{37}	207.0	6.101e-09	-9.672e-07	4.398e-06	4.7194	0.0500
ν_{38}	193.1	7.232e-09	8.153e-07	2.499e-08	1.3284	0.0825
ν_{39}	96.5	-2.160e-09	1.389e-07	1.284e-05	3.2354	0.4427

[a] Atomic units.
[b] Units of $Å^4 amu^{-1}$ for Raman, $10^{-3} Å^4 amu^{-1}$ for VROA.

Table SI23: VROA results for (3S,4S)-dimethyl-2-oxetanone determined by CC2/asp-cc-pVDZ using a B3LYP/aug-cc-pVTZ geometry and Hessian.

		VROA Invariants[a]			SCP (180°)[b]	
Mode	ω/cm^{-1}	$\alpha G'$	$\beta(G')^2$	$\beta(A)^2$	$I_R + I_L$	$I_R - I_L$
ν_1	3105.5	-8.693e-07	-5.874e-06	2.604e-04	93.5265	8.1025
ν_2	3103.5	1.498e-06	9.759e-05	1.060e-03	444.3383	45.1683
ν_3	3095.5	-1.256e-06	-1.342e-04	-1.225e-03	337.5843	-54.3332
ν_4	3095.0	5.196e-08	-4.532e-05	-9.014e-04	159.7170	-34.6249
ν_5	3063.0	-1.366e-06	2.822e-04	3.235e-03	252.3861	136.2357
ν_6	3056.2	3.510e-06	-2.228e-04	-2.233e-03	761.7379	-96.8505
ν_7	3032.9	-4.252e-07	-8.654e-07	-1.588e-05	911.5985	-0.6168
ν_8	3030.7	-7.659e-07	-4.144e-06	1.737e-06	603.6417	-0.3570
ν_9	1892.2	-4.244e-07	3.295e-06	3.343e-05	172.1167	1.4457
ν_{10}	1500.2	1.705e-08	1.843e-05	1.656e-04	31.2317	7.3734
ν_{11}	1498.0	-5.692e-09	3.320e-06	1.587e-05	16.8662	0.8620
ν_{12}	1497.1	-2.716e-09	-1.327e-05	-7.153e-05	18.2578	-3.7169
ν_{13}	1484.9	-4.919e-08	-7.274e-08	-8.178e-05	21.6769	-2.7371
ν_{14}	1419.4	-1.419e-07	6.793e-07	1.448e-05	4.1202	0.5512
ν_{15}	1418.5	1.164e-08	1.818e-09	7.761e-08	0.5673	0.0028
ν_{16}	1397.5	-3.641e-08	-3.704e-06	3.915e-06	0.9711	-0.2402
ν_{17}	1344.5	1.670e-09	-2.324e-06	-3.602e-05	8.6376	-1.4352
ν_{18}	1297.3	2.766e-08	2.145e-06	6.957e-05	13.6595	2.5370
ν_{19}	1244.4	9.006e-08	-3.601e-06	3.756e-05	11.0178	0.8931
ν_{20}	1176.1	4.604e-07	6.618e-06	5.755e-05	17.8307	2.5836
ν_{21}	1138.5	-2.722e-07	-2.641e-07	-2.800e-05	13.6383	-0.9610
ν_{22}	1131.5	5.798e-08	-1.662e-05	-1.581e-04	27.4880	-6.9419
ν_{23}	1105.7	-4.944e-08	-9.562e-06	-9.798e-05	5.6250	-4.2281
ν_{24}	1072.7	1.546e-07	8.962e-06	1.369e-04	22.8099	5.4674
ν_{25}	1042.9	5.660e-08	-2.045e-06	1.189e-05	17.0646	0.1922
ν_{26}	997.2	7.260e-11	2.744e-06	4.786e-06	3.8882	0.4345
ν_{27}	852.6	-8.779e-08	9.208e-07	4.973e-05	18.8773	1.7522
ν_{28}	834.8	1.256e-06	-1.968e-06	-1.945e-05	58.5233	-0.8464
ν_{29}	826.7	8.016e-09	-4.604e-06	-9.003e-05	21.0004	-3.4661
ν_{30}	711.4	7.733e-07	7.066e-06	7.907e-05	56.6143	3.3469
ν_{31}	690.0	-1.175e-07	7.577e-06	6.899e-06	15.7161	0.9890
ν_{32}	531.8	-5.517e-08	-1.022e-05	-4.744e-05	16.0999	-2.6073
ν_{33}	464.6	-5.069e-07	4.337e-06	2.715e-05	18.2263	1.3405
ν_{34}	313.9	-6.093e-08	2.576e-07	-1.256e-05	5.4164	-0.3935
ν_{35}	262.3	-1.129e-08	3.531e-07	-6.507e-07	2.8688	0.0136
ν_{36}	228.8	1.948e-09	9.887e-07	-1.062e-05	0.5873	-0.2556
ν_{37}	213.1	-3.164e-09	-8.266e-07	-3.854e-06	2.3303	-0.2114
ν_{38}	197.4	4.721e-09	-2.794e-06	-4.965e-06	6.2881	-0.4455
ν_{39}	105.2	4.228e-09	-5.642e-07	-1.019e-05	2.9842	-0.3966

[a] Atomic units.
[b] Units of Å^{4}amu^{-1} for Raman, 10^{-3}Å^{4}amu^{-1} for VROA.

Table SI24: VROA results for (3S)-ethyl-(4R)-methyl-2-oxetanone determined by CC2/asp-cc-pVDZ using a B3LYP/aug-cc-pVTZ geometry and Hessian.

		VROA Invariants[a]			SCP (180°)[b]	
Mode	ω/cm^{-1}	$\alpha G'$	$\beta(G')^2$	$\beta(A)^2$	$I_R + I_L$	$I_R - I_L$
ν_1	3115.4	1.247e-06	-1.597e-05	-2.321e-05	148.5884	-2.3742
ν_2	3105.7	5.275e-07	-2.672e-05	-1.684e-04	160.6619	-8.2959
ν_3	3098.3	-2.618e-06	4.748e-05	5.776e-04	383.1030	24.0361
ν_4	3090.0	-4.379e-07	1.431e-05	6.444e-05	287.0748	3.5843
ν_5	3070.4	1.322e-06	-6.669e-05	-1.477e-03	820.6426	-55.9759
ν_6	3061.0	4.563e-07	3.409e-05	9.756e-05	124.0407	6.6706
ν_7	3045.3	1.274e-06	-2.477e-05	3.356e-04	399.6901	8.7202
ν_8	3038.5	-3.469e-07	7.441e-06	4.239e-05	945.4242	2.1601
ν_9	3029.0	2.628e-06	-6.865e-06	-9.047e-05	705.5701	-3.7074
ν_{10}	3021.8	-6.083e-06	-1.815e-05	7.032e-04	378.3241	21.6557
ν_{11}	1885.0	-8.895e-07	4.399e-06	1.641e-05	142.6724	0.9882
ν_{12}	1511.7	-1.604e-09	1.060e-06	-3.022e-05	3.7278	-0.9024
ν_{13}	1502.1	-1.028e-08	-5.676e-06	6.270e-05	18.2312	1.5244
ν_{14}	1499.0	-4.853e-10	-1.818e-05	-1.653e-04	28.4647	-7.3394
ν_{15}	1495.5	5.665e-08	1.778e-05	1.393e-04	32.1624	6.4309
ν_{16}	1483.9	2.699e-09	-8.097e-06	-8.285e-05	19.8512	-3.5762
ν_{17}	1421.7	8.613e-08	-7.378e-07	5.533e-06	2.9262	0.1108
ν_{18}	1418.7	2.758e-08	-2.228e-09	-6.739e-06	0.9831	-0.2252
ν_{19}	1392.8	3.321e-08	-2.276e-06	-1.049e-05	9.2001	-0.5782
ν_{20}	1381.9	9.734e-08	1.274e-06	-1.657e-05	15.7683	-0.4256
ν_{21}	1346.3	-8.226e-10	-4.672e-06	-1.077e-05	12.2196	-0.8274
ν_{22}	1307.4	-2.229e-07	1.140e-06	-1.917e-05	12.0222	-0.5257
ν_{23}	1289.1	-6.212e-08	-3.274e-06	-1.309e-06	2.4609	-0.3715
ν_{24}	1229.8	-3.267e-07	2.187e-06	3.653e-05	13.9611	1.4383
ν_{25}	1178.9	2.562e-08	2.349e-06	4.100e-05	2.6286	1.6038
ν_{26}	1162.8	2.333e-08	1.134e-06	3.781e-05	20.6365	1.3756
ν_{27}	1132.8	1.456e-07	-6.762e-06	-8.599e-05	27.0214	-3.5474
ν_{28}	1102.4	-1.416e-07	1.072e-05	1.792e-04	11.1664	7.0553
ν_{29}	1059.6	4.807e-07	2.195e-06	-2.019e-05	29.5811	-0.4540
ν_{30}	1041.1	-4.524e-08	1.096e-06	2.420e-05	14.0713	0.9174
ν_{31}	1035.8	-1.988e-07	4.218e-06	5.753e-05	11.3617	2.3427

[a] Atomic units.
[b] Units of Å^{4}amu^{-1} for Raman, 10^{-3}Å^{4}amu^{-1} for VROA.

Table SI25: VROA results for (3S)-ethyl-(4S)-methyl-2-oxetanone determined by CC2/asp-cc-pVDZ using a B3LYP/aug-cc-pVTZ geometry and Hessian.

		VROA Invariants[a]			SCP (180°)[b]	
Mode	ω/cm^{-1}	$\alpha G'$	$\beta(G')^2$	$\beta(A)^2$	$I_R + I_L$	$I_R - I_L$
ν_1	3106.0	3.370e-07	-3.868e-05	-4.554e-04	155.9855	-19.0753
ν_2	3105.4	-1.174e-06	3.298e-05	8.636e-04	269.4111	32.1301
ν_3	3095.1	-4.007e-07	-8.507e-05	-1.411e-03	286.9731	-55.6149
ν_4	3088.9	-5.149e-07	1.186e-04	1.430e-03	311.0020	59.5935
ν_5	3063.7	-3.178e-07	-1.353e-04	-1.103e-03	358.6265	-50.3483
ν_6	3055.9	-3.505e-06	3.122e-05	1.234e-04	508.2311	7.2468
ν_7	3039.8	2.489e-06	2.607e-05	7.366e-04	465.6858	27.1965
ν_8	3030.6	-8.028e-07	-8.964e-06	-9.909e-06	884.3895	-1.2284
ν_9	3029.9	2.530e-06	-6.518e-06	-1.486e-04	504.6078	-5.6135
ν_{10}	3013.6	-3.723e-06	-2.425e-05	3.299e-04	611.6403	8.5843
ν_{11}	1885.8	-7.723e-07	7.250e-06	7.811e-05	151.4064	3.3331
ν_{12}	1511.2	-6.046e-09	2.792e-06	-1.629e-05	3.9624	-0.2642
ν_{13}	1500.9	2.834e-09	-1.390e-05	-4.612e-06	20.1235	-1.5459
ν_{14}	1497.5	8.207e-09	8.700e-06	1.059e-04	25.2385	4.4070
ν_{15}	1493.4	6.812e-09	-2.890e-07	-1.021e-04	40.1275	-3.4356
ν_{16}	1484.9	-5.303e-08	4.041e-06	-1.355e-05	21.3115	-0.0477
ν_{17}	1421.0	-1.349e-08	1.688e-06	1.186e-05	1.3848	0.5648
ν_{18}	1417.8	-1.452e-07	-1.153e-06	-2.688e-06	2.8600	-0.2052
ν_{19}	1397.1	-1.736e-07	-5.002e-06	1.571e-06	2.3246	-0.4485
ν_{20}	1375.7	2.087e-08	-4.525e-06	1.474e-05	10.9471	0.0389
ν_{21}	1347.8	-1.258e-08	-1.031e-05	-4.551e-05	17.1802	-2.5519
ν_{22}	1306.3	1.668e-07	2.475e-06	6.963e-05	9.5927	2.5721
ν_{23}	1273.4	-1.095e-09	-6.571e-07	-2.373e-05	3.3406	-0.8579
ν_{24}	1232.9	-5.166e-08	-6.887e-07	4.422e-05	9.7446	1.4069
ν_{25}	1174.9	2.141e-07	3.262e-06	2.670e-06	19.6308	0.4158
ν_{26}	1138.6	-2.181e-07	-1.359e-07	-1.035e-05	19.2389	-0.3590
ν_{27}	1123.9	2.917e-07	-1.448e-05	-1.283e-04	38.3883	-5.7336
ν_{28}	1107.9	1.817e-08	-6.014e-06	-5.414e-05	2.7272	-2.4092
ν_{29}	1096.6	-2.565e-08	8.206e-06	1.568e-04	26.3737	6.0542
ν_{30}	1043.5	-5.678e-08	5.687e-06	8.369e-05	12.4596	3.3629
ν_{31}	1036.1	7.200e-08	-3.864e-06	-2.895e-05	13.2840	-1.3534
ν_{32}	974.4	3.332e-08	-2.513e-06	-5.639e-06	8.4898	-0.4399
ν_{33}	878.0	8.284e-07	2.484e-06	2.313e-06	29.1373	0.3259
ν_{34}	876.6	-1.926e-08	-1.656e-07	-1.078e-04	23.0781	-3.6147
ν_{35}	852.7	3.996e-07	-1.574e-06	4.985e-05	22.9534	1.5062
ν_{36}	774.7	7.708e-07	7.797e-07	-8.675e-06	25.8702	-0.2115
ν_{37}	708.4	7.905e-07	7.740e-06	8.372e-05	50.0521	3.5696
ν_{38}	695.0	3.496e-08	6.217e-06	-1.990e-06	10.5274	0.5562
ν_{39}	533.0	5.933e-08	-9.490e-06	-5.383e-05	17.3437	-2.7471
ν_{40}	457.4	-5.262e-07	3.135e-06	1.842e-05	19.0545	0.9289
ν_{41}	373.0	9.129e-10	4.065e-06	2.760e-05	10.9027	1.3283
ν_{42}	299.7	-8.485e-08	-2.106e-06	-1.582e-05	6.4903	-0.7390
ν_{43}	261.3	-1.452e-10	-1.653e-06	2.505e-07	1.7584	-0.1572
ν_{44}	227.4	6.088e-09	2.666e-07	-9.271e-07	0.5817	-0.0042
ν_{45}	199.9	1.380e-08	1.783e-07	-5.049e-06	1.6652	-0.1507
ν_{46}	151.9	-7.772e-09	-1.935e-07	2.649e-07	4.3743	-0.0105
ν_{47}	110.0	9.704e-10	-2.473e-06	-2.319e-05	2.7973	-1.0217

ν_{48}	82.6	9.176e-09	3.434e-06	2.062e-05	1.3600	1.0322

[a] Atomic units.
[b] Units of $\mathring{A}^4 amu^{-1}$ for Raman, $10^{-3}\mathring{A}^4 amu^{-1}$ for VROA.

Table SI26: VROA results for (3S)-methyl-(4R)-ethyl-2-oxetanone determined by CC2/asp-cc-pVDZ using a B3LYP/aug-cc-pVTZ geometry and Hessian.

		VROA Invariants[a]			SCP (180°)[b]	
Mode	ω/cm^{-1}	$\alpha G'$	$\beta(G')^2$	$\beta(A)^2$	$I_R + I_L$	$I_R - I_L$
ν_1	3110.9	4.492e-07	3.149e-06	-1.735e-04	163.4678	-5.4753
ν_2	3104.3	-9.833e-08	4.399e-05	6.604e-04	167.2729	26.4496
ν_3	3100.8	1.302e-06	-7.601e-05	-5.226e-04	349.0732	-25.0564
ν_4	3091.9	3.312e-07	2.232e-05	-4.529e-05	293.9266	0.7231
ν_5	3069.1	1.033e-05	1.494e-04	1.897e-03	408.8287	78.2926
ν_6	3060.9	-3.169e-06	-1.045e-04	-1.105e-03	563.8126	-47.3639
ν_7	3048.0	-3.686e-07	2.561e-05	-3.735e-04	356.6424	-9.9022
ν_8	3039.2	-1.066e-06	-1.402e-06	-4.087e-05	799.1679	-1.5046
ν_9	3031.1	-2.473e-06	1.064e-06	1.118e-04	781.4081	3.8368
ν_{10}	3020.2	5.343e-06	1.814e-05	-8.887e-04	440.0435	-27.8465
ν_{11}	1890.9	-7.655e-07	-2.391e-06	3.025e-05	176.0784	0.7704
ν_{12}	1508.8	1.009e-08	1.945e-06	3.476e-05	12.5969	1.3550
ν_{13}	1502.5	-1.287e-09	-4.311e-06	-8.178e-06	14.8169	-0.7047
ν_{14}	1500.4	5.915e-09	-9.767e-06	-1.550e-04	36.6373	-6.1529
ν_{15}	1499.8	3.997e-09	2.332e-05	1.639e-04	17.4300	7.8074
ν_{16}	1476.8	-6.215e-08	-7.593e-07	-3.096e-05	27.9827	-1.1096
ν_{17}	1421.5	7.510e-08	-5.314e-07	8.028e-06	3.8240	0.2148
ν_{18}	1417.0	3.177e-08	-3.331e-06	6.324e-06	3.5956	-0.1224
ν_{19}	1414.3	2.547e-07	-2.812e-06	9.430e-06	8.8443	0.0331
ν_{20}	1359.7	-1.673e-08	-2.660e-07	-3.662e-05	13.3560	-1.2491
ν_{21}	1347.6	-4.786e-08	-1.582e-06	-4.275e-05	3.5790	-1.5854
ν_{22}	1310.6	-1.333e-07	4.347e-07	1.947e-05	1.0155	0.6933
ν_{23}	1281.1	-3.484e-09	1.578e-06	3.303e-05	16.2102	1.2606
ν_{24}	1235.1	-3.218e-08	1.363e-07	3.556e-05	11.8776	1.2007
ν_{25}	1186.1	1.278e-07	9.160e-06	6.865e-05	8.3554	3.2088
ν_{26}	1161.3	2.927e-09	4.201e-06	6.505e-05	12.5851	2.5921
ν_{27}	1132.7	1.243e-07	-9.794e-06	-1.221e-04	31.8603	-5.0560
ν_{28}	1090.2	-1.565e-08	3.625e-07	-5.934e-05	10.1102	-1.9443
ν_{29}	1072.5	-5.181e-08	5.078e-06	1.329e-04	26.5330	4.9444
ν_{30}	1054.6	-5.218e-07	-1.687e-05	-6.468e-05	22.5049	-3.8480
ν_{31}	1033.7	8.792e-08	-1.427e-06	5.324e-05	6.6843	1.6342
ν_{32}	964.6	7.306e-08	1.727e-05	2.902e-05	15.1746	2.6982

[a] Atomic units.
[b] Units of $\mathrm{\AA}^4\mathrm{amu}^{-1}$ for Raman, $10^{-3}\mathrm{\AA}^4\mathrm{amu}^{-1}$ for VROA.

Table SI27: VROA results for (3S)-methyl-(4S)-ethyl-2-oxetanone determined by CC2/asp-cc-pVDZ using a B3LYP/aug-cc-pVTZ geometry and Hessian.

		VROA Invariants[a]			SCP (180°)[b]	
Mode	ω/cm^{-1}	$\alpha G'$	$\beta(G')^2$	$\beta(A)^2$	$I_R + I_L$	$I_R - I_L$
ν_1	3104.2	1.041e-06	1.597e-04	8.009e-04	213.0499	42.7241
ν_2	3103.7	9.707e-07	-1.826e-04	-1.292e-03	306.5474	-61.4240
ν_3	3095.7	-1.316e-06	-9.459e-05	-8.386e-04	230.4133	-37.4656
ν_4	3090.7	-3.928e-07	1.169e-04	1.821e-03	292.1286	72.4773
ν_5	3062.6	-4.264e-06	-1.626e-04	-2.326e-03	173.0495	-93.9203
ν_6	3054.9	2.944e-07	1.261e-04	1.124e-03	580.4106	50.1524
ν_7	3042.0	2.021e-06	-1.122e-05	6.688e-04	616.0669	21.2005
ν_8	3034.2	-5.263e-07	5.431e-06	3.049e-05	978.9510	1.5615
ν_9	3030.9	2.684e-06	-7.368e-06	-2.245e-04	381.8144	-8.2322
ν_{10}	3013.0	-2.662e-06	-2.004e-05	5.868e-04	567.0913	17.5789
ν_{11}	1890.5	-1.418e-07	4.537e-06	-6.378e-06	185.3880	0.2414
ν_{12}	1508.3	-1.176e-08	1.471e-06	-1.377e-05	13.0740	-0.3122
ν_{13}	1500.5	2.043e-08	-2.429e-05	-1.193e-04	16.7547	-6.4161
ν_{14}	1499.7	2.194e-09	2.320e-05	1.602e-04	36.1401	7.6709
ν_{15}	1497.9	1.104e-09	3.698e-06	8.585e-06	17.2128	0.6569
ν_{16}	1476.8	-7.797e-09	-1.393e-06	-7.727e-06	35.2385	-0.3974
ν_{17}	1421.3	-1.321e-07	-6.097e-07	5.332e-06	1.5593	0.1169
ν_{18}	1418.5	1.539e-09	1.649e-07	2.285e-06	0.3419	0.0928
ν_{19}	1412.3	-3.428e-07	-3.738e-06	1.074e-05	6.9303	-0.0159
ν_{20}	1357.0	8.885e-08	-5.786e-06	-2.129e-06	8.4444	-0.6505
ν_{21}	1349.6	4.761e-10	7.819e-06	9.543e-05	7.6758	3.9685
ν_{22}	1294.5	1.879e-07	-1.488e-06	-2.415e-05	2.8814	-0.9552
ν_{23}	1277.3	3.772e-08	2.905e-07	-7.136e-05	16.2698	-2.3527
ν_{24}	1242.8	-2.379e-08	-2.372e-06	2.641e-05	12.6687	0.6440
ν_{25}	1180.1	3.192e-07	5.173e-06	3.000e-05	13.4183	1.5193
ν_{26}	1136.8	-2.498e-07	-1.714e-06	-7.980e-05	17.2530	-2.8354
ν_{27}	1125.6	6.293e-08	-1.321e-05	-8.126e-05	20.1449	-4.0350
ν_{28}	1105.0	-1.971e-07	-2.648e-06	-3.671e-05	31.4183	-1.4906
ν_{29}	1079.7	3.400e-07	3.209e-06	6.627e-05	22.0031	2.5333
ν_{30}	1070.7	2.618e-08	-1.844e-06	6.467e-05	21.1850	1.9739
ν_{31}	1037.1	3.985e-08	5.352e-06	2.643e-05	6.7431	1.4181
ν_{32}	964.9	1.570e-08	-7.766e-06	-5.942e-06	11.3411	-0.9760

[a] Atomic units.
[b] Units of $\text{Å}^4\text{amu}^{-1}$ for Raman, $10^{-3}\text{Å}^4\text{amu}^{-1}$ for VROA.

Table SI28: VROA results for (3S,4S)-dimethyl)-2-oxetanone determined by CC2/asp-cc-pVDZ using a CC2/asp-cc-pVDZ geometry and Hessian.

		VROA Invariants[a]			SCP (180°)[b]	
Mode	ω/cm^{-1}	$\alpha G'$	$\beta(G')^2$	$\beta(A)^2$	$I_R + I_L$	$I_R - I_L$
ν_1	3173.5	-2.128e-07	3.368e-05	6.892e-04	259.4798	26.3772
ν_2	3167.9	9.020e-07	3.553e-05	3.684e-04	311.9181	15.8551
ν_3	3161.3	-4.587e-07	-4.126e-05	-8.796e-04	240.3611	-33.4929
ν_4	3160.5	-8.830e-07	-8.921e-05	-6.618e-04	248.6763	-31.0253
ν_5	3106.8	-1.177e-06	2.440e-04	2.912e-03	207.5135	121.6284
ν_6	3101.1	4.643e-06	-2.293e-04	-2.235e-03	754.1218	-97.5719
ν_7	3058.2	-3.994e-07	-2.986e-07	-3.814e-06	1108.3311	-0.1572
ν_8	3057.8	-8.497e-08	-3.170e-07	2.135e-05	505.8689	0.6809
ν_9	1803.9	-8.171e-07	6.855e-07	7.133e-05	191.5285	2.4497
ν_{10}	1486.8	3.324e-08	1.767e-05	1.108e-04	30.4041	5.4672
ν_{11}	1484.3	-4.170e-09	1.189e-05	9.422e-05	21.9239	4.3361
ν_{12}	1482.3	-7.520e-09	-1.556e-05	-5.327e-05	14.8487	-3.3366
ν_{13}	1472.2	-3.536e-08	-3.176e-06	-1.173e-04	20.8498	-4.2344
ν_{14}	1411.9	-1.377e-07	-1.662e-06	1.500e-05	4.6520	0.3342
ν_{15}	1393.9	-2.942e-09	-6.473e-07	-2.643e-06	0.5943	-0.1530
ν_{16}	1380.1	3.356e-08	-1.205e-06	3.871e-06	0.7773	0.0085
ν_{17}	1321.3	-1.997e-08	3.363e-07	-6.956e-06	8.8461	-0.1985
ν_{18}	1262.2	4.126e-08	5.480e-06	8.238e-05	13.2202	3.2985
ν_{19}	1227.9	1.249e-07	-9.526e-07	6.344e-05	10.0324	2.0221
ν_{20}	1172.9	3.407e-07	7.529e-06	4.631e-05	22.1146	2.2997
ν_{21}	1141.0	1.571e-07	-1.814e-05	-1.590e-04	29.5354	-7.1228
ν_{22}	1124.4	2.030e-08	-9.810e-06	-4.259e-05	4.3788	-2.4039
ν_{23}	1099.1	-1.635e-07	-8.850e-06	-1.819e-04	15.4805	-6.9593
ν_{24}	1050.2	8.105e-08	1.370e-05	1.938e-04	36.9521	7.8424
ν_{25}	1017.0	-9.259e-09	-5.439e-06	6.147e-06	13.3657	-0.3395
ν_{26}	983.9	-3.220e-09	2.716e-06	-4.347e-07	4.2461	0.2574
ν_{27}	840.7	-4.776e-07	-8.670e-07	-7.712e-05	32.1355	-2.6611
ν_{28}	827.9	1.086e-06	-3.329e-07	-1.656e-05	58.1156	-0.5862
ν_{29}	808.1	8.527e-07	-3.050e-06	2.408e-05	28.6117	0.4984
ν_{30}	690.2	6.400e-07	4.541e-07	1.638e-05	52.4790	0.5923
ν_{31}	679.1	1.295e-07	1.101e-05	5.133e-05	26.8938	2.8155
ν_{32}	514.0	-1.290e-07	-8.626e-06	-4.257e-05	13.7748	-2.2849
ν_{33}	461.8	-4.724e-07	4.030e-06	2.074e-05	16.7647	1.0957
ν_{34}	315.3	-4.992e-08	-4.155e-08	-1.459e-05	4.6204	-0.4911
ν_{35}	254.6	-1.209e-08	9.925e-07	3.803e-07	3.0117	0.1121
ν_{36}	244.8	1.996e-09	1.911e-07	-5.253e-06	0.4620	-0.1562
ν_{37}	228.5	-2.400e-09	-5.921e-07	-1.202e-06	1.0308	-0.0994
ν_{38}	191.8	-1.799e-10	-2.443e-06	-8.193e-06	7.1207	-0.5181
ν_{39}	91.7	5.794e-09	-6.143e-07	-1.419e-05	3.3191	-0.5352

[a] Atomic units.
[b] Units of Å^{4}amu^{-1} for Raman, 10^{-3}Å^{4}amu^{-1} for VROA.

Table SI29: VROA results for (3S,4R)-dimethyl-2-oxetanone determined by CC2/asp-cc-pVDZ using a CC2/asp-cc-pVDZ geometry and Hessian.

		VROA Invariants[a]			SCP (180°)[b]	
Mode	ω/cm^{-1}	$\alpha G'$	$\beta(G')^2$	$\beta(A)^2$	$I_R + I_L$	$I_R - I_L$
ν_1	3182.6	9.549e-07	4.909e-06	2.827e-04	275.9515	9.9280
ν_2	3172.3	6.079e-08	-1.844e-05	-6.460e-04	47.6167	-23.4085
ν_3	3165.8	2.420e-07	2.259e-05	4.955e-04	567.6331	18.8034
ν_4	3163.2	-9.209e-07	-3.483e-05	-8.199e-05	41.9421	-6.2247
ν_5	3117.7	5.431e-06	5.122e-05	-3.562e-04	903.1813	-6.7618
ν_6	3102.4	1.195e-07	-1.243e-05	3.349e-05	243.5037	-0.1271
ν_7	3065.7	-8.378e-07	5.027e-06	4.783e-05	1461.6009	2.0998
ν_8	3061.6	7.507e-07	1.214e-06	-5.365e-05	80.6424	-1.6691
ν_9	1803.7	-5.980e-07	1.515e-07	-4.201e-06	181.1470	-0.1251
ν_{10}	1494.2	-2.005e-08	5.578e-06	9.084e-05	15.4407	3.5907
ν_{11}	1488.7	1.184e-08	1.145e-05	4.815e-05	18.5592	2.7541
ν_{12}	1476.4	2.570e-10	-1.150e-05	-8.960e-05	30.8888	-4.1421
ν_{13}	1466.3	4.440e-09	-4.562e-06	-4.348e-05	16.9666	-1.9081
ν_{14}	1406.6	1.492e-07	-1.266e-06	7.168e-06	2.6739	0.1125
ν_{15}	1395.4	4.469e-09	-8.116e-07	-1.508e-05	0.9512	-0.5847
ν_{16}	1365.1	1.683e-07	-1.087e-06	-1.113e-06	3.6314	-0.1460
ν_{17}	1344.0	7.101e-09	1.685e-06	7.438e-06	16.9054	0.4171
ν_{18}	1274.9	-2.589e-07	-5.734e-06	-8.343e-05	12.5526	-3.3591
ν_{19}	1221.6	-7.299e-08	2.759e-06	8.940e-05	15.7436	3.2603
ν_{20}	1189.3	-3.954e-08	5.224e-06	4.856e-05	15.5391	2.1440
ν_{21}	1173.3	2.982e-08	3.987e-06	5.459e-05	2.6314	2.2214
ν_{22}	1092.6	1.641e-07	-7.500e-06	-1.395e-04	17.9107	-5.4067
ν_{23}	1048.8	2.495e-07	-2.821e-06	-4.212e-05	26.2339	-1.6883
ν_{24}	1048.3	-4.554e-07	8.758e-06	1.735e-04	22.1793	6.6700
ν_{25}	1014.7	-1.254e-09	-4.972e-06	-2.362e-05	10.2468	-1.2864
ν_{26}	998.5	-1.291e-07	5.152e-07	3.275e-05	12.4552	1.1446
ν_{27}	827.3	1.364e-08	3.564e-06	5.613e-05	36.5345	2.2304
ν_{28}	815.2	5.132e-07	8.603e-06	-2.605e-06	30.9996	0.7746
ν_{29}	793.7	-1.550e-06	-7.807e-06	-9.153e-06	74.6981	-1.0873
ν_{30}	719.6	-6.230e-07	6.644e-07	1.124e-05	67.3808	0.4419
ν_{31}	658.7	-3.758e-09	4.950e-06	-1.750e-05	9.8165	-0.0886
ν_{32}	517.7	-1.798e-08	-5.549e-06	-3.081e-05	9.7707	-1.5839
ν_{33}	465.1	4.747e-07	1.518e-06	1.505e-05	11.6353	0.6545
ν_{34}	356.6	-6.985e-09	-2.274e-06	-1.460e-05	4.8478	-0.7150
ν_{35}	289.4	1.100e-08	3.093e-07	1.490e-06	1.1754	0.0807
ν_{36}	224.4	-5.635e-08	-1.022e-06	8.242e-07	2.1324	-0.0748
ν_{37}	205.3	-2.555e-08	-1.989e-07	4.756e-07	5.9350	-0.0040
ν_{38}	194.1	1.334e-08	9.953e-07	1.017e-06	2.3334	0.1336
ν_{39}	84.1	-3.081e-09	1.626e-07	1.814e-05	3.4294	0.6217

[a] Atomic units.
[b] Units of $\text{Å}^4\text{amu}^{-1}$ for Raman, $10^{-3}\text{Å}^4\text{amu}^{-1}$ for VROA.

Table SI30: VROA results for (3S)-d-2-oxetanone determined by CC2/asp-cc-pVDZ using a B3LYP/aug-cc-pVTZ geometry and Hessian.

		VROA Invariants[a]			SCP (180°)[b]	
Mode	ω/cm^{-1}	$\alpha G'$	$\beta(G')^2$	$\beta(A)^2$	$I_R + I_L$	$I_R - I_L$
ν_1	3138.6	1.278e-06	5.039e-05	4.387e-05	337.2845	6.5103
ν_2	3115.2	4.450e-06	-5.266e-05	-1.083e-04	468.6691	-8.8888
ν_3	3081.0	-2.542e-07	9.665e-06	4.478e-05	636.2316	2.4624
ν_4	2289.1	-2.397e-06	-3.299e-06	3.434e-05	226.5249	0.8161
ν_5	1905.0	1.586e-08	1.008e-06	-2.371e-06	161.3224	0.0218
ν_6	1522.3	3.021e-10	-6.635e-08	-3.467e-07	17.0037	-0.0182
ν_7	1342.4	3.642e-09	-5.439e-07	-1.794e-06	6.0693	-0.1144
ν_8	1311.3	-1.102e-07	-4.158e-06	-9.016e-06	20.1793	-0.7173
ν_9	1206.3	4.262e-08	-5.050e-06	-2.363e-05	5.3263	-1.2945
ν_{10}	1159.9	-1.944e-08	4.942e-06	4.033e-05	16.3770	1.8409
ν_{11}	1111.9	7.939e-07	2.139e-06	-3.799e-05	24.4737	-1.0539
ν_{12}	1096.2	-2.739e-08	2.020e-06	2.144e-05	1.0567	0.9179
ν_{13}	988.3	-1.571e-06	-3.504e-06	-3.589e-05	81.7066	-1.5488
ν_{14}	970.4	4.854e-07	1.520e-06	1.614e-05	36.0746	0.6911
ν_{15}	924.9	1.600e-07	4.220e-06	-1.815e-06	45.6515	0.3620
ν_{16}	846.6	2.610e-09	-5.941e-07	3.459e-05	13.3106	1.0952
ν_{17}	754.7	3.181e-07	3.352e-06	5.368e-05	50.9341	2.1276
ν_{18}	706.4	-1.557e-07	-5.423e-06	-6.902e-05	10.6672	-2.8467
ν_{19}	493.0	2.838e-09	-6.715e-06	-2.897e-05	13.1997	-1.6396
ν_{20}	462.4	-1.138e-08	6.324e-06	2.840e-05	10.8178	1.5813
ν_{21}	183.4	6.148e-10	-6.133e-08	-1.739e-07	0.5065	-0.0119

[a] Atomic units.
[b] Units of Å^{4}amu^{-1} for Raman, 10^{-3}Å^{4}amu^{-1} for VROA.

Table SI31: VROA results for (4R)-d-2-oxetanone determined by CC2/asp-cc-pVDZ using a B3LYP/aug-cc-pVTZ geometry and Hessian.

		VROA Invariants[a]			SCP (180°)[b]	
Mode	ω/cm^{-1}	$\alpha G'$	$\beta(G')^2$	$\beta(A)^2$	$I_R + I_L$	$I_R - I_L$
ν_1	3142.2	1.465e-06	-3.374e-05	-1.967e-04	265.7716	-9.9437
ν_2	3109.5	1.909e-06	6.829e-05	-2.149e-05	536.8569	6.1218
ν_3	3088.8	-2.937e-07	-1.499e-05	-1.573e-05	579.8553	-2.0263
ν_4	2287.0	-2.048e-06	-9.409e-06	1.230e-04	257.9223	3.1624
ν_5	1904.6	2.479e-08	1.292e-07	5.012e-06	153.9091	0.1802
ν_6	1462.5	1.399e-09	2.567e-07	-4.788e-06	25.2774	-0.1341
ν_7	1373.8	1.111e-07	-1.016e-06	2.501e-05	14.0945	0.7330
ν_8	1292.5	-8.364e-09	2.394e-06	1.173e-05	7.1350	0.6315
ν_9	1225.4	1.142e-08	9.562e-07	1.155e-05	2.9738	0.4813
ν_{10}	1158.2	-2.805e-08	-1.781e-06	-7.780e-05	8.3280	-2.7754
ν_{11}	1110.2	-3.396e-08	2.234e-06	7.060e-05	18.5339	2.5803
ν_{12}	1044.9	-1.291e-06	4.712e-07	-2.396e-05	21.2240	-0.7527
ν_{13}	1001.7	9.893e-07	1.685e-06	5.212e-05	79.8363	1.9087
ν_{14}	938.3	-8.789e-08	-2.697e-06	-4.676e-05	50.4626	-1.8310
ν_{15}	911.6	-4.778e-08	-9.111e-06	-3.811e-05	7.8952	-2.1845
ν_{16}	860.2	-1.429e-09	3.602e-06	3.879e-05	23.7458	1.6554
ν_{17}	744.3	-1.693e-07	7.402e-07	-1.829e-05	56.8348	-0.5363
ν_{18}	710.5	3.667e-07	3.351e-06	1.727e-05	11.7088	0.9120
ν_{19}	520.9	1.071e-10	-2.839e-07	-5.346e-07	4.0368	-0.0463
ν_{20}	488.8	3.291e-09	-6.844e-07	-1.317e-06	19.2048	-0.1125
ν_{21}	178.3	-7.028e-10	2.733e-08	6.822e-08	0.8641	0.0050

[a] Atomic units.
[b] Units of Å^{4}amu^{-1} for Raman, 10^{-3}Å^{4}amu^{-1} for VROA.

Table SI32: VROA results for (3S)-d-(4S)-d-2-oxetanone determined by CC2/asp-cc-pVDZ using a B3LYP/aug-cc-pVTZ geometry and Hessian.

		VROA Invariants[a]			SCP (180°)[b]	
Mode	ω/cm^{-1}	$\alpha G'$	$\beta(G')^2$	$\beta(A)^2$	$I_R + I_L$	$I_R - I_L$
ν_1	3117.2	3.131e-06	1.211e-04	3.989e-04	262.0570	25.4445
ν_2	3109.0	-7.693e-07	-1.330e-04	-1.802e-04	667.9345	-19.3355
ν_3	2290.7	-1.566e-06	7.081e-05	2.564e-04	347.0845	15.6502
ν_4	2285.8	1.215e-06	-6.505e-05	-3.495e-04	137.0849	-18.1819
ν_5	1904.3	-6.417e-09	9.827e-07	-8.985e-06	154.2941	-0.2015
ν_6	1377.4	-1.445e-07	7.719e-07	-3.110e-06	8.0681	-0.0265
ν_7	1311.7	-1.681e-08	-7.014e-06	-6.959e-05	19.8627	-3.0254
ν_8	1282.1	5.906e-09	6.954e-07	4.809e-05	11.4528	1.6748
ν_9	1192.7	-2.882e-08	-4.284e-07	4.442e-05	6.2893	1.4398
ν_{10}	1110.7	-3.051e-09	-1.399e-06	-3.961e-05	14.5784	-1.4623
ν_{11}	1073.8	1.189e-06	2.221e-06	-2.909e-05	42.3382	-0.7485
ν_{12}	970.8	4.625e-07	-9.442e-07	-3.880e-06	20.7219	-0.2241
ν_{13}	958.9	-6.655e-07	1.415e-06	-4.789e-05	22.5716	-1.4568
ν_{14}	928.7	-6.763e-07	2.609e-06	4.610e-05	79.9919	1.7999
ν_{15}	887.8	-3.302e-08	5.878e-06	1.637e-05	10.2712	1.1352
ν_{16}	809.7	-1.102e-10	9.109e-07	3.311e-05	28.2964	1.1963
ν_{17}	739.0	2.098e-07	-2.470e-06	2.005e-05	45.3140	0.4220
ν_{18}	663.4	-1.764e-07	-4.405e-06	-4.321e-05	7.8340	-1.8835
ν_{19}	489.9	-1.539e-09	-6.128e-06	-2.850e-05	11.9218	-1.5650
ν_{20}	461.5	-1.066e-08	6.626e-06	2.944e-05	11.3023	1.6462
ν_{21}	174.2	9.190e-10	-6.879e-08	-7.094e-08	0.8214	-0.0093

[a] Atomic units.
[b] Units of Å^{4}amu^{-1} for Raman, 10^{-3}Å^{4}amu^{-1} for VROA.

Table SI33: VROA results for (3S)-d-(4R)-d-2-oxetanone determined by CC2/asp-cc-pVDZ using a B3LYP/aug-cc-pVTZ geometry and Hessian.

		VROA Invariants[a]			SCP (180°)[b]	
Mode	ω/cm^{-1}	$\alpha G'$	$\beta(G')^2$	$\beta(A)^2$	$I_R + I_L$	$I_R - I_L$
ν_1	3118.8	8.685e-06	1.484e-05	-3.839e-04	625.4177	-11.3273
ν_2	3107.7	-8.676e-08	1.241e-05	1.294e-04	299.8278	5.5621
ν_3	2291.6	-4.384e-06	-7.494e-06	2.180e-04	342.2729	6.5252
ν_4	2284.6	-1.435e-07	-5.306e-06	-5.681e-05	146.4530	-2.4275
ν_5	1904.3	4.703e-08	1.397e-06	-2.420e-06	154.3080	0.0591
ν_6	1374.6	1.320e-07	-1.165e-06	1.085e-05	9.8807	0.2455
ν_7	1312.0	-9.813e-08	-3.323e-06	-4.892e-07	20.6407	-0.3491
ν_8	1292.3	-9.066e-09	1.771e-06	1.166e-06	7.2734	0.2162
ν_9	1196.3	-4.612e-09	-2.888e-07	5.514e-06	4.3785	0.1551
ν_{10}	1114.8	-2.656e-09	2.432e-06	-5.556e-06	17.4257	0.0580
ν_{11}	1011.7	8.374e-07	3.238e-07	-6.493e-08	20.7790	0.0303
ν_{12}	988.9	-1.710e-06	-1.749e-06	-7.269e-06	68.4677	-0.4177
ν_{13}	967.6	1.932e-07	3.423e-06	2.484e-05	35.6332	1.1720
ν_{14}	936.2	7.419e-08	-2.090e-06	-4.208e-05	44.1783	-1.6140
ν_{15}	891.1	4.853e-09	-7.248e-07	-8.321e-06	10.8388	-0.3503
ν_{16}	837.6	2.254e-09	1.255e-06	4.284e-05	15.3643	1.5555
ν_{17}	735.2	2.539e-07	3.017e-07	5.322e-06	57.2738	0.2079
ν_{18}	647.4	7.557e-08	-3.002e-07	-1.816e-05	5.1483	-0.6362
ν_{19}	492.7	1.370e-09	-7.200e-06	-3.214e-05	12.5748	-1.7940
ν_{20}	459.9	-8.829e-09	5.954e-06	2.744e-05	10.8816	1.5123
ν_{21}	174.2	-6.634e-10	-1.242e-08	5.161e-08	0.8173	0.0005

[a] Atomic units.
[b] Units of Å^{4}amu^{-1} for Raman, 10^{-3}Å^{4}amu^{-1} for VROA.

Table SI34: VROA results for (3,3)-d2-(4R)-d-2-oxetanone determined by CC2/asp-cc-pVDZ using a B3LYP/aug-cc-pVTZ geometry and Hessian.

		VROA Invariants[a]			SCP (180°)[b]	
Mode	ω/cm^{-1}	$\alpha G'$	$\beta(G')^2$	$\beta(A)^2$	$I_R + I_L$	$I_R - I_L$
ν_1	3110.4	3.169e-06	1.917e-05	-2.420e-04	494.4183	-6.1578
ν_2	2333.0	-9.839e-08	3.217e-06	2.098e-05	137.9989	1.0225
ν_3	2287.2	-2.233e-06	-2.282e-05	9.214e-05	292.3912	0.7909
ν_4	2247.9	2.139e-07	1.070e-05	1.852e-05	262.0715	1.6897
ν_5	1904.0	2.355e-08	1.132e-07	5.070e-06	154.3764	0.1806
ν_6	1373.7	1.341e-07	-1.564e-06	8.843e-06	10.8862	0.1386
ν_7	1289.0	-3.203e-09	3.463e-06	-3.254e-06	7.5455	0.2381
ν_8	1131.4	-3.595e-07	-2.801e-07	1.669e-05	46.5400	0.5292
ν_9	1112.3	-2.087e-09	1.738e-06	1.338e-06	7.2832	0.2187
ν_{10}	1054.1	9.467e-08	-4.581e-06	-2.401e-05	7.0278	-1.2601
ν_{11}	996.3	-8.233e-07	1.818e-06	1.332e-05	12.4722	0.6267
ν_{12}	940.6	1.080e-08	3.255e-06	4.023e-05	24.1131	1.6687
ν_{13}	923.3	8.224e-07	2.789e-06	1.786e-05	96.8074	0.8755
ν_{14}	906.2	-1.622e-07	-2.173e-06	-1.029e-06	4.5682	-0.2520
ν_{15}	881.2	-4.563e-08	-5.578e-06	-4.471e-05	19.7479	-2.0509
ν_{16}	801.1	2.268e-08	-1.163e-06	-1.814e-05	29.2702	-0.7219
ν_{17}	718.8	4.513e-08	1.836e-06	3.755e-06	36.8542	0.3092
ν_{18}	623.4	7.574e-08	1.329e-06	5.879e-06	5.0230	0.3293
ν_{19}	466.0	2.579e-09	-1.142e-06	-3.885e-06	19.7357	-0.2440
ν_{20}	442.9	-9.144e-10	4.037e-07	1.989e-06	3.9082	0.1068
ν_{21}	170.9	-5.230e-10	3.142e-08	3.334e-08	0.8130	0.0043

[a] Atomic units.
[b] Units of $\text{Å}^4\text{amu}^{-1}$ for Raman, $10^{-3}\text{Å}^4\text{amu}^{-1}$ for VROA.

Table SI35: VROA results for (3S)-d-(4,4)-d2-2-oxetanone determined by CC2/asp-cc-pVDZ using a B3LYP/aug-cc-pVTZ geometry and Hessian.

		VROA Invariants[a]			SCP (180°)[b]	
Mode	ω/cm^{-1}	$\alpha G'$	$\beta(G')^2$	$\beta(A)^2$	$I_R + I_L$	$I_R - I_L$
ν_1	3115.8	5.528e-06	8.376e-06	-1.903e-05	434.2176	0.2034
ν_2	2337.4	-8.405e-08	-3.576e-06	-9.169e-06	176.3566	-0.6642
ν_3	2289.8	-2.596e-06	7.146e-06	8.519e-05	253.5646	3.5592
ν_4	2238.9	1.911e-07	-7.777e-06	-4.326e-05	294.6494	-2.2227
ν_5	1903.5	1.931e-08	1.219e-06	-5.344e-06	146.9245	-0.0563
ν_6	1312.1	-8.115e-08	-3.419e-06	-6.607e-06	19.1590	-0.5630
ν_7	1200.5	8.440e-09	2.315e-06	1.183e-04	8.0433	4.1806
ν_8	1189.8	-1.166e-08	-3.029e-06	-8.937e-05	1.7495	-3.2865
ν_9	1145.5	6.471e-08	6.350e-08	-1.383e-05	27.1302	-0.4553
ν_{10}	1030.5	5.375e-07	7.408e-07	-2.804e-05	35.1013	-0.8619
ν_{11}	978.3	2.359e-07	5.277e-07	-1.698e-05	5.3092	-0.5140
ν_{12}	967.9	1.470e-07	6.581e-07	5.786e-06	18.7484	0.2590
ν_{13}	931.7	-1.151e-06	-1.821e-06	-2.356e-06	78.1012	-0.2610
ν_{14}	901.5	-2.985e-09	3.439e-06	-9.833e-06	17.4089	0.0162
ν_{15}	855.2	4.112e-08	3.418e-06	2.600e-05	9.9648	1.2101
ν_{16}	795.6	-3.967e-08	-7.257e-07	2.599e-05	26.7522	0.7948
ν_{17}	728.3	1.984e-07	-1.756e-06	3.102e-06	55.2235	-0.0723
ν_{18}	622.8	-2.129e-09	-1.700e-06	-2.109e-05	4.4334	-0.8743
ν_{19}	489.2	-2.867e-09	-6.446e-06	-3.041e-05	11.5360	-1.6605
ν_{20}	459.5	-7.896e-09	6.198e-06	2.882e-05	11.1734	1.5828
ν_{21}	167.0	9.839e-11	-2.212e-08	9.926e-08	1.1348	0.0011

[a] Atomic units.
[b] Units of $\mathrm{\AA}^4\mathrm{amu}^{-1}$ for Raman, $10^{-3}\mathrm{\AA}^4\mathrm{amu}^{-1}$ for VROA.

Table SI36: VROA results for (S)-methyloxirane determined by CC2/aug-cc-pVDZ using a B3LYP/aug-cc-pVTZ geometry and Hessian.

		VROA Invariants[a]			SCP (180°)[b]	
Mode	ω/cm^{-1}	$\alpha G'$	$\beta(G')^2$	$\beta(A)^2$	$I_R + I_L$	$I_R - I_L$
ν_1	3164.4	1.781e-06	3.571e-05	3.487e-04	359.9738	15.2150
ν_2	3109.5	-2.231e-06	3.255e-06	1.418e-04	128.0014	5.0582
ν_3	3088.4	-2.922e-06	-1.204e-04	-7.440e-04	881.7418	-36.8882
ν_4	3084.5	2.305e-07	1.845e-06	-1.804e-04	306.0426	-5.8371
ν_5	3081.0	2.372e-06	6.845e-05	1.788e-04	492.9010	12.8222
ν_6	3027.9	-2.050e-06	-3.124e-06	1.835e-05	815.7888	0.2996
ν_7	1529.2	-1.533e-08	-3.212e-06	-1.233e-05	15.9426	-0.7333
ν_8	1497.0	-6.920e-08	1.150e-05	4.977e-05	21.7177	2.8133
ν_9	1483.2	1.806e-09	-2.631e-06	-1.051e-05	15.5277	-0.6143
ν_{10}	1438.6	-2.066e-07	-5.793e-06	-2.967e-05	31.7384	-1.5703
ν_{11}	1406.7	9.124e-08	1.385e-06	5.943e-06	7.6368	0.3371
ν_{12}	1293.3	-5.035e-07	2.482e-06	5.755e-06	101.8742	0.4407
ν_{13}	1189.2	-5.992e-08	-6.500e-06	-4.900e-05	18.0834	-2.2864
ν_{14}	1166.8	-1.105e-07	1.160e-06	1.891e-05	7.1946	0.7474
ν_{15}	1156.9	-4.333e-08	4.953e-06	1.100e-05	14.9636	0.8632
ν_{16}	1129.2	-1.484e-07	-1.086e-06	-1.935e-05	8.6560	-0.7545
ν_{17}	1042.6	2.671e-07	2.907e-06	3.237e-05	6.8920	1.3716
ν_{18}	972.4	4.684e-07	1.731e-06	3.507e-06	33.8712	0.2904
ν_{19}	907.7	8.395e-08	-5.647e-06	-7.410e-05	15.8952	-3.0388
ν_{20}	842.2	-2.030e-08	-8.871e-06	-1.178e-06	38.4747	-0.9276
ν_{21}	770.4	2.119e-07	7.466e-06	3.995e-05	30.7322	2.0812
ν_{22}	410.2	-4.649e-08	-7.239e-07	-1.408e-08	4.1063	-0.0730
ν_{23}	367.1	1.006e-08	-7.266e-07	-2.668e-06	4.2849	-0.1618
ν_{24}	207.1	1.005e-08	1.136e-09	-1.080e-06	0.2428	-0.0359

[a] Atomic units.
[b] Units of Å^{4}amu^{-1} for Raman, 10^{-3}Å^{4}amu^{-1} for VROA.

Table SI37: VROA results for (S)-methyloxirane determined by CC2/aug-cc-pVDZ using a CCSD(T)/cc-pVTZ geometry and Hessian.

		VROA Invariants[a]			SCP (180°)[b]	
Mode	ω/cm^{-1}	$\alpha G'$	$\beta(G')^2$	$\beta(A)^2$	$I_R + I_L$	$I_R - I_L$
ν_1	3196.8	1.923e-06	3.973e-05	3.692e-04	360.5425	16.3036
ν_2	3134.6	-3.759e-06	-1.625e-05	1.620e-04	252.3640	3.7802
ν_3	3116.9	-1.408e-06	-8.917e-05	-7.824e-04	694.4651	-35.0463
ν_4	3112.2	6.351e-07	-2.751e-06	-1.309e-04	319.8700	-4.6456
ν_5	3105.5	2.068e-06	5.478e-05	1.175e-04	512.3722	9.4086
ν_6	3038.7	-2.182e-06	-2.546e-06	3.211e-05	829.1221	0.8169
ν_7	1541.4	-5.158e-08	-2.992e-06	-1.361e-05	16.6003	-0.7541
ν_8	1504.4	-6.599e-08	1.182e-05	5.119e-05	22.0569	2.8926
ν_9	1489.8	1.632e-10	-2.619e-06	-8.746e-06	15.6469	-0.5542
ν_{10}	1452.0	-2.285e-07	-6.704e-06	-2.497e-05	32.9026	-1.5050
ν_{11}	1402.5	1.092e-07	1.219e-06	4.555e-06	9.0211	0.2741
ν_{12}	1294.9	-4.596e-07	2.404e-06	6.413e-06	96.3897	0.4548
ν_{13}	1191.8	-1.229e-07	-7.060e-06	-5.851e-05	24.1868	-2.6601
ν_{14}	1171.2	-2.622e-08	8.093e-06	5.314e-05	11.4363	2.5842
ν_{15}	1161.6	-1.373e-07	-1.525e-06	-2.053e-05	11.0818	-0.8379
ν_{16}	1132.4	-1.051e-07	-3.404e-07	-1.532e-05	6.5088	-0.5455
ν_{17}	1046.1	3.056e-07	3.478e-06	3.999e-05	8.1663	1.6831
ν_{18}	978.9	4.893e-07	2.806e-07	-6.851e-06	37.8975	-0.2006
ν_{19}	908.0	5.346e-08	-3.982e-06	-7.177e-05	14.7129	-2.7945
ν_{20}	859.7	-2.946e-08	-9.835e-06	1.501e-06	37.3626	-0.9347
ν_{21}	777.0	2.083e-07	7.205e-06	3.610e-05	30.5158	1.9264
ν_{22}	405.9	-4.118e-08	-6.319e-07	8.430e-07	3.7298	-0.0351
ν_{23}	364.9	5.128e-09	-6.582e-07	-2.505e-06	4.1438	-0.1495
ν_{24}	213.1	1.319e-08	-3.411e-08	-9.484e-07	0.2844	-0.0351

[a] Atomic units.
[b] Units of $\text{Å}^4\text{amu}^{-1}$ for Raman, $10^{-3}\text{Å}^4\text{amu}^{-1}$ for VROA.

Table SI38: VROA results for (S)-methyloxirane determined by CCSD/aug-cc-pVDZ using a B3LYP/aug-cc-pVTZ geometry and Hessian.

		VROA Invariants[a]			SCP (180°)[b]	
Mode	ω/cm^{-1}	$\alpha G'$	$\beta(G')^2$	$\beta(A)^2$	$I_R + I_L$	$I_R - I_L$
ν_1	3164.4	1.303e-06	2.727e-05	2.587e-04	274.5244	11.3648
ν_2	3109.5	-1.534e-06	1.306e-05	1.895e-04	101.2613	7.6345
ν_3	3088.4	-2.477e-06	-1.013e-04	-6.495e-04	722.4457	-31.8241
ν_4	3084.5	2.805e-07	4.656e-06	-1.132e-04	260.6539	-3.3112
ν_5	3081.0	2.172e-06	5.616e-05	1.445e-04	405.4202	10.4474
ν_6	3027.9	-1.223e-06	-2.438e-06	9.972e-06	696.1129	0.0887
ν_7	1529.2	-4.395e-08	-2.707e-06	-1.028e-05	11.7099	-0.6142
ν_8	1497.0	-4.821e-08	1.105e-05	4.555e-05	18.7611	2.6269
ν_9	1483.2	6.665e-09	-2.711e-06	-1.049e-05	13.7356	-0.6215
ν_{10}	1438.6	-2.138e-07	-5.442e-06	-1.766e-05	26.6128	-1.1344
ν_{11}	1406.7	3.775e-08	8.844e-07	5.817e-06	5.5842	0.2827
ν_{12}	1293.3	-3.469e-07	3.264e-06	-3.959e-06	84.3494	0.1947
ν_{13}	1189.2	1.193e-07	-5.690e-06	-4.138e-05	14.0142	-1.9509
ν_{14}	1166.8	-6.864e-08	1.628e-06	1.717e-05	6.2812	0.7360
ν_{15}	1156.9	1.301e-08	4.433e-06	1.144e-05	11.1472	0.8258
ν_{16}	1129.2	-1.245e-07	-1.070e-06	-1.765e-05	6.5342	-0.6964
ν_{17}	1042.6	2.185e-07	1.919e-06	2.446e-05	5.4597	1.0086
ν_{18}	972.4	2.729e-07	2.283e-06	1.932e-06	28.1803	0.2931
ν_{19}	907.7	3.953e-08	-5.174e-06	-6.552e-05	12.7283	-2.7052
ν_{20}	842.2	-7.831e-08	-7.729e-06	-5.865e-06	33.6530	-0.9698
ν_{21}	770.4	1.886e-07	5.109e-06	4.331e-05	30.1992	1.9574
ν_{22}	410.2	-4.198e-08	-5.963e-07	-3.019e-07	3.6475	-0.0698
ν_{23}	367.1	7.910e-09	-9.150e-07	-2.229e-06	3.4018	-0.1660
ν_{24}	207.1	5.029e-09	5.405e-09	-7.095e-07	0.1836	-0.0231

[a] Atomic units.
[b] Units of Å^{4}amu^{-1} for Raman, 10^{-3}Å^{4}amu^{-1} for VROA.

Table SI39: VROA results for (S)-methyloxirane determined by CCSD/aug-cc-pVDZ using a CCSD(T)/cc-pVTZ geometry and Hessian.

		VROA Invariants[a]			SCP (180°)[b]	
Mode	ω/cm^{-1}	$\alpha G'$	$\beta(G')^2$	$\beta(A)^2$	$I_R + I_L$	$I_R - I_L$
ν_1	3196.8	1.416e-06	3.048e-05	2.744e-04	275.1470	12.2113
ν_2	3134.6	-2.718e-06	-6.806e-07	2.149e-04	195.3082	7.1036
ν_3	3116.9	-1.324e-06	-7.806e-05	-6.901e-04	574.9431	-30.8515
ν_4	3112.2	6.139e-07	9.176e-07	-6.799e-05	272.0284	-2.1778
ν_5	3105.5	1.921e-06	4.545e-05	9.407e-05	422.3090	7.6913
ν_6	3038.7	-1.330e-06	-1.966e-06	1.982e-05	707.1077	0.4646
ν_7	1541.4	-7.567e-08	-2.488e-06	-1.098e-05	12.0708	-0.6158
ν_8	1504.4	-4.510e-08	1.137e-05	4.688e-05	19.0229	2.7035
ν_9	1489.8	4.862e-09	-2.591e-06	-9.016e-06	13.8179	-0.5604
ν_{10}	1452.0	-2.234e-07	-6.256e-06	-1.476e-05	27.5522	-1.1191
ν_{11}	1402.5	4.670e-08	7.118e-07	4.537e-06	6.6084	0.2227
ν_{12}	1294.9	-3.181e-07	3.104e-06	-2.905e-06	80.0338	0.2138
ν_{13}	1191.8	1.205e-07	-6.157e-06	-4.890e-05	18.4025	-2.2487
ν_{14}	1171.2	-3.936e-08	7.851e-06	4.726e-05	10.5689	2.3636
ν_{15}	1161.6	-4.517e-08	-1.580e-06	-1.709e-05	7.3597	-0.7286
ν_{16}	1132.4	-9.736e-08	-3.220e-07	-1.383e-05	5.2008	-0.4940
ν_{17}	1046.1	2.470e-07	2.460e-06	3.169e-05	6.4409	1.3041
ν_{18}	978.9	2.760e-07	1.188e-06	-7.381e-06	31.6895	-0.1274
ν_{19}	908.0	2.380e-08	-3.950e-06	-6.296e-05	11.7059	-2.4970
ν_{20}	859.7	-8.612e-08	-8.478e-06	-4.798e-06	32.7762	-1.0091
ν_{21}	777.0	1.820e-07	4.801e-06	4.057e-05	30.0363	1.8350
ν_{22}	405.9	-3.884e-08	-5.274e-07	3.907e-07	3.3241	-0.0398
ν_{23}	364.9	4.267e-09	-8.448e-07	-2.188e-06	3.3009	-0.1576
ν_{24}	213.1	6.851e-09	-2.642e-08	-5.916e-07	0.2148	-0.0224

[a] Atomic units.
[b] Units of $\mathrm{\AA}^4\mathrm{amu}^{-1}$ for Raman, $10^{-3}\mathrm{\AA}^4\mathrm{amu}^{-1}$ for VROA.
